# Synthesis, Biological Evaluation, and Molecular Dynamics Studies of Novel 1,2,4-Triazole-Functionalized Benzene-Sulfonate Derivatives as Potential Enzyme Inhibitors and Cytotoxicity in HepG2 Cells

**DOI:** 10.3390/ph19101645

**Published:** 2026-10-19

**Authors:** Yasemin Ünver, Fikret Türkan, Halil İbrahim Güler, Ercan Oguz, Tuğba Usta, Fatih Çelik

**Affiliations:** 1 Department of Chemistry, Faculty of Sciences, Karadeniz Technical University, 61080 Trabzon, Türkiye; 2 Department of Basic Sciences, Faculty of Dentistry, Iğdır University, 76000 Iğdır, Türkiye; fikret.turkan@gmail.com; 3 Department of Basic Medical Science, Medical of Faculty, Nakhchivan State University, AZ 7012 Nakhchivan, Azerbaijan; 4 Department of Molecular Biology and Genetics, Faculty of Science, Karadeniz Technical University, 61080 Trabzon, Türkiye; 5 Department of Medical Services and Techniques, Health Services Vocational School, Iğdır University, 76000 Iğdır, Türkiye; 6 Department of Chemistry, Graduate School of Natural and Applied Science, Karadeniz Technical University, 61080 Trabzon, Türkiye

**Keywords:** cytotoxicity study, AChE, GST, enzyme inhibition, molecular docking, molecular dynamics

## Abstract

**Background/Objectives:** In this study, novel benzenesulfonate derivatives containing a 1,2,4-triazole ring were synthesized. **Methods:** Their molecular structures were characterized by IR, ^1^H NMR, and ^13^C NMR spectroscopy, together with Q-TOF LC/MS analyses. For compounds **5b** and **5d**, the available mass spectrometric data were not used for structural confirmation, and their structural assignments were based primarily on the spectroscopic data. The inhibitory effects of the synthesized compounds (**3**–**5**) against acetylcholinesterase (AChE) and glutathione S-transferase (GST) enzymes were evaluated. **Results:** Comparison of the results with the reference inhibitors, donepezil and ethacrynic acid, showed that compound **3e** exhibited the highest AChE inhibitory activity among the tested compounds, with an IC_50_ value of 1.60 ± 0.198 μM. In addition, compound 4a showed remarkable GST inhibitory activity, with an IC_50_ value of 1.95 ± 0.492 μM, demonstrating greater potency than the reference inhibitor. The cytotoxic effects of the selected compounds were further assessed against the HepG2 cell line after 24 and 48 h of treatment. Overall, the synthesized molecules exhibited potent enzyme inhibitory activities together with promising cytotoxic effects, highlighting their potential as lead candidates for further biological investigations. In silico analyses were performed to elucidate the molecular basis of the experimentally observed acetylcholinesterase (AChE) inhibitory activity of the synthesized derivatives. Molecular docking against human AChE (PDB ID: 4EY7) identified compound **3e** as the most favorable binder among the synthesized series, while **3f** also exhibited a strong binding profile. Subsequent 100 ns molecular dynamics simulations of the hAChE–**3e**, hAChE–**3f**, and hAChE–donepezil complexes demonstrated stable protein conformations and persistent ligand accommodation within the catalytic gorge. MM/GBSA calculations further indicated a more favorable binding-energy profile for **3e** than for **3f**, whereas donepezil showed the most favorable overall calculated binding energy. Per-residue decomposition highlighted key gorge residues, including Trp86, Trp286, Tyr337, Tyr341, Phe295, and Phe297, as important contributors to ligand stabilization. **Conclusions:** Collectively, these computational findings provide structural and energetic support for the experimentally observed AChE inhibitory activity of the lead derivatives.

## 1. Introduction

Nitrogen-containing heterocycles, particularly 1,2,4-triazole derivatives, have attracted considerable attention because of their broad utility in medicinal chemistry, agricultural applications, materials development, and organic synthesis. Their significance in drug design is largely attributed to the ability of the triazole ring to establish favorable interactions with biological targets, including hydrogen bonding and dipole–dipole interactions, which can contribute to molecular recognition and biological activity. The antimicrobial activities of compounds containing the 1,2,4-triazole moiety are well documented, and several commercially available drugs, including Fluconazole, Flupoxam, and Anastrozole, incorporate this heterocyclic scaffold [1,2,3,4,5,6]. The biological significance of 1,2,4-triazoles has stimulated the development of numerous synthetic methodologies for the construction of this framework. The synthetic approaches reported to date provide access to a wide variety of 1,2,4-triazole derivatives through multistep synthetic routes [7,8,9,10,11]. Due to their chemotherapeutic potential and diagnostic applications, 1,2,4-triazoles constitute one of the most important and widely utilized classes of heterocyclic compounds in organic and medicinal chemistry. Previous studies have demonstrated that molecules containing a 1,2,4-triazole ring exhibit remarkable anti-inflammatory, anticancer, antihypertensive, antimicrobial, and anticonvulsant activities, among others [12,13,14,15,16,17,18,19,20]. Although numerous 1,2,4-triazole-containing compounds have already been reported, these molecules continue to attract considerable attention from researchers because of their broad spectrum of biological activities, low toxicity, and favorable pharmacokinetic properties. Consequently, the synthesis of novel analogs bearing various substituents remains an active area of research [21]. Sulfonates are among the most frequently employed leaving groups owing to their nucleophilic properties, ease of preparation, and favorable reaction rates. Sulfonic esters are widely utilized as intermediates in numerous synthetic transformations. The reactions of sulfonate esters have played a pivotal role in the development of many fundamental concepts of modern chemistry, including reaction mechanisms, neighboring-group participation, nonclassical carbocations, solvent effects on reactivity, and linear free-energy relationships [22]. Molecules containing sulfonate groups have been reported to exhibit enhanced intracellular activities [23]. Furthermore, these derivatives possess superior antitumor, anticoagulant, and antibacterial properties [24]. Schiff bases, characterized by the presence of an azomethine (–CH=N–) linkage, represent an important class of organic compounds with a wide range of applications in medicinal chemistry and various industrial fields [25]. These compounds have attracted significant interest due to their diverse pharmacological potential, including antibacterial [26], antifungal [27], anticancer, anti-HIV [28], antitubercular [29], antiviral [30], anti-inflammatory [31], anticonvulsant [32], and antimalarial activities [33]. Recent studies by Ünver et al. have focused on the synthesis and biological evaluation of sulfonate derivatives incorporating triazole–hydrazone moieties. These compounds exhibited notable antimicrobial and anticancer activities, while DFT calculations and molecular docking studies provided insights into their structural and molecular properties [34]. In a subsequent study, related triazole–hydrazone sulfonate derivatives were further investigated through experimental, DFT, and in silico approaches, demonstrating the potential of this scaffold for biological applications [35].

Among the molecular targets implicated in neurodegenerative disorders, acetylcholinesterase (AChE; EC 3.1.1.7) remains one of the most extensively investigated enzymes due to its central role in cholinergic neurotransmission [36]. Progressive degeneration of cholinergic neurons, particularly in Alzheimer’s disease (AD), leads to a marked decline in acetylcholine (ACh) levels within the synaptic cleft, resulting in impairments of learning, memory, and other cognitive functions [37]. According to the cholinergic hypothesis, pharmacological inhibition of AChE prolongs the availability of ACh at cholinergic synapses, thereby partially restoring neurotransmission and improving cognitive performance [38]. Structurally, AChE is a serine hydrolase characterized by a narrow catalytic gorge containing the catalytic triad (Ser203, His447, and Glu334), an anionic binding site, and a peripheral anionic site (PAS). Beyond its classical role in terminating cholinergic signaling through rapid hydrolysis of acetylcholine, accumulating evidence indicates that AChE actively contributes to AD pathology by promoting amyloid-β (Aβ) fibrillogenesis through interactions at the PAS [39]. Consequently, the development of multifunctional AChE inhibitors capable of targeting both the catalytic active site and the PAS has emerged as an attractive therapeutic strategy with the potential not only to alleviate cognitive symptoms but also to interfere with disease progression [40].

In parallel, glutathione S-transferases (GSTs; EC 2.5.1.18) constitute another therapeutically relevant enzyme family involved in the cellular defense against oxidative and electrophilic stress [41]. These phase II detoxification enzymes catalyze the conjugation of reduced glutathione (GSH) with a wide range of endogenous and exogenous electrophilic compounds, thereby facilitating their detoxification and elimination [42]. Although this protective mechanism is essential for maintaining cellular homeostasis under physiological conditions, aberrant overexpression of GST isoenzymes has been reported in numerous malignancies, including hepatocellular carcinoma, where it contributes to enhanced detoxification of anticancer drugs, resistance to chemotherapy, tumor progression, and unfavorable clinical outcomes. The human hepatocellular carcinoma cell line HepG2 is widely recognized as an appropriate in vitro model for evaluating GST inhibitors and anticancer candidates because it retains many hepatic metabolic characteristics and exhibits substantial GST activity [43]. Inhibition of GST activity in HepG2 cells disrupts intracellular redox homeostasis, promotes the accumulation of reactive oxygen species (ROS), impairs mitochondrial function, and ultimately triggers apoptotic cell death [44]. Therefore, selective GST inhibition represents a promising strategy for sensitizing tumor cells to oxidative damage and improving the therapeutic efficacy of anticancer agents.

Although AChE and GST participate in distinct physiological pathways, both enzymes represent validated molecular targets in the treatment of complex human diseases. Whereas AChE inhibition primarily aims to preserve cholinergic neurotransmission and mitigate amyloid-associated neurotoxicity in neurodegenerative disorders, GST inhibition is intended to weaken the antioxidant defense system of cancer cells, thereby increasing their susceptibility to oxidative stress and chemotherapeutic agents [45]. Consequently, the identification of novel heterocyclic scaffolds exhibiting **GST inhibitory activity together with cytotoxic effects in HepG2 cells provides a relevant basis for investigating the relationship between GST-associated biochemical activity and cellular responses in a hepatocellular carcinoma model. In addition, the AChE inhibitory activity of these compounds was investigated as a complementary biochemical property, considering the reported involvement of AChE-related pathways in different cancer cell models. Thus, the present study primarily focuses on the relationship between GST inhibition and cytotoxicity in HepG2 cells, while AChE inhibition provides additional information on the multifunctional biochemical profile of the synthesized compounds. In this study, a new series of sulfonate derivatives bearing 1,2,4-triazole–Schiff base moieties was synthesized. A systematic structure–activity relationship (SAR) approach was adopted by introducing different substituents at the para position of the aromatic ring, with H serving as the reference group. Electron-donating (p-OCH_3_) and electron-withdrawing (p-F, p-Cl, p-Br, p-I, and p-NO_2_) substituents were selected to investigate the influence of their electronic properties on the molecular and biological behavior of the derivatives. The differences in size and polarizability among the halogen substituents were also considered to evaluate their contribution to molecular interactions and biological activity. The synthesized compounds were fully characterized, and their biological potential was systematically evaluated through AChE and GST inhibition assays and cytotoxicity studies against HepG2 cells. Molecular docking and subsequent molecular dynamics analyses were additionally performed for human AChE to provide a structural interpretation of the experimentally observed AChE inhibitory activity. GST inhibition was evaluated experimentally, and no isoenzyme-specific structural mechanism is inferred from the present data.

## 2. Results and Discussion

### 2.1. Synthesis of 1,2,4-Triazol-Phenyl Benzenesulfonate (***3***–***5***)

**Scheme 1:**
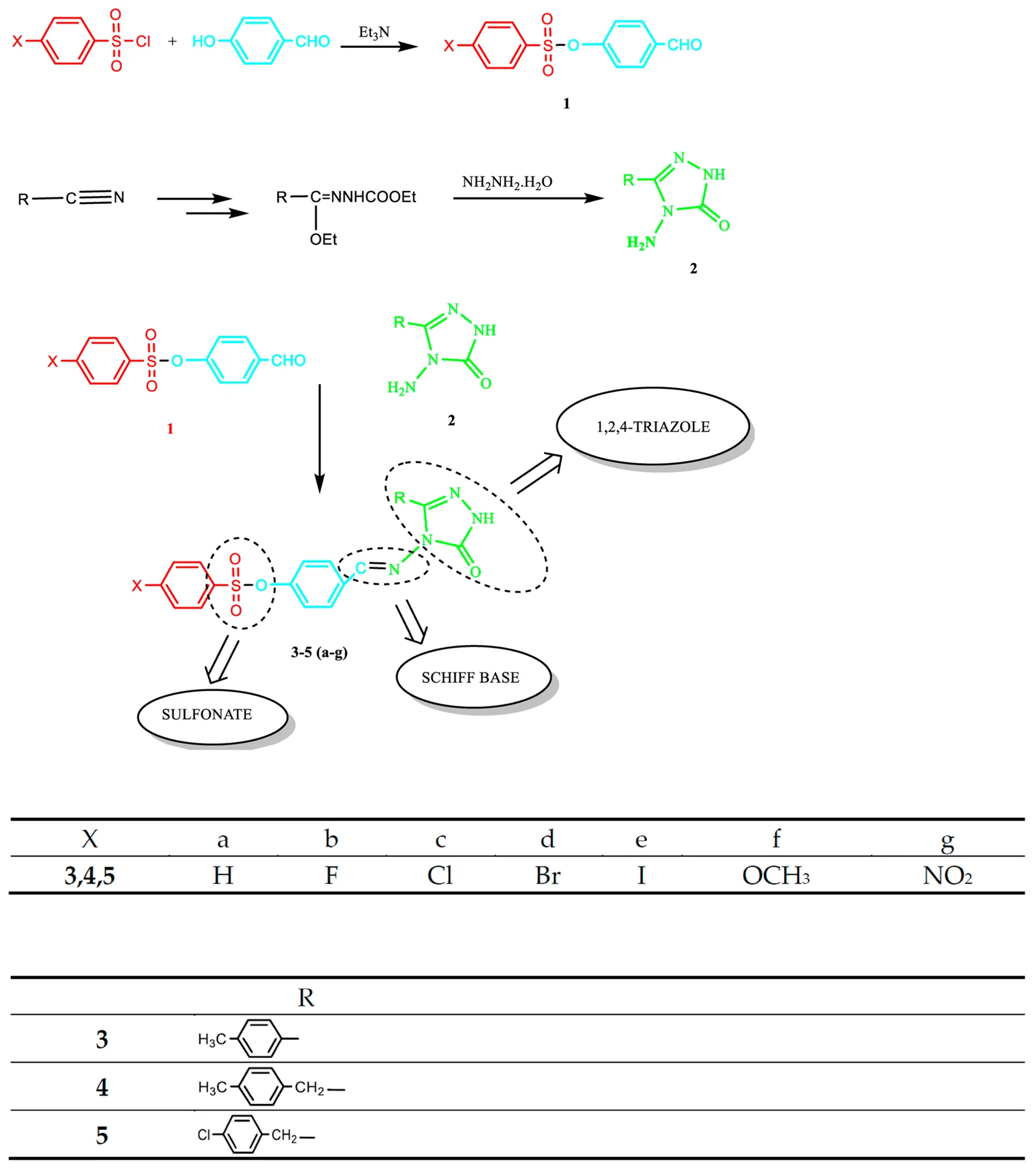
Synthetic pathway for the preparation of compounds **3**–**5** (a–g).

A solvent-free condensation reaction was carried out by combining 4-formylphenyl 4-substituted-benzenesulfonates (**1**) (0.01 mol) with 4-amino-5-substituted-3H-1,2,4-triazol-3-ones (**2**) (0.01 mol). The reaction mixture was heated at 160–170 °C in an oil bath for 1 h, with the reaction progress followed by thin-layer chromatography (TLC). Upon completion, the mixture was allowed to cool to room temperature, and the precipitated product was collected by filtration. The crude product was subsequently purified through recrystallization using a DMF/water mixture (1:3, *v*/*v*), yielding the target compounds (**3**–**5**), as illustrated in Scheme 1. The starting materials (**1** and **2**) were prepared following previously reported procedures [46,47].

(E)-4-(((5-oxo-3-(p-tolyl)-1,5-dihydro-4H-1,2,4-triazol-4-yl) imino) methyl) phenyl benzene sulfonate (**3a**): Yield 80.80%, m.p. 74–74 °C. IR (KBr, cm^−1^): 3184 (NH), 3071 (=CH), 1700 (C=O), 1600 (C=N), 1504 (C=C); ^1^H NMR (400 MHz, DMSO-d_6_) δ: 2.37 (s, CH_3_, 3H), benzene H [7.18 (d, 2H, J = 8 Hz), 7.33 (d, 2H, J = 8 Hz), 7.67–7.71 (m, 2H), 7.74–7.76 (m, 2H), 7.81–7.84 (m, 3H), 7.89–7.91 (m, 2H)], 9.65 (s, N=CH, 1H), 12.36 (s, NH, 1H); ^13^C NMR (100 MHz, DMSO-d_6_) δ: 21.47 (CH_3_), benzene C [123.32 (2CH), 124.18 (C), 128.38 (2CH), 128.72 (2CH), 129.60 (CH), 130.06 (2CH), 130.41 (2CH), 133.03 (C), 134.56 (C), 135.74 (CH),140.39 (C)] 151.37 (C=N triazol), 151.72 (C=O), 155.15 (N=CH). LC Q-TOF *m*/*z*: [M+H]^+^ calcd for C_22_H_18_N_4_O4S, 435.11216; found, 435.11303.

(E)-4-(((5-oxo-3-(p-tolyl)-1,5-dihydro-4H-1,2,4-triazol-4-yl) imino) methyl) phenyl 4-fluoro benzene sulfonate (**3b**): Yield 85.60%, m.p. 193–195 °C. IR (KBr, cm^−1^): 3168 (NH), 3043 (=CH), 1697 (C=O), 1588 (C=N), 1504 (C=C); ^1^H NMR (400 MHz, DMSO-d_6_) δ: 2.37 (s, CH_3_, 3H), benzene H [7.30 (d, 2H, J = 8 Hz), 7.33 (d, 2H, J = 8 Hz), 7.54 (t, 2H, J = 8Hz), 7.76 (d, 2H, J = 8 Hz), 7.84 (d, 2H, J = 8 Hz), 7.96–8.00 (m, 2H)], 9.66 (s, N=CH, 1H), 12.36 (s, NH, 1H); ^13^C NMR (100 MHz, DMSO-d_6_) δ: 21.45 (CH_3_), benzene C [117.70,117.93 (2CH), 123.41 (2CH) 124.18 (C), 128.38 (2CH), 129.61 (2CH), 130.12 (2CH), 130.74, 130.77 (2C), 132.12,132.22 (2CH), 133.09 (C), 140.40 (C), 145.17 (C), 164.88, 167.42 (C)] 151.28 (C=N triazol), 151.72 (C=O), 155.15 (N=CH). LC Q-TOF *m*/*z*: [M+H]^+^ calcd for C_22_H_17_FN_4_O4S, 453.10273; found, 453.10310.

(E)-4-(((5-oxo-3-(p-tolyl)-1,5-dihydro-4H-1,2,4-triazol-4-yl) imino) methyl) phenyl 4-chloro benzene sulfonate (**3c**): Yield 81.20%, m.p. 227–229 °C. IR (KBr, cm^−1^): 3168 (NH), 3064 (=CH), 1694 (C=O), 1584 (C=N), 1505 (C=C); ^1^H NMR (400 MHz, DMSO-d_6_) δ: 2.37 (s, CH_3_, 3H), benzene H [7.21 (d, 2H, J = 8 Hz), 7.30 (d, 2H, J = 8 Hz), 7.75–7.78 (m, 2H), 7.84 (d, 2H, J = 8 Hz), 7.90 (d, 2H, J = 8 Hz), 7.96–8.00 (m, 2H)], 9.66 (s, N=CH, 1H), 12.36 (s, NH, 1H); ^13^C NMR (100 MHz, DMSO-d_6_) δ: 21.45 (CH_3_), benzene C [123.39 (2CH), 124.18 (C), 128.38 (2CH), 129.60 (2CH), 130.15 (2CH), 130.62 (CH), 130.70 (CH), 132.24 (C), 133.29 (C), 140.39 (C), 140.77 (C), 145.17 (C)] 151.23 (C=N triazol), 151.72 (C=O), 155.09 (N=CH). LC Q-TOF *m*/*z*: [M+H]^+^ calcd for C_22_H_17_ClN_4_O4S, 469.07318; found, 469.07497.

(E)-4-(((5-oxo-3-(p-tolyl)-1,5-dihydro-4H-1,2,4-triazol-4-yl) imino) methyl) phenyl 4-bromo benzene sulfonate (**3d**): Yield 88.50%, m.p. 232–234 °C. IR (KBr, cm^−1^): 3166 (NH), 3061 (=CH), 1695 (C=O), 1574 (C=N), 1506 (C=C); ^1^H NMR (400 MHz, DMSO-d_6_) δ: 2.37 (s, CH_3_, 3H), benzene H [7.21 (d, 2H, J = 8 Hz), 7.34 (d, 2H, J = 8 Hz), 7.80–7.86 (m, 4H), 7.90–7.92 (d, 2H, J = 8 Hz), 7.96–8.00 (m, 2H)], 9.66 (s, N=CH, 1H), 12.36 (s, NH, 1H); ^13^C NMR (100 MHz, DMSO-d_6_) δ: 21.46 (CH_3_), benzene C [123.39 (2CH), 124.18 (C), 128.39 (2CH), 129.61 (2CH), 129.98 (C), 130.16 (2CH), 130.66 (2CH), 133.24 (C), 133.56 (2CH), 133.72 (C), 140.40 (C), 145.18 (C)] 151.24 (C=N triazol), 151.72 (C=O), 155.12 (N=CH). LC Q-TOF *m*/*z*: [M+H]^+^ calcd for C_22_H_17_BrN_4_O4S, 513.02267; found, 513.02430.

(E)-4-(((5-oxo-3-(p-tolyl)-1,5-dihydro-4H-1,2,4-triazol-4-yl)imino)methyl)phenyl 4-iodo benzene sulfonate (**3e**): Yield 84.45%, m.p. 234–236 °C. IR (KBr, cm^−1^): 3164 (NH), 3056 (=CH), 1693 (C=O), 1569 (C=N), 1505 (C=C); ^1^H NMR (400 MHz, DMSO-d_6_) δ: 2.37 (s, CH_3_, 3H), benzene H [7.21 (d, 2H, J = 8 Hz), 7.34 (d, 2H, J = 8 Hz), 7.63 (d, 2H, J = 8 Hz), 7.76 (d, 2H, J = 8 Hz), 7.84 (d, 2H, J = 8 Hz), 8.07 (d, 2H, J = 8 Hz)], 9.66 (s, N=CH, 1H), 12.36 (s, NH, 1H); ^13^C NMR (100 MHz, DMSO-d_6_) δ: 21.16 (CH_3_), benzene C [123.28 (2CH), 124.11 (C), 128.39 (2CH), 129.61 (2CH), 130.12 (2CH), 133.20 (C), 134.05 (C), 139.33 (2CH), 140.30 (C), 145.17 (C)] 151.26 (C=N triazol), 151.72 (C=O), 155.13 (N=CH). LC Q-TOF *m*/*z*: [M+H]^+^ calcd for C_22_H_17_IN_4_O4S, 561.00880; found, 561.01090.

(E)-4-(((5-oxo-3-(p-tolyl)-1,5-dihydro-4H-1,2,4-triazol-4-yl) imino) methyl) phenyl 4-methoxy benzene sulfonate (**3f**): Yield 81.70%, m.p. 221–223 °C. IR (KBr, cm^−1^): 3181 (NH), 3076 (=CH), 1705 (C=O), 1595 (C=N), 1510 (C=C), 1149 (C-O-C); ^1^H NMR (400 MHz, DMSO-d_6_) δ: 2.37 (s, CH_3_, 3H), 3.87 (OCH_3_), benzene H [7.16–7.19 (m, 4H), 7.34 (d, 2H, J = 8 Hz), 7.75–7.84 (m, 6H)], 9.64 (s, N=CH, 1H), 12.36 (s, NH, 1H); ^13^C NMR (100 MHz, DMSO-d_6_) δ: 21.73 (CH_3_), 56.06 (OCH_3_), benzene C [115.54 (2CH), 123.30 (2CH), 124.14 (C), 125.63 (C), 128.29 (2CH), 129.56 (2CH), 130.03 (2CH), 131.18 (C), 134.05 (2CH), 132.92 (C), 140.39 (C), 145.16 (C), 164.61 (C)] 151.52 (C=N triazol), 151.77 (C=O), 155.25 (N=CH). LC Q-TOF *m*/*z*: [M+H]^+^ calcd for C_23_H_20_N_4_O5S, 465.12272; found, 465.12442.

(E)-4-(((5-oxo-3-(p-tolyl)-1,5-dihydro-4H-1,2,4-triazol-4-yl)imino) methyl) phenyl 4-nitro benzene sulfonate (**3g**): Yield 89.15%, m.p. 265–267 °C. IR (KBr, cm^−1^): 3169 (NH), 3066 (=CH), 1696 (C=O), 1605 (C=N), 1585 (C=C); ^1^H NMR (400 MHz, DMSO-d_6_) δ: 2.37 (s, CH_3_, 3H), benzene H [7.23 (d, 2H, J = 8 Hz), 7.33 (d, 2H, J = 8 Hz), 7.75 (d, 2H, J = 8 Hz), 7.84 (d, 2H, J = 8 Hz), 8.18 (d, 2H, J = 8 Hz), 8.46 (d, 2H, J = 8 Hz)], 9.67 (s, N=CH, 1H), 12.36 (s, NH, 1H); ^13^C NMR (100 MHz, DMSO-d_6_) δ: 21.17 (CH_3_), benzene C [123.39 (2CH), 124.16 (C), 125.60 (2CH), 128.39 (2CH), 129.59 (2CH), 130.23 (2CH), 130.56 (2CH), 133.45 (C), 139.67 (C), 140.44 (C), 145.18 (C), 151.05 (C)] 151.62 (C=N triazol), 151.71 (C=O), 154.98 (N=CH). LC Q-TOF *m*/*z*: [M+H]^+^ calcd for C_22_H_17_N_5_O_6_S, 480.09723; found, 480.0988.

(E)-4-(((3-(4-methylbenzyl)-5-oxo-1,5-dihydro-4H-1,2,4-triazol-4-yl)imino)methyl)phenyl benzenesulfonate (**4a**): Yield 90.30%, m.p. 135–137 °C. IR (KBr, cm^−1^): 3190 (NH), 3062 (=CH), 1707 (C=O), 1590 (C=N), 1503 (C=C); ^1^H NMR (400 MHz, DMSO-d_6_) δ: 2.24 (s, CH_3_, 3H), 3.93 (s, CH_2_, 2H), benzene H [7.18 (bs, 7H), 7.89 (bs, 6H)], 9.67 (s, N=CH, 1H), 12.02 (s, NH, 1H); ^13^C NMR (100 MHz, DMSO-d_6_) δ: 21.15 (CH_3_), 30.95 (CH_2_), benzene C [123. 22 (2CH), 128.75 (2CH), 129.13 (2CH), 129.49 (2CH), 129.87 (2CH), 130.23 (2CH), 130.40 (2CH), 133.08 (C), 133.23 (C), 134.50 (C), 135.73 (CH), 136.26 (C), 146.90 (C)] 151.22 (C=N triazol), 151.55 (C=O), 152.22 (N=CH). LC Q-TOF *m*/*z*: [M+H]^+^ calcd for C_23_H_20_N_4_O_4_S, 449.12781; found, 449.12804.

(E)-4-(((3-(4-methylbenzyl)-5-oxo-1,5-dihydro-4H-1,2,4-triazol-4-yl)imino)methyl)phenyl 4-fluoro benzene sulfonate (**4b**): Yield 91.45%, m.p. 180–182 °C. IR (KBr, cm^−1^): 3174 (NH), 3071 (=CH), 1704 (C=O), 1591 (C=N), 1492 (C=C); ^1^H NMR (400 MHz, DMSO-d_6_) δ: 2.24 (s, CH_3_, 3H), 3.98 (s, CH_2_, 2H), benzene H [7.20 (bs, 6H), 7.56 (bs, 2H), 7.84–7.98(m, 4H)], 9.69 (s, N=CH, 1H), 12.00 (s, NH, 1H); ^13^C NMR (100 MHz, DMSO-d_6_) δ: 20.66 (CH_3_), 31.24 (CH_2_), benzene C [117.67, 117.90 (2CH), 123.30 (2CH), 129.13 (2CH), 129.49 (2CH), 129.92 (2CH), 130.75 (C), 132.16, 132.26 (2CH), 133.08,133.33 (C), 136.26 (C), 146.84 (C), 164.88, 167.42 (C)] 151.12 (C=N triazol), 151.57 (C=O), 152.19 (N=CH). LC Q-TOF *m*/*z*: [M+H]^+^ calcd for C_23_H_19_FN_4_O_4_S, 467.11838; found, 467.11986.

(E)-4-(((3-(4-methylbenzyl)-5-oxo-1,5-dihydro-4H-1,2,4-triazol-4-yl)imino)methyl)phenyl 4-chlorobenzenesulfonate (**4c**): Yield 89.90%, m.p. 170–172 °C. IR (KBr, cm^−1^): 3174 (NH), 3092 (=CH), 1690 (C=O), 1590 (C=N), 1504 (C=C); ^1^H NMR (400 MHz, DMSO-d_6_) δ: 2.24 (s, CH_3_, 3H), 3.98 (s, CH_2_, 2H), benzene H [7.10 (d, 2H, J = 8 Hz), 7.18 (d, 4H, J = 8 Hz), 7.77 (d, 2H, J = 8 Hz), 7.81 (d, 2H, J = 8 Hz), 7.90 (d, 2H, J = 8 Hz)], 9.67 (s, N=CH, 1H), 12.00 (s, NH, 1H); ^13^C NMR (100 MHz, DMSO-d_6_) δ: 20.87 (CH_3_), 30.98 (CH_2_), benzene C [123.98 (2CH), 129.13 (2CH), 129.48 (2CH), 129.95 (2CH), 130.59 (2CH), 130.73 (2CH), 133.08 (C), 133.26 (C), 133. 39 (C), 136.26 (C), 140.76 (C), 146.84 (C)] 151.06 (C=N triazol), 151.52 (C=O), 152.15 (N=CH). LC Q-TOF *m*/*z*: [M+H]^+^ calcd for C_23_H_19_ClN_4_O_4_S, 483.08883; found, 483.09053.

(E)-4-(((3-(4-methylbenzyl)-5-oxo-1,5-dihydro-4H-1,2,4-triazol-4-yl)imino)methyl)phenyl 4-bromobenzenesulfonate (**4d**):Yield 86.46%, m.p. 163–165 °C. IR (KBr, cm^−1^): 3157 (NH), 3066 (=CH), 1697 (C=O), 1597 (C=N), 1573 (C=C); ^1^H NMR (400 MHz, DMSO-d_6_) δ: 2.25 (s, CH_3_, 3H), 3.99 (s, CH_2_, 2H), benzene H [7.11–7.20 (m, 6H), 7.82–7.90 (m, 6H)], 9.67 (s, N=CH, 1H), 12.00 (s, NH, 1H); ^13^C NMR (100 MHz, DMSO-d_6_) δ: 20.65 (CH_3_), 30.96 (CH_2_), benzene C [123.29 (2CH), 129.14 (2CH), 129.49 (2CH), 129.96 (2CH), 129.99 (C), 130.70 (2CH), 133.09 (C), 133.40 (C), 133.54 (CH), 133. 68 (CH), 136.23 (C), 146.85 (C)] 151.08 (C=N triazol), 151.62 (C=O), 152.11 (N=CH). LC Q-TOF *m*/*z*: [M+H]^+^ calcd for C_23_H_19_BrN_4_O_4_S, 527.03832; found, 527.04042.

(E)-4-(((3-(4-methylbenzyl)-5-oxo-1,5-dihydro-4H-1,2,4-triazol-4-yl)imino) methyl) phenyl 4-iodo benzene sulfonate (**4e**): Yield 88.60%, m.p. 196–198 °C. IR (KBr, cm^−1^): 3163 (NH), 3034 (=CH), 1693 (C=O), 1597 (C=N), 1567 (C=C); ^1^H NMR (400 MHz, DMSO-d_6_) δ: 2.25 (s, CH_3_, 3H), 3.98 (s, CH_2_, 2H), benzene H [7.11–7.18 (m, 6H), 7.62 (d, 2H, J = 8 Hz), 7.81 (d, 2H, J = 8 Hz), 8.05 (d, 2H, J = 8 Hz)], 9.66 (s, N=CH, 1H), 12.00 (s, NH, 1H); ^13^C NMR (100 MHz, DMSO-d_6_) δ: 20.87 (CH_3_), 30.96 (CH_2_), benzene C [123.26 (2CH), 129.13 (2CH), 129.49 (2CH), 129.94 (2CH), 130.16 (CH), 133.09 (C), 133.36 (C), 134.01 (C), 136.26 (C), 139. 30 (2CH), 146.84 (C)] 151.16 (C=N triazol), 151.62 (C=O), 152.13 (N=CH). LC Q-TOF *m*/*z*: [M+H]^+^ calcd for C_23_H_19_IN_4_O_4_S, 575.02445; found, 575.02561.

(E)-4-(((3-(4-methylbenzyl)-5-oxo-1,5-dihydro-4H-1,2,4-triazol-4-yl)imino)methyl)phenyl 4-methoxybenzenesulfonate (**4f**): Yield 90.50%, m.p. 174–176 °C. IR (KBr, cm^−1^): 3183 (NH), 3063 (=CH), 1709 (C=O), 1595 (C=N), 1577 (C=C); ^1^H NMR (400 MHz, DMSO-d_6_) δ: 2.25 (s, CH_3_, 3H), 3.88 (s, OCH_3_, 3H),3.98 (s, CH_2_, 2H), benzene H [7.17 (bs, 8H), 7.82 (bs, 4H)], 9.64 (s, N=CH, 1H), 12.00 (s, NH, 1H); ^13^C NMR (100 MHz, DMSO-d_6_) δ: 20.66 (CH_3_), 30.95 (CH_2_), 56.38 (OCH_3_), benzene C [115.51 (2CH), 123.18 (2CH), 125.64 (C), 129.14 (2CH), 129.49 (2CH), 129.83 (2CH), 131.21 (2CH), 133.08 (C), 136.11 (C), 146.79 (C)] 151.19 (C=N triazol), 151.71 (C=O), 152.38 (N=CH). LC Q-TOF *m*/*z*: [M+H]^+^ calcd for C_24_H_22_N_4_O_5_S, 479.13837; found, 479.14066.

(E)-4-(((3-(4-methylbenzyl)-5-oxo-1,5-dihydro-4H-1,2,4-triazol-4-yl)imino)methyl)phenyl 4-nitrobenzenesulfonate (**4g**): Yield 88.50%, m.p. 175–177 °C. IR (KBr, cm^−1^): 3181 (NH), 3053 (=CH), 1709 (C=O), 1592 (C=N), 1530 (C=C); ^1^H NMR (400 MHz, DMSO-d_6_) δ: 2.24 (s, CH_3_, 3H), 3.98 (s, CH_2_, 2H), benzene H [7.10 (d, 2H, J = 8 Hz), 7.17–7.23 (m, 4H), 7.84 (d, 2H, J = 8 Hz), 8.16 (d, 2H, J = 8 Hz), 8.45 (d, 2H, J = 8 Hz)], 9.68 (s, N=CH, 1H), 12.00 (s, NH, 1H); ^13^C NMR (100 MHz, DMSO-d_6_) δ: 20.74 (CH_3_), 30.74 (CH_2_), benzene C [123.29 (2CH), 125.59 (2CH), 129.11 (2CH), 129.49 (2CH), 130.04 (2CH), 130.60 (2CH), 133.07 (C), 133.56 (C), 136.27 (C), 139.67 (C), 146.82 (C), 150.91 (C)] 151.56 (C=N triazol), 151.62 (C=O), 152.10 (N=CH). LC Q-TOF *m*/*z*: [M+H]^+^ calcd for C_23_H_19_N_5_O_6_S, 494.11288; found, 494.11369.

(E)-4-(((3-(4-chlorobenzyl)-5-oxo-1,5-dihydro-4H-1,2,4-triazol-4-yl)imino)methyl)phenyl benzenesulfonate (**5a**): Yield 91.55%, m.p. 140–142 °C. IR (KBr, cm^−1^): 3160 (NH), 3066 (=CH), 1708 (C=O), 1598 (C=N), 1491 (C=C); ^1^H NMR (400 MHz, DMSO-d_6_) δ: 4.05 (s, CH_2_, 2H), benzene H [7.15 (d, 2H, J = 8 Hz), 7.32–7.38 (m, 4H), 7.67–7.71 (m, 2H), 7.80–7.85 (m, 3H), 7.89–7.90 (m, 2H)], 9.67 (s, N=CH, 1H), 12.03 (s, NH, 1H); ^13^C NMR (100 MHz, DMSO-d_6_) δ: 30.77 (CH_2_), benzene C [123.22 (2CH), 128.75 (2CH), 128.86 (2CH), 129.89 (2CH), 131.23 (2CH), 131.88 (C), 133.17 (C), 134.49 (C), 135.18 (C), 135.73 (CH), 146.38 (C)] 151.22 (C=N triazol), 151.55 (C=O), 152.22 (N=CH). LC Q-TOF m/z: [M+H]^+^ calcd for C22H17ClN4O4S, 469.07318; found, 469.07519.

(E)-4-(((3-(4-chlorobenzyl)-5-oxo-1,5-dihydro-4H-1,2,4-triazol-4-yl)imino)methyl)phenyl 4-fluorobenzenesulfonate (**5b**): Yield 90.85%, m.p. 148–150 °C. IR (KBr, cm^−1^): 3181 (NH), 3061 (=CH), 1708 (C=O), 1591 (C=N), 1492 (C=C); ^1^H NMR (400 MHz, DMSO-d_6_) δ: 4.05 (s, CH_2_, 2H), benzene H [7.18 (bs, 2H), 7.36 (bs, 4H), 7.54 (bs, 2H), 7.84 (bs, 2H), 7.97 (bs, 2H)], 9.71 (s, N=CH, 1H), 12.03 (s, NH, 1H); ^13^C NMR (100 MHz, DMSO-d_6_) δ: 30.46 (CH_2_), benzene C [117.67, 117.90 (2CH), 123.23 (2CH), 128.66 (2CH), 129.95 (2CH), 130.72, 130.75 (C), 129.87 (2CH), 130.23 (2CH), 131.89 (C), 132.15, 132.25 (2CH), 133.27 (C), 135.18 (C), 146.37 (C), 164.89, 167.42 (C)] 151.15 (C=N triazol), 151.55 (C=O), 152.45 (N=CH).

(E)-4-(((3-(4-chlorobenzyl)-5-oxo-1,5-dihydro-4H-1,2,4-triazol-4-yl)imino)methyl)phenyl 4-chlorobenzenesulfonate (**5c**): Yield 88.85%, m.p. 163–165 °C. IR (KBr, cm^−1^): 3176 (NH), 3061 (=CH), 1708 (C=O), 1589 (C=N), 1491 (C=C); ^1^H NMR (400 MHz, DMSO-d_6_) δ: 4.05 (s, CH_2_, 2H), benzene H [7.18 (d, 2H, J = 8 Hz), 7.36 (s, 4H), 7.76–7.91 (m, 6H)], 9.68 (s, N=CH, 1H), 12.04 (s, NH, 1H); ^13^C NMR (100 MHz, DMSO-d_6_) δ: 30.46 (CH_2_), benzene C [123.13 (2CH), 128.86 (2CH), 129.98 (2CH), 130.59 (2CH), 130.73 (2CH), 131.23 (2CH), 131.90 (C), 133.26 (C), 133.34 (C), 135.17 (C), 140.77 (C), 146.37 (C)] 151.10 (C=N triazol), 151.55 (C=O), 152.25 (N=CH). LC Q-TOF *m*/*z*: [M+H]^+^ calcd for C22H16Cl2N4O4S, 503.03421; found, 503.03623.

(E)-4-(((3-(4-chlorobenzyl)-5-oxo-1,5-dihydro-4H-1,2,4-triazol-4-yl)imino)methyl)phenyl 4-bromobenzenesulfonate (**5d**): Yield 82.60%, m.p. 183–185 °C. IR (KBr, cm^−1^): 3178 (NH), 3063 (=CH), 1709 (C=O), 1665 (C=N), 1597 (C=C); ^1^H NMR (400 MHz, DMSO-d_6_) δ: 4.04 (s, CH_2_, 2H), benzene H [7.18–7.82 (m, 8H), 7.82 (bs, 4H)], 9.69 (s, N=CH, 1H), 12.05 (s, NH, 1H); ^13^C NMR (100 MHz, DMSO-d_6_) δ: 30.46 (CH_2_), benzene C [115.51 (2CH), 123.28 (2CH), 125.54 (C), 128.86 (2CH), 129.87 (2CH), 131.23 (2CH), 131.89 (C), 131.90 (C), 133.01 (C), 135.18 (C), 146.32 (C)] 151.39 (C=N triazol), 151.56 (C=O), 152.43 (N=CH).

(E)-4-(((3-(4-chlorobenzyl)-5-oxo-1,5-dihydro-4H-1,2,4-triazol-4-yl)imino)methyl)phenyl 4-iodobenzenesulfonate (**5e**): Yield 89.99%, m.p. 222–224 °C. IR (KBr, cm^−1^): 3162 (NH), 3036 (=CH), 1694 (C=O), 1599 (C=N), 1567 (C=C); ^1^H NMR (400 MHz, DMSO-d_6_) δ: 4.06 (s, CH_2_, 2H), benzene H [7.19 (bs, 2H), 7.36 (bs, 4H), 7.63 (bs, 2H), 7.84 (bs, 2H), 8.08 (bs, 2H)], 9.68 (s, N=CH, 1H), 12.04 (s, NH, 1H); ^13^C NMR (100 MHz, DMSO-d_6_) δ: 30.78 (CH_2_), benzene C [123.26 (2CH), 128.86 (2CH), 129.86 (2CH), 130.15 (2CH), 131.24 (2CH), 131.84 (C), 133.20 (C), 133.94 (C), 135.18 (C), 139.30 (2CH), 146.28 (C)] 151.12 (C=N triazol), 151.55 (C=O), 152.27 (N=CH). LC Q-TOF *m*/*z*: [M+H]^+^ calcd for C22H16ClIN4O4S, 594.96983; found, 594.97185.

(E)-4-(((3-(4-chlorobenzyl)-5-oxo-1,5-dihydro-4H-1,2,4-triazol-4-yl)imino)methyl)phenyl 4-methoxybenzenesulfonate (**5f**): Yield 92.15%, m.p. 174–176 °C. IR (KBr, cm^−1^): 3182 (NH), 3064 (=CH), 1708 (C=O), 1664 (C=N), 1597 (C=C); ^1^H NMR (400 MHz, DMSO-d_6_) δ: 3.87 (s, OCH_3_, 3H), 4.00 (s, CH_2_, 2H), benzene H [7.13–7.19 (m, 4H), 7.35–7.36 (m, 4H), 7.81 (bs, 4H)], 9.67 (s, N=CH, 1H), 12.03 (s, NH, 1H); ^13^C NMR (100 MHz, DMSO-d_6_) δ: 30.17 (CH_2_), 56.35 (OCH_3_), benzene C [115.51 (2CH), 123.28 (2CH), 125.63 (C), 128.86 (2CH), 129.87 (2CH), 131.23 (2CH), 131.77 (C), 133.02 (C), 135.18 (C), 146.28 (C), 164.82 (C)] 151.14 (C=N triazol), 151.44 (C=O), 152.80 (N=CH). LC Q-TOF *m*/*z*: [M+H]^+^ calcd for C23H19ClN4O5S, 499.08375; found, 499.08585.

(E)-4-(((3-(4-chlorobenzyl)-5-oxo-1,5-dihydro-4H-1,2,4-triazol-4-yl)imino)methyl)phenyl 4-nitrobenzenesulfonate (**5g**): Yield 88.20%, m.p. 212–214 °C. IR (KBr, cm^−1^): 3174 (NH), 3061 (=CH), 1708 (C=O), 1596 (C=N), 1529 (C=C); ^1^H NMR (400 MHz, DMSO-d_6_) δ: 4.05 (s, CH_2_, 2H), benzene H [7.22 (d, 2H, J = 8 Hz), 7.32–7.38 (m, 4H), 7.83 (d, 2H, J = 8 Hz), 8.18 (d, 2H, J = 8 Hz), 8.46 (d, 2H, J = 8 Hz)], 9.71 (s, N=CH, 1H), 12.09 (s, NH, 1H); ^13^C NMR (100 MHz, DMSO-d_6_) δ: 30.76 (CH_2_), benzene C [123.29 (2CH), 125.59 (2CH), 128.87 (2CH), 130.08 (2CH), 130.59 (2CH), 131.21 (2CH), 131.89 (C), 133.55 (C), 135.16 (C), 139.66 (C), 146.36 (C), 150.93 (C)] 151.54 (C=N triazol), 151.62 (C=O), 152.22 (N=CH). LC Q-TOF *m*/*z*: [M+H]^+^ calcd for C22H16ClN5O6S, 514.05826; found, 514.05956.

The structures of the synthesized sulfonate derivatives were characterized by FT-IR, ^1^H NMR, ^13^C NMR, and LC-Q-TOF-MS analyses. For compounds **5b** and **5d**, however, the available LC-Q-TOF-MS data were not used for structural confirmation, and their structural assignments were based primarily on the FT-IR, ^1^H NMR, and ^13^C NMR data. The spectral data of the synthesized compounds are provided in Figures S1–S61, and their molecular formulas are listed under “Formulas of Synthesized Compounds” in the Supplementary File. All compounds were obtained in good to excellent yields ranging from 80.80% to 92.15%, indicating the efficiency of the synthetic procedure. The melting points varied considerably depending on the substituents present on the sulfonate moiety, further supporting the structural differences within the series.

In the FT-IR spectra, the characteristic N–H stretching vibrations of the 1,2,4-triazol-5-one ring were observed in the range of 3157–3190 cm^−1^. The azomethine C=N stretching bands appeared at approximately 1584–1600 cm^−1^, while the characteristic carbonyl C=O absorption of the triazolone ring was observed at 1690–1709 cm^−1^. The aromatic C=C stretching vibrations were detected in the range of 1491–1510 cm^−1^. For the methoxy-substituted derivatives, an additional C–O–C absorption was observed, consistent with the presence of the methoxy group. These characteristic absorptions collectively support the formation of the expected triazolone-Schiff base-sulfonate framework.

The ^1^H NMR spectra provided further evidence for the proposed structures. The azomethine proton (N=CH) was consistently observed as a singlet at approximately 9.64–9.71 ppm, confirming the formation of the Schiff base linkage. The NH proton of the triazolone ring appeared as a characteristic downfield singlet at 12.00–12.36 ppm, which can be attributed to its involvement in hydrogen bonding and the electron-withdrawing environment of the heterocyclic system. For derivatives **3a**–**3g**, the p-tolyl methyl group appeared at approximately 2.37 ppm, whereas the corresponding methyl group in **4a**–**4g** was observed at approximately 2.24–2.25 ppm. The benzyl CH_2_ protons of the latter series appeared at approximately 3.93–4.07 ppm. The aromatic protons were observed in the expected region, and their chemical shifts varied according to the electronic nature of the substituent on the sulfonate phenyl ring.

The ^13^C NMR spectra were also consistent with the proposed structures. The triazolone carbonyl carbon was observed at approximately 151.4–151.8 ppm, while the triazole C=N carbon resonated at approximately 151.1–151.5 ppm. The azomethine carbon appeared around 152.1–155.3 ppm. The methyl carbons of the p-tolyl or p-methylbenzyl groups were detected around 20.7–21.7 ppm, and the benzyl CH_2_ carbons of compounds **4a**–**4g** and **5a**–**5g** appeared near 30–31 ppm. The remaining aromatic carbon resonances were in agreement with the proposed substitution patterns. Electron-donating methoxy and electron-withdrawing fluoro, chloro, bromo, iodo, and nitro substituents also produced the expected changes in the aromatic carbon chemical shifts.

The structures of the newly synthesized compounds were characterized using FT-IR, ^1^H NMR, and ^13^C NMR spectroscopy, together with LC-Q-TOF-MS measurements. The mass spectrometric results revealed protonated molecular ion peaks that were generally consistent with the expected molecular masses of the target compounds. For compounds **5b** and **5d**, however, the available mass spectrometric data were not used as primary evidence for structural elucidation. The structural assignments of these compounds were based primarily on their FT-IR, ^1^H NMR, and ^13^C NMR data. The combined spectroscopic data provided evidence for the successful preparation and structural identification of the synthesized sulfonate derivatives.

The synthesized compounds were designed as hybrid molecules combining a sulfonate-containing aromatic moiety with a 1,2,4-triazol-3-one (triazolone) scaffold through an azomethine (Schiff base) linkage. The 1,2,4-triazol-3-one moiety provides several heteroatom-containing sites that may contribute to hydrogen-bonding and other noncovalent interactions with biological targets, while the azomethine linkage may contribute to molecular recognition and conformational organization. The para-substituent on the benzene-sulfonate moiety was systematically varied to investigate its contribution to biological activity. The unsubstituted derivative was used as the reference, whereas OCH_3_, F, Cl, Br, I, and NO_2_ substituents were introduced to examine the effects of electronic character, steric demand, and polarizability. In particular, comparison of the halogen-containing derivatives allowed the influence of increasing halogen size and polarizability (F, Cl, Br, and I) to be assessed, whereas OCH_3_ and NO_2_ provided contrasting electron-donating and electron-withdrawing properties. The observed differences in AChE and GST inhibitory activities and HepG2 cytotoxicity suggest that modification of the para-substituent can modulate the biological behavior of the hybrid scaffold. However, the activity trends cannot be attributed solely to electronic effects, indicating that a combination of steric, hydrophobic, polarizability, and molecular-recognition factors may contribute to the observed SAR.

### 2.2. Acetylcholinesterase and Glutathione S-Transferase Inhibitory Activities

The inhibitory activities of the synthesized compounds against acetylcholinesterase (AChE) and glutathione S-transferase (GST) are presented in Table 1. Overall, the synthesized derivatives exhibited potent inhibitory activities toward both enzymes, with IC_50_ values in the low micromolar range, demonstrating the suitability of the designed scaffold as a promising multitarget pharmacophore.

For AChE inhibition, the synthesized compounds exhibited IC_50_ values ranging from 1.60 ± 0.198 to 3.00 ± 0.912 µM, whereas the reference inhibitor donepezil (DNP) showed an IC_50_ value of 0.202 ± 0.031 µM under the same experimental conditions. Among the synthesized compounds, **3e** exhibited the highest AChE inhibitory activity, with an IC_50_ value of 1.60 ± 0.198 µM. Other compounds, including **4e** (1.74 ± 0.765 µM), **3f** (1.92 ± 0.291 µM), **4g** (1.96 ± 0.134 µM), **5d** (1.96 ± 0.475 µM), **4d** (2.02 ± 0.271 µM), **5e** (2.02 ± 0.179 µM), and **5f** (2.14 ± 0.814 µM), also exhibited relatively strong inhibitory activity among the synthesized derivatives. Compound **4c** (2.24 ± 0.265 µM) and several other derivatives showed IC_50_ values within a relatively narrow range, indicating comparable inhibitory activities among a number of the synthesized compounds. Overall, although the synthesized compounds were less potent than donepezil under the present assay conditions, several derivatives exhibited appreciable AChE inhibitory activity. These findings indicate that structural modifications of the triazole derivatives can modulate their inhibitory activity toward AChE.

In the GST inhibition assay, the synthesized derivatives exhibited IC_50_ values ranging from 1.95 ± 0.492 to 3.61 ± 0.216 µM, while the standard inhibitor ethacrynic acid (INN) showed an IC_50_ value of 3.066 ± 0.475 µM. Among all tested compounds, **4a** (1.95 ± 0.492 µM) showed the strongest GST inhibitory activity, corresponding to approximately 1.6-fold greater potency than ethacrynic acid. Likewise, **5c** (1.98 ± 0.420 µM), **3g** (2.02 ± 0.229 µM), **4b** (2.31 ± 1.068 µM), **3f** (2.32 ± 0.810 µM), **3b** (2.36 ± 0.341 µM), **4e** (2.39 ± 0.258 µM), **5e** (2.51 ± 0.716 µM), **5d** (2.71 ± 0.379 µM), **3e** (2.78 ± 0.210 µM), **5g** (2.81 ± 0.480 µM), **4g** (2.96 ± 0.357 µM), **3d** (3.00 ± 1.025 µM) and **4c** (3.00 ± 0.680 µM) all exhibited lower IC_50_ values than ethacrynic acid, whereas compounds **5a**, **5b**, **3c**, and **4f** showed comparable or slightly weaker inhibitory activities. The GST inhibitory activity observed for several derivatives identifies this enzyme family as a potentially relevant biochemical target of the synthesized compounds. However, because the present study did not include isoenzyme-specific structural analyses or cellular mechanistic experiments, these findings should be interpreted as evidence of in vitro GST inhibition rather than as demonstration of a specific GST-mediated anticancer mechanism.

A particularly noteworthy finding is that several derivatives displayed potent inhibitory activity against both target enzymes. Compound **3f** demonstrated the most balanced dual inhibitory profile, with IC_50_ values of 1.92 ± 0.291 µM for AChE and 2.32 ± 0.810 µM for GST, both of which were lower than the corresponding reference inhibitor values under the experimental conditions employed in this study. These findings indicate differences in the observed IC_50_ values among the synthesized compounds and the reference inhibitors, rather than demonstrating statistically superior inhibitory potency. Among the synthesized compounds, **3e** exhibited the strongest AChE inhibitory activity while also maintaining substantial GST inhibitory activity. Likewise, **4e** and **5d** showed relatively strong inhibitory activities toward both enzymes. These results suggest that several of the synthesized derivatives possess a favorable dual-inhibitory activity profile against AChE and GST, although their inhibitory potencies should be interpreted descriptively rather than as statistically superior to those of the reference inhibitors. Such dual inhibition is particularly attractive for the development of multitarget-directed ligands (MTDLs), since modulation of cholinergic dysfunction together with oxidative stress-related pathways has attracted considerable attention in the treatment of complex disorders, including neurodegenerative diseases and cancer.

The concentration-response curves yielded excellent linearity, with R^2^ values ranging from 0.9531 to 0.9995, confirming the robustness of the inhibition assays and the reliability of the calculated IC_50_ values. Collectively, these findings demonstrate that several of the newly synthesized derivatives possess inhibitory activities that are comparable to or superior to those of the standard inhibitors donepezil and ethacrynic acid. Among them, compound **3e** emerged as the most potent AChE inhibitor, compound **4a** showed the highest GST inhibitory activity, and compound 3f exhibited the most promising balanced dual inhibitory profile, making these derivatives attractive candidates for further biological and pharmacological investigations. The findings obtained in the present study are in good agreement with those reported in recent studies on triazole-based enzyme inhibitors. The synthesized compounds exhibited inhibitory activities within the low micromolar range, comparable to those described in the contemporary literature. Moreover, several derivatives demonstrated inhibitory potencies that were comparable to, or even exceeded, those of the standard inhibitors, donepezil for AChE and ethacrynic acid for GST. These results indicate that the structural modifications introduced into the synthesized scaffold successfully preserved or enhanced enzyme-binding affinity, highlighting the potential of these derivatives as promising candidates for the development of multifunctional therapeutic agents targeting both neurodegenerative disorders and cancer-related pathways [48,49,50].

### 2.3. Evaluation of Cytotoxic Effects

**Figure 1:**
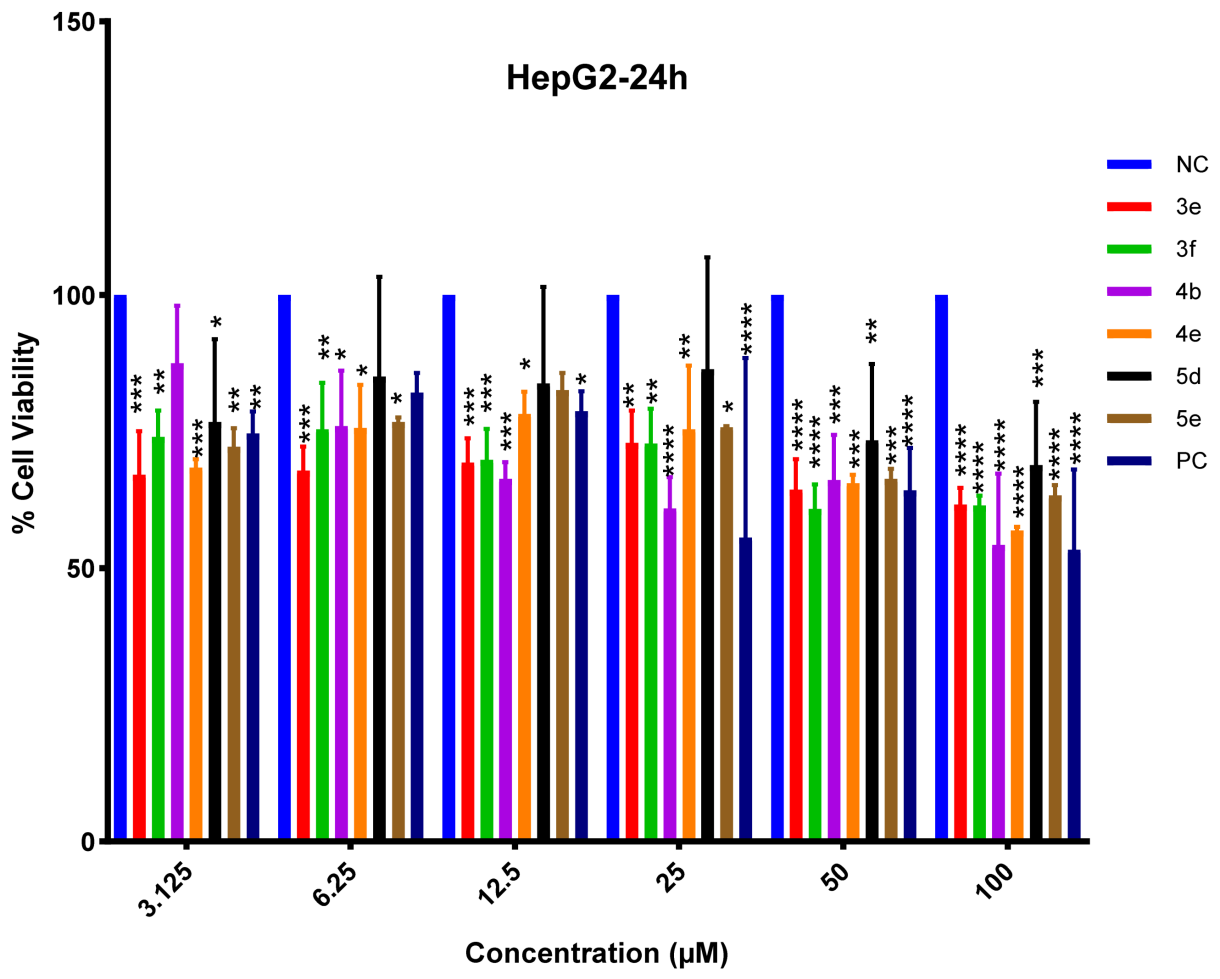
Effects of compounds **3e**, **3f**, **4b**, **4e**, **5d**, **5e**, and the positive control (PC) on the viability of HepG2 cells after 24 h of incubation, as determined by the MTT assay. Cell viability is expressed as the percentage relative to the negative control (NC). 5-Fluorouracil (5-FU) was used as the positive control. Data are presented as mean ± SD and were analyzed using two-way ANOVA. Statistical significance was defined as **** *p* ≤ 0.0001, *** *p* ≤ 0.0005, ** *p* ≤ 0.01, and * *p* ≤ 0.05 versus the negative control.

Before evaluating the cytotoxic effects of the synthesized compounds in HepG2 cells, their inhibitory activities against acetylcholinesterase (AChE) and glutathione S-transferase (GST) were comprehensively assessed. Based on the enzyme inhibition results, only the derivatives exhibiting the highest inhibitory potency were selected for subsequent cytotoxicity evaluation to determine whether their observed biochemical activities were accompanied by effects on HepG2 cell viability. This stepwise screening strategy enabled the biological characterization to focus on the most active candidates while reducing unnecessary testing of less active derivatives. Accordingly, compounds **3e**, **3f**, **4b**, **4e**, **5d**, and **5e**, which demonstrated the strongest enzyme inhibitory activities, were further evaluated for their in vitro cytotoxic effects against the human hepatocellular carcinoma (HepG2) cell line using the MTT assay. Cell viability was determined following 24 h and 48 h incubation periods to investigate the time-dependent effects of the selected compounds on HepG2 cell viability. The two incubation intervals allowed assessment of both the initial cytotoxic response and the sustained cytotoxic effects of the tested derivatives. 5-Fluorouracil (5-FU), a clinically established chemotherapeutic agent widely used in liver cancer studies, was employed as the positive control to provide a reference for comparison of the cytotoxic effects observed in HepG2 cells. The cytotoxic effects observed after 24 h of incubation are presented in Figure 1, illustrating the effects of the selected compounds on HepG2 cell viability.

**Figure 2:**
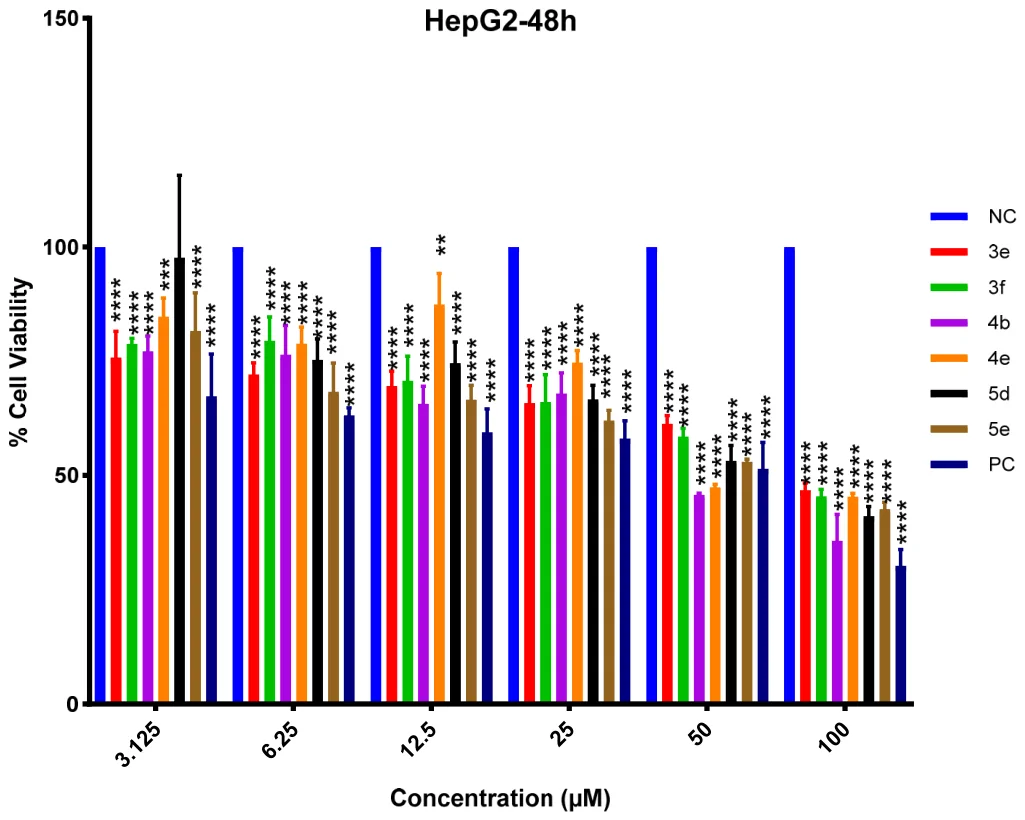
Effects of compounds **3e**, **3f**, **4b**, **4e**, **5d**, **5e**, and the positive control (PC) on the viability of HepG2 cells after 48 h of incubation, as determined by the MTT assay. Cell viability is expressed as the percentage relative to the negative control (NC). 5-Fluorouracil (5-FU) was used as the positive control. Data are presented as mean ± SD and were analyzed using two-way ANOVA. Statistical significance was defined as **** *p* ≤ 0.0001, *** *p* ≤ 0.0005, and ** *p* ≤ 0.01 versus the negative control.

The results obtained after 24 h of incubation demonstrated that compound **4b** exhibited the most pronounced cytotoxic effect among the tested derivatives, reducing HepG2 cell viability to a level comparable to that of the positive control. In contrast, compounds **3e** and **3f** also showed considerable cytotoxic activity, although their effects were less pronounced than those of compound **4b**. These findings indicate that compound **4b** possesses a strong and rapid antiproliferative effect against HepG2 cells within the first 24 h of exposure, whereas compounds **3e** and **3f** also display promising cytotoxic potential. The cytotoxic effects of the selected compounds following 48 h of incubation are presented in Figure 2, illustrating their time-dependent effects on HepG2 cell viability.

Following 48 h of incubation, compound **4b** consistently exhibited the strongest cytotoxic activity against HepG2 cells, maintaining an antiproliferative effect comparable to that of the positive control. In addition to compounds **3e** and **3f**, compound **5e** also demonstrated a significant reduction in cell viability after prolonged exposure, indicating a time-dependent enhancement of its cytotoxic activity. The increased activity of compound **5e** at 48 h suggests that extended incubation may potentiate its antiproliferative effect on HepG2 cells. Overall, the cytotoxicity results demonstrated that all selected compounds significantly reduced the viability of HepG2 cells compared with the negative control. Moreover, several derivatives exhibited cytotoxic activities approaching that of the positive control (5-fluorouracil), highlighting the promising anticancer potential of these synthesized compounds. Interestingly, compound **4b** exhibited pronounced cytotoxic activity despite its relatively modest inhibitory activity against the investigated enzymes. This finding suggests that enzyme inhibitory potency and cytotoxicity may not necessarily show a direct relationship. Therefore, the relationship between these activities should be considered only as a hypothesis, and the cytotoxic effect of **4b** may involve additional cellular mechanisms that were not investigated in the present study. These findings, together with their potent AChE and GST inhibitory activities, suggest that the selected derivatives represent.

### 2.4. Molecular Docking and Binding Mode Analysis

**Figure 3:**
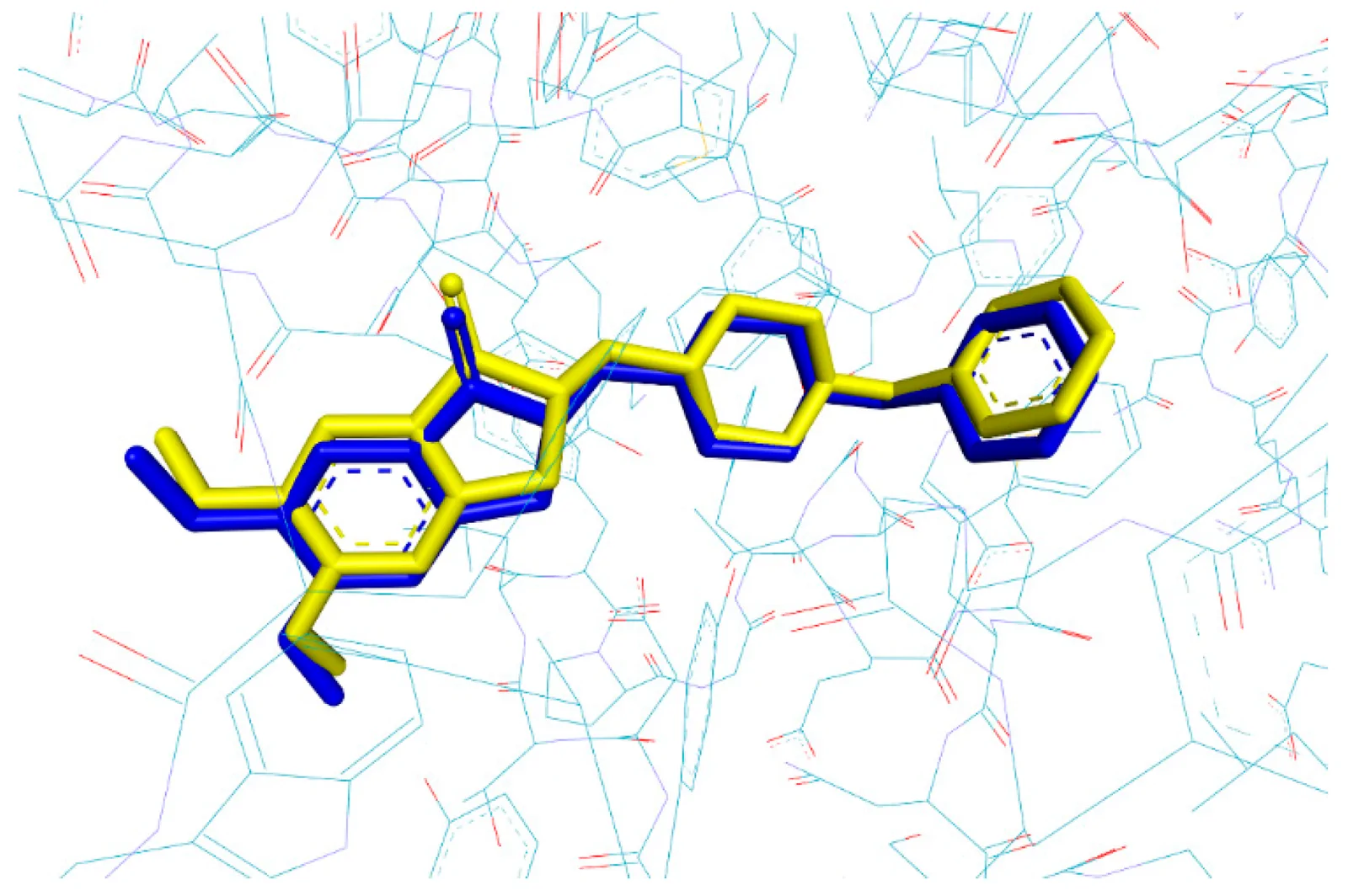
Validation of the docking protocol by re-docking donepezil into the hAChE binding site. The crystallographic pose of donepezil is shown in blue, whereas the redocked pose is shown in yellow. Superposition of the two conformations yielded an RMSD of 0.54 Å.

Molecular docking analysis was performed to provide a structural interpretation of the experimentally observed hAChE inhibitory activity of the synthesized derivatives. The reliability of the docking protocol was initially evaluated by re-docking the co-crystallized inhibitor donepezil into the ligand-binding gorge of hAChE (PDB ID: 4EY7). This structure corresponds to a 2.35 Å resolution X-ray crystal structure of recombinant human AChE in complex with donepezil and therefore provides an experimentally validated framework for structure-based analysis of AChE inhibitors [51]. Superposition of the redocked and crystallographic conformations of donepezil yielded an RMSD of 0.54 Å (Figure 3), indicating close reproduction of the experimentally observed binding orientation.

Docking of the complete series showed that all 21 synthesized derivatives could be accommodated favorably within the hAChE binding gorge, with predicted binding energies ranging from −13.10 to −14.67 kcal/mol (Supplementary Table S1). Among the investigated compounds, **3e** exhibited the most favorable binding energy (−14.67 kcal/mol**)**, followed by **3d** (−14.30 kcal/mol), **5e** (−14.24 kcal/mol), **5d** (−14.18 kcal/mol), and **3c** (−14.17 kcal/mol) (Table 2)**.** Importantly, **3e** was also the most potent inhibitor in the experimental AChE assay, with an IC_50_ value of 1.60 ± 0.198 µM**,** indicating qualitative consistency between its favorable docking profile and experimentally observed inhibitory activity. Compound **3f**, which also displayed strong AChE inhibition experimentally (IC_50_ = 1.92 ± 0.291 µM), exhibited a calculated binding energy of −13.62 kcal/mol. Donepezil, used as the reference inhibitor, showed a docking energy of −11.27 kcal/mol. These calculated docking energies were considered primarily as comparative indicators of ligand–receptor complementarity and were not interpreted as direct quantitative equivalents of the experimentally determined IC_50_ values.

**Figure 4:**
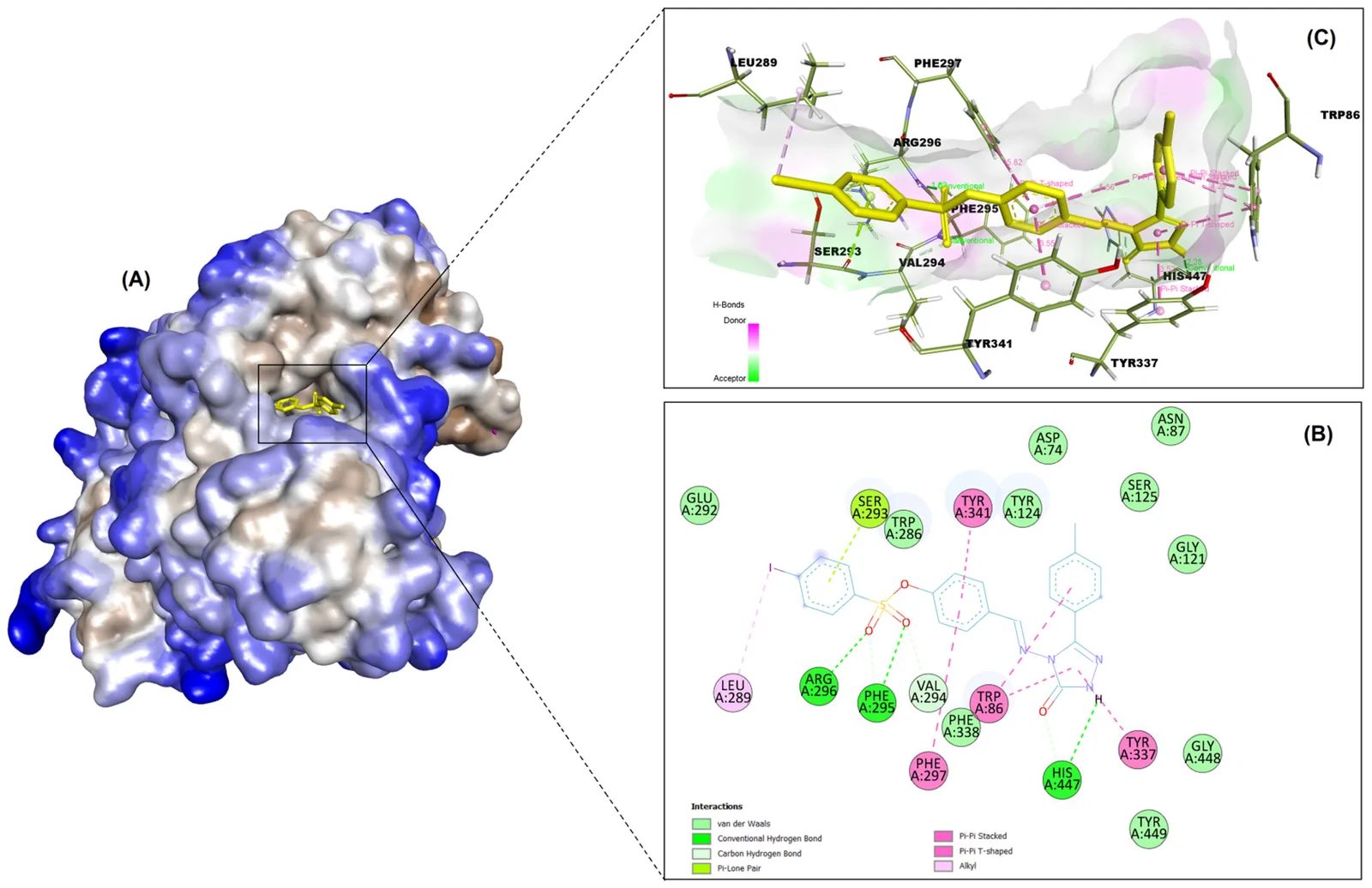
Binding mode of compound **3e** within the active-site gorge of human acetylcholinesterase (hAChE). (**A**) Surface representation of the docked complex, (**B**) 2D interaction map, and (**C**) 3D view of key ligand–residue interactions.

Detailed analysis of the binding mode of **3e** revealed an extended orientation within the AChE gorge involving Trp86, Leu289, Ser293, Val294, Phe295, Arg296, Phe297, Tyr337, Tyr341, and His447 (Figure 4). Conventional hydrogen bonds were identified with Phe295, Arg296, and His447**,** together with additional carbon–hydrogen, hydrophobic, and π-mediated contacts. The involvement of these residues is structurally relevant because human AChE contains several functionally distinct subsites distributed along its deep active-site gorge. Trp86 and Tyr337 form major components of the choline-binding region, whereas Phe295 and Phe297 constitute the acyl-binding pocket. Tyr341**,** together with Tyr72, Asp74, Tyr124, and Trp286, is associated with the peripheral region near the entrance of the gorge, while His447 is a member of the catalytic triad [52,53,54]. Thus, the interaction pattern predicted for **3e** suggests that the molecule occupies an extended portion of the AChE gorge and establishes contacts with residues involved in substrate recognition (Trp86 and Tyr337), acyl-pocket organization (Phe295 and Phe297), the peripheral region (Tyr341), and catalytic-site architecture (His447) [52,53,54].

**Figure 5:**
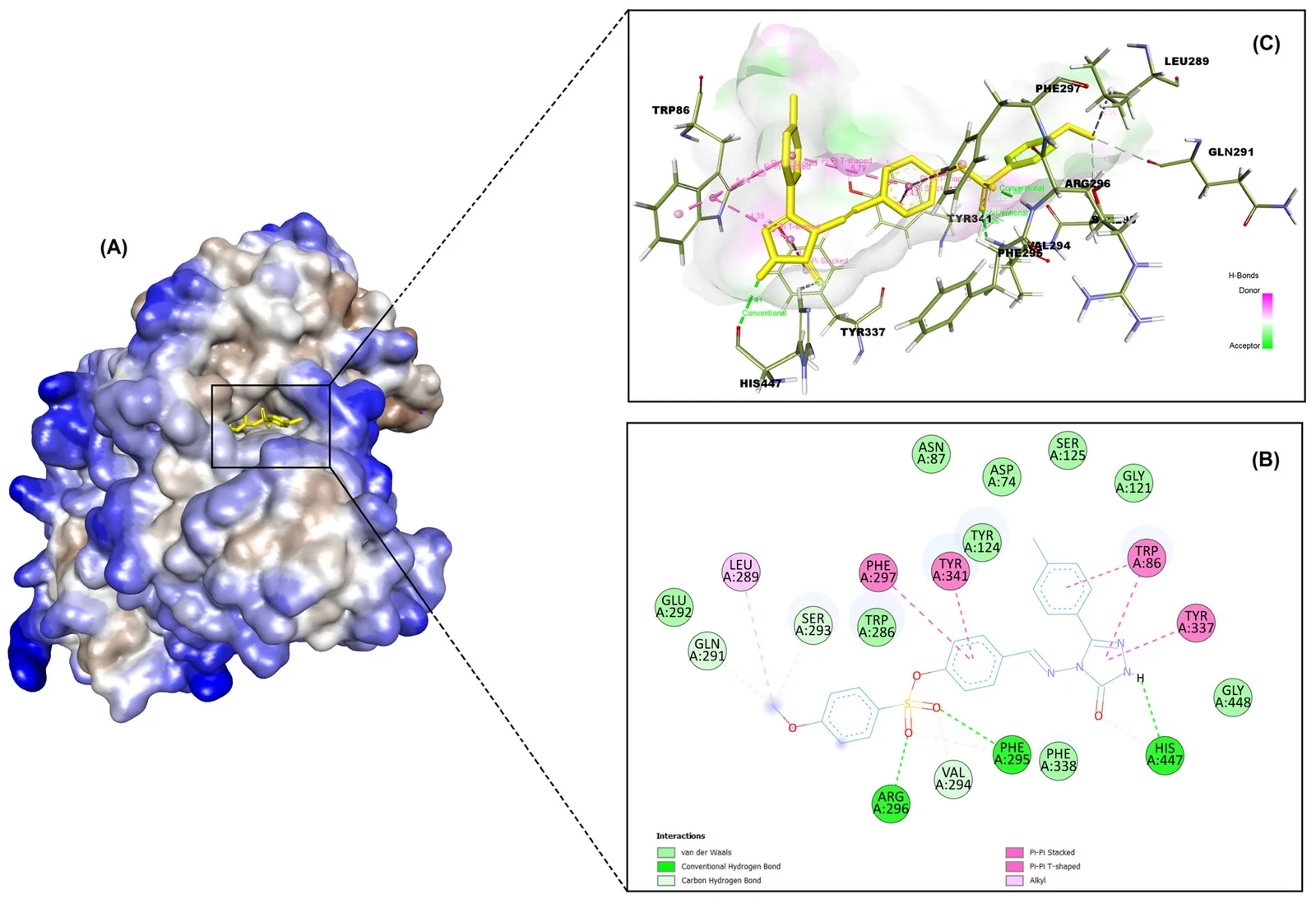
Binding mode of compound **3f** within the active-site gorge of human acetylcholinesterase (hAChE). (**A**) Surface representation of the docked complex, (**B**) 2D interaction map, and (**C**) 3D view of key ligand–residue interactions.

The docking analysis of compound **3f** revealed a binding mode comparable to that of **3e**, with predicted contacts involving Trp86, Leu289, Gln291, Ser293, Val294, Phe295, Arg296, Phe297, Tyr337, Tyr341, and His447 (Figure 5). Hydrogen-bond interactions were predicted with Phe295, Arg296, and His447, while additional aromatic and hydrophobic interactions contributed to the binding profile. Comparison of the docking results for **3e** and **3f** revealed overlapping interactions with key residues, particularly Trp86, Phe295, Phe297, Tyr337, Tyr341, and His447, suggesting that both active compounds may adopt similar interaction patterns within the AChE binding gorge. Previous structural and computational studies have similarly emphasized the importance of the aromatic environment of the AChE active-site gorge, particularly Trp86, Tyr337, Phe295, Phe297, Trp286, and Tyr341, in the recognition and stabilization of structurally diverse inhibitors [52,53,55]. The importance of these regions has also been observed in docking studies of chemically distinct AChE inhibitors, where occupation of both the deeper choline/acyl-binding regions and the peripheral portion of the gorge contributes to favorable ligand accommodation [56].

The donepezil complex provided a useful structural benchmark for interpreting the binding modes of the synthesized derivatives. In the present redocking analysis, donepezil interacted with Tyr72, Trp86, Trp286, Ser293, Phe295, Phe338, Tyr337, Tyr341, and His447. This interaction profile is consistent with the experimentally determined 4EY7 structure and previous structure-based analyses of donepezil binding. In particular, donepezil is known to extend along the AChE gorge, with aromatic interactions involving Trp86 in the deeper choline-binding region and Trp286 toward the peripheral site, while Phe295, Tyr337, Phe338, and Tyr341 further contribute to stabilization of the ligand within the gorge [51,56]. This characteristic distribution of interactions across spatially distinct regions of AChE explains why donepezil is frequently used as a structural reference when evaluating extended or gorge-spanning AChE inhibitors.

Overall, the docking findings provide a plausible structural basis for the pronounced experimental AChE inhibition observed for **3e** and **3f**. In particular, their ability to establish simultaneous hydrogen-bonding, hydrophobic, and aromatic contacts with several residues lining the catalytic gorge may contribute to favorable molecular recognition. Nevertheless, docking represents a static approximation of ligand binding and does not establish the persistence of these interactions under dynamic conditions. Compounds **3e** and **3f** were selected for MD simulations based on their strong AChE inhibitory activities, with **3e** being the most potent AChE inhibitor and **3f** exhibiting a strong and balanced AChE/GST inhibitory profile. Since the MD analysis was specifically designed to investigate AChE binding behavior, compound selection was based primarily on enzyme inhibition rather than cytotoxic activity. Therefore, the hAChE–**3e**, hAChE–**3f**, and hAChE–donepezil complexes were subsequently subjected to 100 ns molecular dynamics simulations to evaluate their conformational stability and time-dependent interaction behavior.

### 2.5. Dynamic Stability and Structural Behavior of hAChE–Ligand Complexes

To further evaluate the stability of the binding modes predicted by molecular docking, 100 ns molecular dynamics simulations were performed for the hAChE–**3e**, hAChE–**3f**, and hAChE–donepezil complexes. Compounds **3e** and **3f** were selected based on their experimental AChE inhibitory profiles, with **3e** showing the strongest AChE inhibition and **3f** exhibiting strong AChE inhibition together with a balanced AChE/GST inhibitory profile. Donepezil was included as the reference AChE inhibitor. MD simulations provide a dynamic perspective on protein–ligand recognition and are particularly useful for assessing whether docking-derived poses remain structurally stable over time within the highly confined and conformationally responsive AChE binding gorge [53,57].

**Figure 6:**
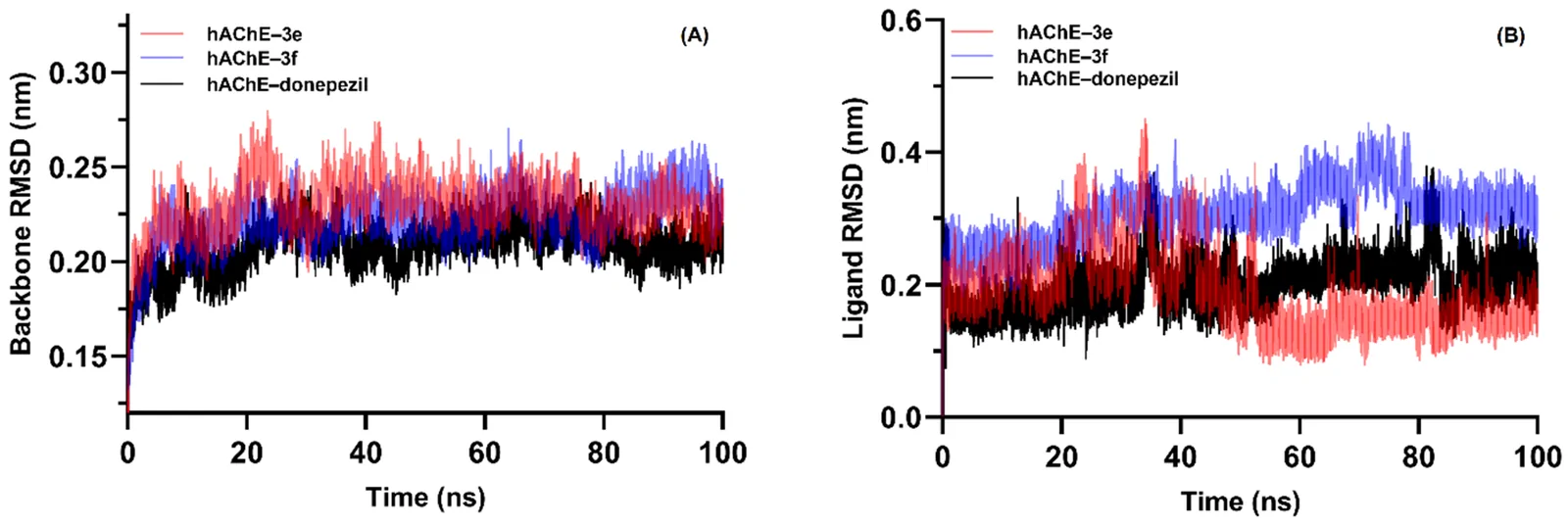
RMSD profiles of the hAChE–ligand complexes during 100 ns molecular dynamics simulations. (**A**) Backbone RMSD profiles of hAChE in complex with **3e**, **3f**, and donepezil. (**B**) Ligand RMSD profiles of **3e**, **3f,** and donepezil calculated after fitting the trajectories to the protein backbone.

The backbone RMSD profiles showed that all three complexes underwent an initial adjustment during the early stage of the simulations and subsequently maintained relatively stable conformations throughout the remaining trajectory (Figure 6A). Inspection of the trajectories indicated comparatively stable behavior during the latter half of the simulations; therefore, quantitative RMSD comparisons were performed over the 50–100 ns interval. Over this interval, the mean backbone RMSD values were 0.174 ± 0.012 nm for hAChE–**3e**, 0.168 ± 0.010 nm for hAChE–**3f**, and 0.164 ± 0.011 nm for hAChE–donepezil. Although the donepezil complex exhibited slightly lower deviations, the differences among the systems were modest and no progressive increase indicative of global structural destabilization was observed. Comparable low-amplitude backbone fluctuations have been reported in previous MD studies of AChE inhibitor complexes and are generally associated with preservation of the overall protein fold during simulation [58].

Ligand RMSD analysis provided additional insight into the positional stability of the compounds within the AChE gorge (Figure 6B). Compound **3e** showed moderate rearrangement during the first half of the trajectory, followed by a pronounced reduction in RMSD after approximately 50 ns, indicating adoption of a more confined binding orientation during the latter part of the simulation. Its mean ligand RMSD over 50–100 ns was 0.146 ± 0.028 nm. In contrast, 3f exhibited higher positional fluctuations, with a mean RMSD of 0.333 ± 0.031 nm, particularly between approximately 55 and 80 ns. Nevertheless, the absence of a continuous increase or irreversible displacement suggests that **3f** remained associated with the binding cavity while sampling a broader conformational space. Donepezil displayed an intermediate and comparatively consistent profile, with a mean ligand RMSD of 0.212 ± 0.033 nm. These observations indicate that **3e** remained closer to its reference binding orientation during the latter part of the simulation, whereas **3f** sampled a broader conformational space within the binding gorge. These RMSD differences were interpreted together with the complementary structural and interaction analyses rather than as direct measures of binding stability.

**Figure 7:**
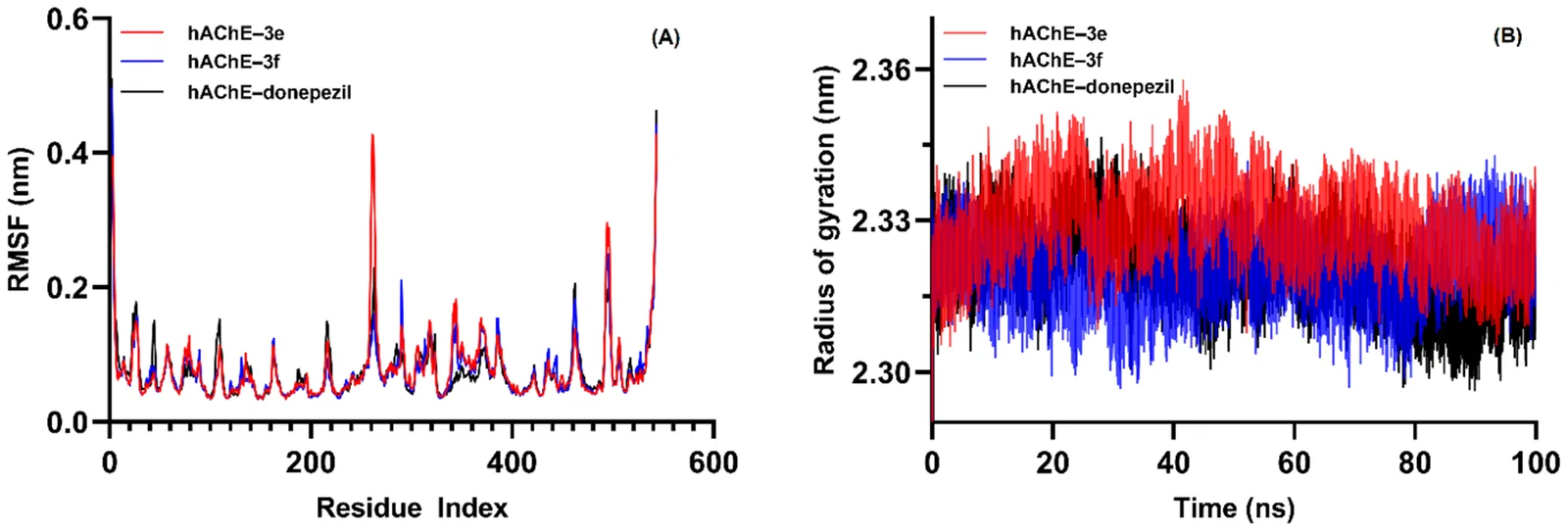
Structural flexibility and compactness of hAChE–ligand complexes during 100 ns MD simulations. (**A**) Residue-wise RMSF profiles of hAChE in complex with **3e**, **3f**, and donepezil. (**B**) Radius of gyration (Rg) profiles of the corresponding complexes.

The residue-wise RMSF profiles were highly similar among the three ligand-bound systems (Figure 7A), indicating no major differences in the overall flexibility pattern of hAChE among the investigated complexes. Most residues displayed relatively low fluctuations, whereas the largest peaks were confined primarily to terminal and flexible loop regions. Such localized flexibility is consistent with previous structural and simulation studies showing that cholinesterases retain a comparatively rigid catalytic core while allowing greater mobility in peripheral and gorge-entrance regions [53]. The overall similarity of the RMSF profiles therefore supports the conclusion that neither 3e nor 3f caused substantial perturbation of the receptor structure relative to donepezil.

Consistent with the RMSD and RMSF findings, the radius of gyration profiles remained stable throughout the simulations (Figure 7B). Mean Rg values over 50–100 ns were 2.327 ± 0.006 nm for hAChE–**3e**, 2.321 ± 0.006 nm for hAChE–**3f**, and 2.317 ± 0.006 nm for hAChE–donepezil. The narrow distribution and absence of sustained drift indicate preservation of the global compactness of hAChE in all three ligand-bound systems. Similar Rg behavior has been reported for other AChE–inhibitor complexes, where maintenance of a stable gyration radius is interpreted as evidence against ligand-induced unfolding or major domain rearrangement [57,58].

The SASA profiles likewise showed no persistent directional change over the 100 ns trajectories (Figure 6A), suggesting that the overall solvent-exposed surface of hAChE remained largely preserved. The **3e** and **3f** complexes displayed modestly higher solvent exposure than the donepezil system during portions of the simulations, while **3f** exhibited a transient increase near the end of the trajectory. However, these variations were limited and were not accompanied by corresponding evidence of global destabilization in either the RMSD or Rg analyses. Thus, the SASA behavior is more consistent with local conformational breathing and solvent-accessibility changes than with major structural reorganization.

**Figure 8:**
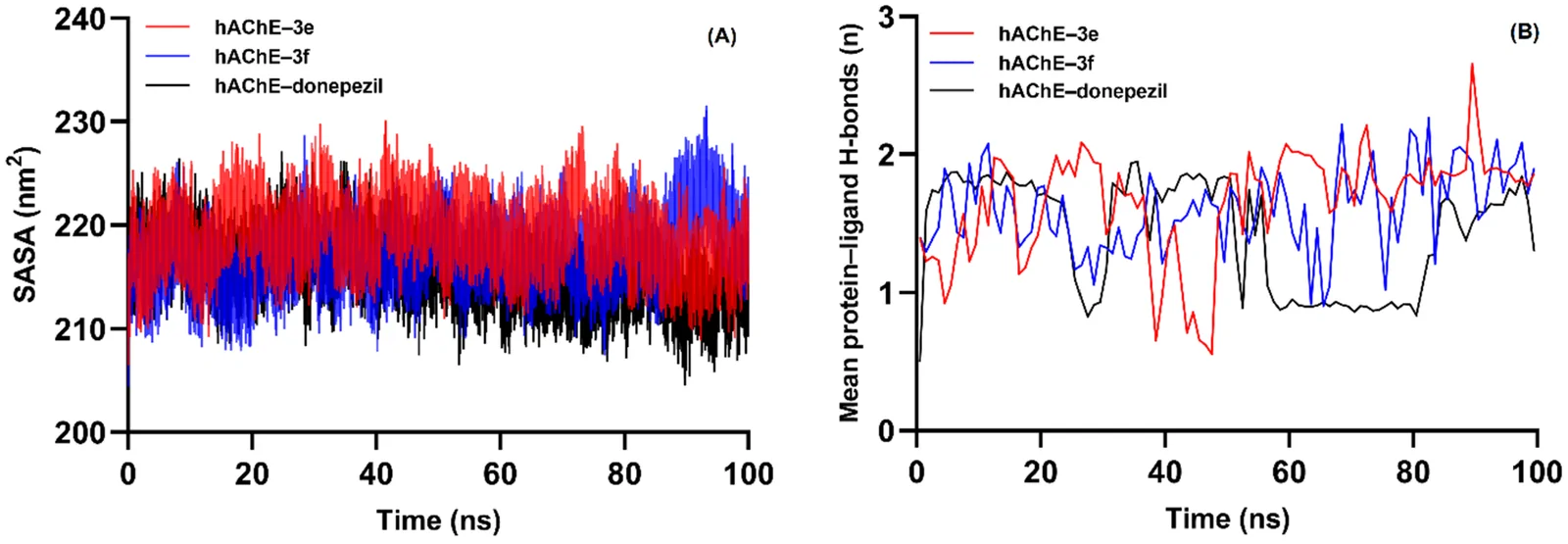
Solvent exposure and intermolecular hydrogen-bonding behavior of hAChE–ligand complexes during 100 ns MD simulations. (**A**) SASA profiles of the hAChE–**3e**, hAChE–**3f**, and hAChE–donepezil complexes. (**B**) Mean protein–ligand hydrogen-bond profiles calculated using 1 ns time-binned averages.

Protein–ligand hydrogen bonding was maintained throughout the simulations in all three systems (Figure 8B). Consistent with the revised RMSD and Rg analyses, quantitative hydrogen-bond analysis was performed over the comparatively stable 50–100 ns portion of the trajectories, yielding mean hydrogen-bond numbers of 1.86 ± 0.59 for **3e**, 1.70 ± 0.86 for **3f**, and 1.22 ± 0.59 for donepezil. Although individual hydrogen bonds formed and dissociated dynamically, both synthesized derivatives maintained on average approximately one to two intermolecular hydrogen bonds during most of the simulation period. Compound **3e** exhibited the highest mean number of protein–ligand hydrogen bonds among the three systems, which is consistent with its extensive hydrogen-bonding network observed in the docking analysis. Previous AChE studies combining docking and molecular dynamics have similarly shown that persistent, dynamically exchanging interactions can contribute to stabilization of inhibitors within the gorge even when individual contacts are not continuously maintained throughout the trajectory [57,58,59].

Taken together, the MD results support the structural plausibility of the docking-derived complexes and provide a dynamic context for the experimentally observed AChE inhibition. All three systems preserved stable protein conformations and overall compactness during the 100 ns simulations. Among the synthesized derivatives, **3e** combined a comparatively low ligand RMSD during the 50–100 ns interval with maintained hydrogen-bonding interactions and stable global protein behavior, indicating that it remained relatively close to its reference binding orientation during the latter part of the trajectory. Compound **3f**, although more mobile within the binding gorge, remained associated with the receptor and maintained a stable protein-level trajectory, suggesting a more flexible but still persistent binding mode. These dynamic differences were subsequently examined at the energetic level using MM/GBSA binding free energy calculations and per-residue energy decomposition.

Each complex was represented by a single 100 ns trajectory. Accordingly, the reported means and standard deviations for trajectory-derived metrics and MM/GBSA snapshots describe variation within the sampled trajectory only; they do not quantify variability across independent simulations or establish statistical reproducibility. The comparisons among systems should therefore be interpreted as descriptive.

### 2.6. Binding Free Energy and Per-Residue Decomposition

To complement the docking and MD analyses with an energetic assessment of ligand recognition, MM/GBSA calculations were performed using 200 snapshots extracted from the final 20 ns (80–100 ns) of the trajectories, representing the terminal portion of the simulations selected for energetic analysis. MM/GBSA provides an end-state estimate of binding energetics by combining molecular-mechanics and implicit-solvent contributions and is widely used for comparative analysis of protein–ligand complexes generated from MD simulations [60].

All three complexes exhibited favorable calculated binding energies (Table 3). The reference hAChE–donepezil complex showed the most favorable total MM/GBSA energy, with a Δ*G*_bind_ of −53.35 ± 2.99 kcal/mol, followed by hAChE–**3e** (−35.15 ± 3.24 kcal/mol) and hAChE–**3f** (−25.34 ± 3.18 kcal/mol). For both synthesized compounds, van der Waals interactions constituted a major favorable contribution, amounting to −54.01 ± 3.02 kcal/mol for **3e** and −55.63 ± 2.14 kcal/mol for 3f. However, **3e** displayed a more favorable electrostatic contribution than 3f (−18.36 ± 3.47 vs. −6.62 ± 4.85 kcal/mol), which partly accounts for its more favorable overall binding-energy profile.

Donepezil displayed a distinct energetic signature (Table 3). Its highly favorable electrostatic term (−233.25 ± 7.09 kcal/mol) was largely offset by an unfavorable polar solvation contribution (+246.61 ± 7.03 kcal/mol), while van der Waals interactions remained strongly favorable (−60.29 ± 2.55 kcal/mol). The resulting total binding energy nevertheless remained the most favorable among the three systems. Such compensation between direct electrostatic interactions and polar desolvation is characteristic of MM/GBSA calculations and highlights the importance of interpreting individual energy components together rather than in isolation [60]. Because configurational entropy was not included, the calculated values should be regarded as comparative energetic estimates rather than absolute experimental binding free energies.

**Figure 9:**
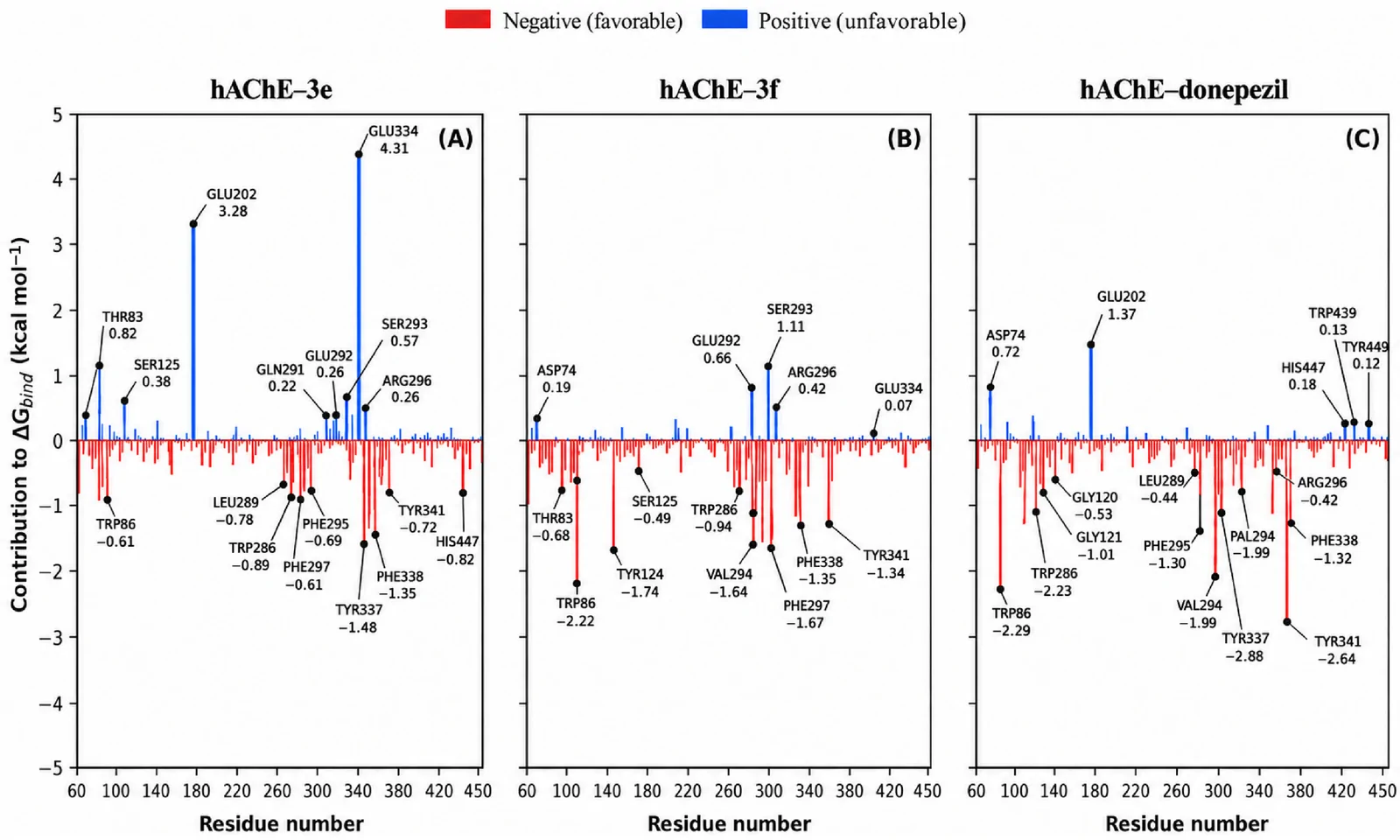
Residue-wise binding free energy decomposition of the hAChE–**3e** (**A**), hAChE–**3f** (**B**), and hAChE–donepezil (**C**) complexes. Negative values denote favorable contributions to ligand binding, whereas positive values indicate unfavorable contributions. Major contributing residues are labeled with their mean energy values.

Per-residue energy decomposition provided further insight into the amino acid contributions underlying ligand stabilization (Figure 7). For **3e**, the principal favorable contributions involved Tyr337, Phe338, Trp286, His447, Leu289, Tyr341, Phe295, Trp86, and Phe297 (Figure 9A), with Tyr337 showing the strongest favorable contribution among the analyzed residues. Notably, many of these residues had also been identified in the docking analysis, thereby linking the static binding mode with the energetic behavior observed during the MD-derived trajectory. Trp86, Tyr337, Trp286, and Tyr341 are recognized as major aromatic determinants of ligand recognition along the AChE gorge, whereas Phe295 and Phe297 contribute to the acyl-binding region [51,61].

For **3f**, the most favorable residue-level contributions were associated with Trp86, Tyr124, Phe297, Val294, Phe338, Tyr341, Phe295, and Trp286 (Figure 9B). The prominent contribution of Trp86 agrees with the docking-derived interaction pattern, in which this residue was positioned close to the ligand within the catalytic gorge. Although **3f** showed a less favorable overall MM/GBSA energy and greater ligand mobility than **3e**, its decomposition profile indicates that it remained stabilized by a broad network of aromatic and hydrophobic contacts. Previous structural and computational studies of AChE inhibitors have similarly emphasized the roles of Trp86, Trp286, Tyr337, and Tyr341 in anchoring ligands within the gorge [51,61].

The donepezil decomposition profile was dominated by favorable contributions from Tyr337, Tyr341, Trp86, Trp286, Val294, Phe338, and Phe295 (Figure 9C). This pattern closely reflects the experimentally established gorge-spanning binding mode of donepezil in human AChE and agrees with previous structural descriptions showing simultaneous involvement of the deeper catalytic/anionic region and the peripheral aromatic site [51]. The recurrence of several of the same residues in the **3e** and **3f** complexes suggests that the synthesized derivatives exploit interaction hotspots that are also important for the reference inhibitor, although the relative energetic contributions differ among the ligands.

Overall, the energetic analysis complemented the docking and MD findings by differentiating the relative energetic behavior of the selected complexes. Although the MM/GBSA ranking did not fully reproduce the experimental IC_50_ order, **3e** showed a more favorable binding-energy profile than **3f**, consistent with their relative experimental activities. The stronger calculated binding energy of donepezil further emphasizes that MM/GBSA values should be interpreted as comparative computational estimates rather than direct predictors of experimental inhibitory potency.

To provide a preliminary assessment of the physicochemical and pharmacokinetic properties of the selected compounds, **3e**, **3f**, and **4a** were further evaluated using SwissADME. Compounds **3f** and **4a** showed no Lipinski rule violations and predicted bioavailability scores of 0.55, whereas **3e** showed two Lipinski violations and a lower bioavailability score of 0.17. High GI absorption was predicted for **3e** and **4a**, while **3f** showed low predicted GI absorption. None of the three compounds was predicted to permeate the BBB or to act as a P-gp substrate. These computational predictions provide a preliminary indication of their pharmacokinetic profiles and require experimental validation. Detailed results are provided in Supplementary Table S2.

## 3. Materials and Methods

### 3.1. Instruments

The IR spectra of the synthesized compounds were recorded using a PerkinElmer FT-IR 1600 spectrophotometer (PerkinElmer, Waltham, MA, USA) in the range of 4000–400 cm^−1^. The 1H-NMR and 13C-NMR spectra were obtained using a Bruker 400 MHz NMR spectrometer (Bruker, Billerica, MA, USA), with DMSO-d_6_ as the solvent. Mass spectra were obtained using a Thermo Surveyor TSQ Quantum Access mass spectrometer (Thermo Fisher Scientific, San Jose, CA, USA). All solvents and reagents used in the synthesis and structural characterization were purchased from Fluka (Buchs, Switzerland), Merck (Darmstadt, Germany), and Aldrich (St. Louis, MO, USA), and were subjected to appropriate purification and drying procedures before use.

### 3.2. Biological Activity

#### 3.2.1. General Remarks

The cell line was purchased from Sigma-Aldrich (St. Louis, MO, USA). The cell line was originally obtained from the European Collection of Authenticated Cell Cultures (ECACC). The relevant catalog number is ECACC/Sigma-Aldrich, Catalog No. 89081402.

The evaluation conducted in the present study comprised cytotoxicity testing against the HepG2 human hepatocellular carcinoma cell line together with inhibitory activity assays targeting glutathione S-transferase (GST) and acetylcholinesterase (AChE). HepG2 cells were maintained in RPMI-1640 culture medium (Gibco, Grand Island, NY, USA) supplemented with 10% fetal bovine serum (FBS; Biological Industries, Beit HaEmek, Israel) and 1% penicillin–streptomycin (P/S; Sigma-Aldrich, St. Louis, MO, USA). Cell viability was assessed using the 3-(4,5-dimethylthiazol-2-yl)-2,5-diphenyltetrazolium bromide (MTT) assay (Sigma-Aldrich, St. Louis, MO, USA). Enzyme inhibition experiments employed cytosolic glutathione S-transferase from equine liver (GST; Sigma-Aldrich, St. Louis, MO, USA, G6511) and acetylcholinesterase from *Electrophorus electricus* (electric eel; Sigma-Aldrich, St. Louis, MO, USA, C3389). 1-Chloro-2,4-dinitrobenzene (CDNB; Sigma-Aldrich, St. Louis, MO, USA) and reduced glutathione (GSH; Sigma-Aldrich, St. Louis, MO, USA) were used for the GST assay, whereas acetylthiocholine iodide (ATCI; Sigma-Aldrich, St. Louis, MO, USA) and 5,5′-dithiobis-(2-nitrobenzoic acid) (DTNB; Sigma-Aldrich, St. Louis, MO, USA) were used for the AChE inhibition assay. Dimethyl sulfoxide (DMSO; Sigma-Aldrich, St. Louis, MO, USA) served as the solvent for the synthesized compounds, and phosphate-buffered saline (PBS; Gibco, Grand Island, NY, USA) was used where required during the experimental procedures. All enzyme inhibition experiments were performed using three independent experimental replicates, and the results were expressed as mean ± standard deviation. All reagents were of analytical grade and were handled in accordance with standard laboratory practices to ensure experimental reliability and reproducibility.

#### 3.2.2. Spectrophotometric Determination of GST Activity

**Figure 10:**
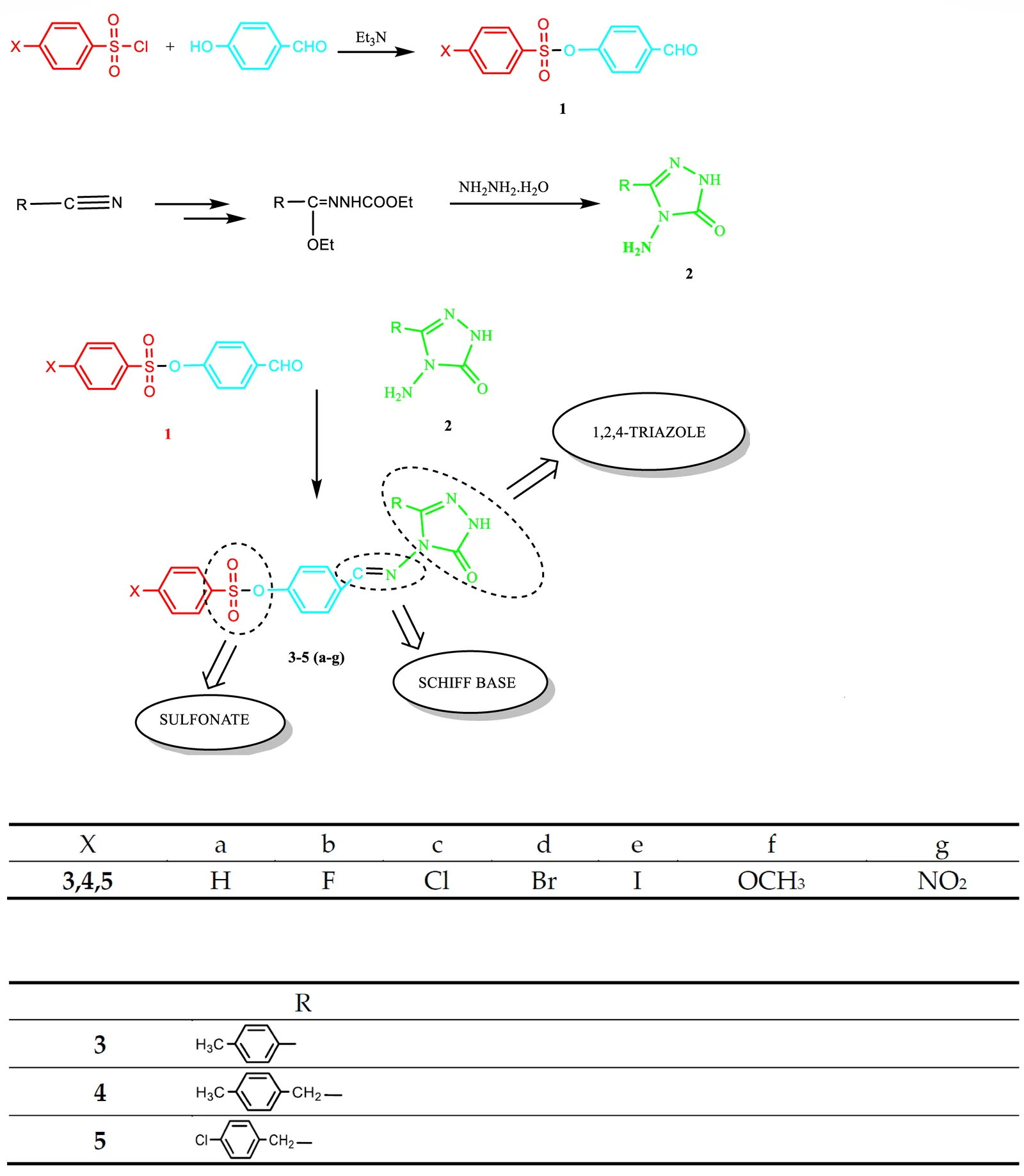
IC_50_ Values of the Synthesized Compounds Against AChE and GST Enzymes.

Glutathione S-transferase (GST) inhibitory activity was evaluated spectrophotometrically according to the method described by Habig and Jakoby [62], based on the conjugation of 20 mM reduced glutathione (GSH) with 25 mM 1-chloro-2,4-dinitrobenzene (CDNB). The enzymatic reaction was monitored by measuring the increase in absorbance of the GS-DNB conjugate at 340 nm. The reaction mixture consisted of 0.2 M sodium phosphate buffer (pH 6.5), reduced glutathione (GSH), 1-chloro-2,4-dinitrobenzene (CDNB), the GST enzyme solution, and the test compound at the appropriate concentration. A corresponding control containing all reaction components except the test compound was included to determine the basal enzyme activity. After a 5 min incubation period, the absorbance was measured at 340 nm. The inhibitory activities of the synthesized compounds were determined by comparing the enzyme activity in the presence and absence of the test compounds. The percentage inhibition and IC_50_ values were calculated from concentration-dependent inhibition curves according to literature [62] (Figure 10).

#### 3.2.3. Spectrophotometric Determination of AChE Activity

The acetylcholinesterase (AChE; EC 3.1.1.7) inhibitory activities of the synthesized compounds were determined spectrophotometrically using Ellman’s colorimetric method with minor modifications [63]. The assay is based on the hydrolysis of acetylthiocholine iodide (ATCI) by AChE to produce thiocholine, which subsequently reacts with 5,5′-dithiobis(2-nitrobenzoic acid) (DTNB, Ellman’s reagent) to form the yellow-colored 5-thio-2-nitrobenzoate (TNB) anion. The formation of TNB was monitored by measuring the increase in absorbance at 412 nm. The reaction mixture consisted of 100 mM sodium phosphate buffer (pH 7.5), AChE enzyme solution. The enzyme was prepared as a 1 mg/mL protein stock solution and 50 µL of the enzyme preparation was used in a final reaction volume of 1.0 mL, corresponding to a final enzyme activity of 10–50 U/mL. The assay was performed according to the method of Ellman et al. [63], using 1 M Tris-HCl containing 5 mM EDTA (pH 8.0) as the reaction buffer. Acetylthiocholine iodide (ATCI; 10 mM stock solution) was added at 20 µL, giving a final concentration of 0.20 mM. DTNB (10 mM stock solution) was added at 50 µL, giving a final concentration of 0.50 mM. The reaction was monitored spectrophotometrically at 412 nm. Control assays containing all reaction components except the test compound were performed under identical experimental conditions to determine the basal enzyme activity [64]. The inhibitory activity of each compound was expressed as the percentage inhibition relative to the control, and IC_50_ values were calculated from concentration–response curves by nonlinear regression analysis [62] (Figure 10).

### 3.3. Molecular Docking

Molecular docking provides a structure-based framework for examining how bioactive compounds can be accommodated within the binding pocket of a target protein and for identifying the non-covalent interactions that may contribute to ligand recognition and inhibitory activity [65]. In the present study, the docking analysis was designed as a mechanistic complement to the in vitro AChE inhibition experiments, in which several synthesized derivatives exhibited pronounced inhibitory activity in the low micromolar range. Rather than being used as an independent screening approach, molecular docking was therefore employed to rationalize the experimentally observed activity profile, compare the binding characteristics of the synthesized compounds with those of the reference inhibitor, and identify structural features that may contribute to AChE recognition.

Molecular docking calculations were carried out to characterize the binding orientations and interaction patterns of the synthesized derivatives within the active-site gorge of human acetylcholinesterase (hAChE). The X-ray crystal structure of hAChE (PDB ID: 4EY7, 2.35 Å resolution) was retrieved from the Protein Data Bank and used as the receptor model. Receptor preparation was performed in UCSF ChimeraX [66]. Missing structural regions were completed using the comparative modeling tools available in ChimeraX, and the receptor was subsequently processed using the DockPrep module. During preparation, hydrogen atoms were added, protonation states were adjusted, partial charges and atom types were assigned, and non-essential solvent molecules and heteroatoms were removed where appropriate.

The synthesized ligands were constructed in ChemDraw (version 26.0) and subjected to geometry optimization in Avogadro (version 2.0.0) using the MMFF94 force field [67]. The co-crystallized AChE inhibitor donepezil was used as the reference ligand for docking protocol validation. The docking search space was defined on the basis of the experimentally determined coordinates of the co-crystallized donepezil, thereby restricting the calculations to the established AChE binding gorge rather than employing a blind-docking strategy. This grid definition was designed to encompass the catalytically relevant ligand-binding region and adjacent aromatic subsites involved in inhibitor recognition.

Docking simulations were conducted using AutoDock 4.2.6 [68]. Prior to docking, Gasteiger partial charges were assigned to the ligands, nonpolar hydrogen atoms were merged, and ligand rotatable bonds were defined. The docking search was performed using the Lamarckian Genetic Algorithm (LGA) with a population size of 150, a maximum of 2,500,000 energy evaluations, and 54,000 generations. For each ligand, 150 independent docking runs were performed. A 40 × 40 × 40 grid box with a grid spacing of 0.375 Å was centered on the co-crystallized donepezil binding site at x = −14.108, y = −43.833, and z = 27.670 Å. The resulting poses were clustered using an RMSD tolerance of 2.0 Å and ranked according to the AutoDock-predicted binding energies. The docking protocol was validated by redocking the co-crystallized donepezil into the hAChE binding gorge.

The docking protocol was validated by re-docking donepezil into its crystallographic binding site and comparing the predicted pose with the experimentally observed ligand conformation. Following protocol validation, all synthesized compounds **3a**–**3g**, **4a**–**4g**, and **5a**–**5g** were docked into the same binding region under identical search conditions. Docked complexes were visually inspected in UCSF ChimeraX and BIOVIA Discovery Studio Visualizer [69]. Protein–ligand contacts, including conventional hydrogen bonds, hydrophobic interactions, π-mediated contacts, and other non-covalent interactions within the AChE binding gorge, were examined to support subsequent structural interpretation and selection of representative complexes for molecular dynamics simulations.

### 3.4. Molecular Dynamics Simulations

Molecular dynamics (MD) simulations were performed to complement the static information obtained from molecular docking and to investigate the structural stability, conformational behavior, and persistence of protein–ligand interactions over time [70]. Based on the experimental AChE inhibition profile together with the docking results, hAChE–**3e**, hAChE–**3f**, and hAChE–donepezil complexes were simulated using a single 100 ns production trajectory; independent replicate trajectories were not generated.

All simulations were carried out using GROMACS 2024.1. The receptor protein was parameterized with the AMBER14SB force field, while ligand topologies and parameters were generated using ACPYPE with the General AMBER Force Field (GAFF) and AM1-BCC partial atomic charges [71]. Each protein–ligand complex was placed in a triclinic simulation box and solvated explicitly with TIP3P water molecules, maintaining a minimum distance of 1.0 nm between the solute and the box boundaries. Counterions were added to neutralize the systems, and the ionic strength was adjusted to 0.15 M NaCl.

Long-range electrostatic interactions were treated using the Particle Mesh Ewald (PME) method [72], while both Coulombic and van der Waals interactions employed a 1.0 nm cutoff. All covalent bonds were constrained using the LINCS algorithm [73], and a 2 fs integration time step was used. Prior to equilibration, each solvated system was subjected to energy minimization using the steepest-descent algorithm to remove unfavorable contacts and steric clashes.

The minimized systems were subsequently equilibrated in two consecutive stages. First, 125 ps NVT equilibration was performed at 300 K using the V-rescale thermostat, followed by 125 ps NPT equilibration at 1 bar using the Parrinello–Rahman barostat. Production MD simulations were then carried out for 100 ns under periodic boundary conditions, with temperature and pressure maintained using the V-rescale thermostat and Parrinello–Rahman barostat, respectively. Compressed trajectory coordinates were recorded every 2 ps for subsequent analysis.

The generated trajectories were analyzed to assess both global protein stability and ligand behavior within the AChE binding gorge. Root-mean-square deviation (RMSD) was calculated for the protein backbone and bound ligands, while root-mean-square fluctuation (RMSF) was used to evaluate residue-level flexibility. Changes in overall protein compactness and solvent exposure were examined through the radius of gyration (Rg) and solvent-accessible surface area (SASA), respectively. In addition, protein–ligand hydrogen-bond analyses were performed to evaluate the persistence of intermolecular interactions throughout the simulations and to compare the dynamic behavior of compounds **3e** and **3f** with that of the reference inhibitor donepezil.

### 3.5. Binding Free Energy Calculations

Binding free energies of the hAChE–**3e**, hAChE–**3f**, and hAChE–donepezil complexes were estimated using the Molecular Mechanics/Generalized Born Surface Area (MM/GBSA) approach implemented in gmx_MMPBSA [60]. For each complex, the final 20 ns of the 100 ns production trajectory were considered for the calculations. A total of 200 snapshots, sampled at 100 ps intervals, were used to obtain representative binding-energy estimates.

The binding free energy was calculated as the sum of molecular mechanics and solvation contributions:

Δ*G*_bind_ = Δ*E*_vdW_ + Δ*E*_ele_ + Δ*G*_GB_ + Δ*G*_SA_

where Δ*E*_vdW_ and Δ*E*_ele_ represent the van der Waals and electrostatic interaction energies, respectively, whereas Δ*G*_GB_ and Δ*G*_SA_ correspond to the polar and nonpolar solvation contributions. The polar solvation energy was evaluated using the Generalized Born model with igb = 5. The internal and external dielectric constants were set to 1.0 and 80.0, respectively, and the ionic strength was defined as 0.150 M.

To further characterize the energetic determinants of ligand recognition, per-residue energy decomposition was performed using the decomposition scheme specified by idecomp = 2. This analysis was used to identify individual AChE residues contributing favorably or unfavorably to ligand binding and to compare the residue-level energetic profiles of **3e**, **3f**, and donepezil. Entropic contributions were not included; therefore, the calculated MM/GBSA values were interpreted comparatively rather than as absolute experimental binding free energies.

### 3.6. In Silico ADME and Drug-likeness Prediction

The physicochemical, pharmacokinetic, and drug-likeness properties of the representative lead compounds **3e**, **3f**, and **4a** were predicted using the SwissADME web server [74]. These compounds were selected to represent the most potent AChE inhibitor (**3e**), the compound with a balanced AChE/GST inhibitory profile (**3f**), and the most potent GST inhibitor (**4a**). The evaluated parameters included molecular weight (MW), topological polar surface area (TPSA), consensus LogP, gastrointestinal (GI) absorption, blood–brain barrier (BBB) permeation, P-glycoprotein (P-gp) substrate prediction, Lipinski rule violations, and bioavailability score. These predictions were used as complementary estimates of the physicochemical and pharmacokinetic profiles of the selected compounds and were not interpreted as substitutes for experimental pharmacokinetic measurements (Table S2).

## 4. Conclusions

In this study, a series of novel 1,2,4-triazole-based benzenesulfonate derivatives was synthesized and structurally elucidated using FT-IR, ^1^H NMR, ^13^C NMR, and LC-Q-TOF-MS techniques. The biological screening revealed inhibitory effects against acetylcholinesterase (AChE) and glutathione S-transferase (GST). Among the investigated compounds, **3e** exhibited the strongest AChE inhibition, with an IC_50_ value of 1.60 ± 0.198 μM, whereas **4a** showed the highest GST inhibitory activity (IC_50_ = 1.95 ± 0.492 μM), displaying greater inhibition than the reference compound ethacrynic acid. Selected derivatives also produced cytotoxic effects in HepG2 cells after 24 and 48 h of exposure.

To further investigate the molecular basis of AChE inhibition, molecular docking, molecular dynamics simulations, and binding free energy calculations were performed. The computational findings indicated that **3e** and **3f** maintained stable interactions within the hAChE active-site gorge, with **3e** exhibiting the most favorable computational characteristics among the examined derivatives. Per-residue energy decomposition highlighted the contribution of several amino acid residues associated with ligand recognition and stabilization in the catalytic gorge. While the calculated MM/GBSA binding energies do not directly establish experimental inhibitory potency, the integrated computational results, together with the biochemical assays, identify **3e** and **3f** as noteworthy AChE-active candidates. These findings provide a basis for further structural optimization and investigation of the biological potential of this class of compounds.

## Figures and Tables

**Table 1:** Enzyme Inhibition compounds (**3**–**5**) toward AChE and GST.

Compounds	AChE		GST	
	IC_50_(µM)	R^2^	IC_50_(µM)	R^2^
**3a**	2.78 ± 0.215	0.9823	3.41 ± 1.158	0.994
**3b**	2.64 ± 0.159	0.987	2.36 ± 0.341	0.9935
**3c**	2.45 ± 0.212	0.9954	3.3 ± 0.948	0.9602
**3d**	2.38 ± 0.235	0.991	3 ± 1.025	0.9924
**3e**	1.60 ± 0.198	0.9701	2.78 ± 0.210	0.9944
**3f**	1.92 ± 0.291	0.9929	2.32 ± 0.810	0.981
**3g**	2.46 ± 0.541	0.9931	2.02 ± 0.229	0.9991
**4a**	2.53 ± 0.452	0.9851	1.95 ± 0.492	0.9931
**4b**	2.39 ± 0.154	0.9953	2.31 ± 1.068	0.9973
**4c**	2.24 ± 0.265	0.9986	3 ± 0.680	0.9937
**4d**	2.02 ± 0.271	0.9768	3.17 ± 0.787	0.9994
**4e**	1.74 ± 0.765	0.9689	2.39 ± 0.258	0.9869
**4f**	2.55 ± 0.445	0.9898	3.61 ± 0.216	0.987
**4g**	1.96 ± 0.134	0.9976	2.96 ± 0.357	0.9878
**5a**	2.41 ± 0.457	0.967	3.22 ± 0.419	0.9531
**5b**	3 ± 0.912	0.9915	3.26 ± 0.810	0.998
**5c**	2.41 ± 0.658	0.9682	1.98 ± 0.420	0.9875
**5d**	1.96 ± 0.475	0.9913	2.71 ± 0.379	0.9888
**5e**	2.02 ± 0.179	0.984	2.51 ± 0.716	0.9914
**5f**	2.14 ± 0.814	0.9987	3.06 ± 0.225	0.9995
**5g**	2.47 ± 0.759	0.984	2.81 ± 0.480	0.9807
**DNP**	0.2017 ± 0.0036	0.9938	-	-
**INN**	-	-	3.066 ± 0.475	0.9751

**Table 2:** Molecular docking and interaction profiles of compounds **3e** and **3f** and the reference inhibitor donepezil against human acetylcholinesterase (hAChE).

Ligand	Binding Energy (kcal/mol)	H-Bonds (*n*)	Key Interacting Residues
**3e**	−14.67	3	Trp86, Leu289, Ser293, Val294, Phe295, Arg296, Phe297, Tyr341, Tyr337, His447
**3f**	−13.62	3	Trp86, Leu289, Gln291, Ser293, Val294, Phe295, Arg296, Phe297, Tyr337, Tyr341, His447
donepezil *	−11.27	1	Tyr72, Trp86, Trp286, Phe338, Ser293, Phe295, Tyr337, Tyr341, His447

* reference compound.

**Table 3:** MM/GBSA binding free energy components of the hAChE–**3e**, hAChE–**3f**, and hAChE–donepezil complexes calculated from the final 20 ns of the MD trajectories. Values are reported as mean ± SD in kcal/mol.

Complex	Δ*E*_vdW_ (kcal/mol)	Δ*E*_ele_ (kcal/mol)	Δ*G*_GB_ (kcal/mol)	Δ*G*_SA_ (kcal/mol)	Δ*G*_bind_ (kcal/mol)
hAChE–**3e**	−54.01 ± 3.02	−18.36 ± 3.47	43.80 ± 2.90	−6.58 ± 0.24	−35.15 ± 3.24
hAChE–**3f**	−55.63 ± 2.14	−6.62 ± 4.85	44.29 ± 3.59	−7.38 ± 0.22	−25.34 ± 3.18
hAChE–donepezil	−60.29 ± 2.55	−233.25 ± 7.09	246.61 ± 7.03	−6.43 ± 0.14	−53.35 ± 2.99

## Data Availability

The original contributions presented in this study are included in the article/Supplementary Materials. Further inquiries can be directed to the corresponding author.

## Supplementary Materials

The following supporting information can be downloaded at: https://www.mdpi.com/article/10.3390/ph19101645/s1, Figures S1–S21: ^1^H-NMR, ^13^C-NMR (APT) and LC Q-TOF spectrum of compound **3a**–**3g**; Figures S22–S42: ^1^H-NMR, ^13^C-NMR (APT) and LC Q-TOF spectrum of compound **4a**–**4g**; Figures S43–S61: ^1^H-NMR, ^13^C-NMR (APT) and LC Q-TOF spectrum of compound **5a**–**5g**; Figure S62: IC_50_ inhibition curves of the synthesized compounds and reference inhibitors against the investigated enzymes; Table S1: Predicted binding energies of the synthesized compounds against human acetylcholinesterase (hAChE); Table S2: Predicted physicochemical, pharmacokinetic, and drug-likeness properties of selected lead compounds using SwissADME.

## References

[1] Al-Masoudi I.A., Al-Soud Y.A., Al-Salihi N.J., Al-Masoudi N.A. (2006). 1,2,4-Triazoles: Synthetic approaches and pharmacological importance. Chem. Heterocycl. Compd..

[2] Moulin A., Bibian M., Blayo A.-L., El Habnouni S., Martinez J., Fehrentz J.-A. (2010). Synthesis of 3,4,5-Trisubstituted-1,2,4-triazoles. Chem. Rev..

[3] Hitotsuyanagi Y., Motegi S., Fukaya H., Takeya K. (2002). A cis amide bond surrogate incorporating 1,2,4-triazole. J. Org. Chem..

[4] Blayo A.-L., Brunel F., Martinez J., Fehrentz J.-A. (2011). Synthesis of various chiral 1,2,4-triazole-containing α-amino acids from aspartic or glutamic acids. Eur. J. Org. Chem..

[5] Banerjee S., Ganguly S., Sen K.K., India B.W.B. (2013). A review on 1,2,4-triazoles. J. Adv. Pharm. Educ. Res..

[6] Kumar S.S., Kavitha H.P. (2013). Synthesis and Biological Applications of 1,2,4-Triazole Derivatives–A Review. Mini-Rev. Org. Chem..

[7] Hassan A.S., Moustafa G.O., Askar A.A., Naglah A.M., Al-Omar M.A. (2018). Synthesis and antibacterial evaluation of fused pyrazoles and Schiff-bases. Synth. Commun..

[8] Hranjec M., Starcevic K., Pavelic S.K., Pero Lucin P., Paveli K., Zamola G.K. (2011). Synthesis, spectroscopic characterization and antiproliferative evaluation in vitro of novel Schiff bases related to benzimidazoles. Eur. J. Med. Chem..

[9] Wilkinson S.M., Sheedy T.M., New E.J. (2016). Synthesis and characterization of metal complexes with Schiff base ligands. J. Chem. Educ..

[10] Singh K., Barwa M.S., Tyagi P. (2006). Synthesis, characterization and biological studies of Co(II), Ni(II), Cu(II) and Zn(II) complexes with bidentate Schiff bases derived by heterocyclic ketone. Eur. J. Med. Chem..

[11] Tadavi S.K., Rajput J.D., Bagul S.D., Hosamani A.A., Sangshetti J.N., Bendre R.S. (2017). Synthesis, crystal structures, biological screening and electrochemical analysis of some salen-based transition metal complexes. Res. Chem. Intermed..

[12] Chu X.-M., Wang C., Wang W.-L., Liang L.-L., Liu W., Gong K.-K., Sun K.-L. (2019). Triazole derivatives and their antiplasmodial and antimalarial activities. Eur. J. Med. Chem..

[13] Romero A.H., Sojo F., Arvelo F., Calderón C., Morales A., López S.E. (2020). Anticancer potential of new 3-nitroaryl-6-(N-methyl)piperazin-1,2,4-triazolo[3,4-a]phthalazines targeting voltage-gated K+ channel: Copper-catalyzed one-pot synthesis from 4-chloro-1-phthalazinyl-arylhydrazones. Bioorg. Chem..

[14] Veloso R.V., Shamim A., Lamarrey Y., Stefani H.A., Sciani J.M. (2021). Antioxidant and anti-sickling activity of glucal-based triazoles compounds–An in vitro and in silico study. Bioorg. Chem..

[15] Süleymanoğlu N., Ustabaş R., Ünver Y., Bingöl Alpaslan Y., Direkel Ş., Karaman Ü. (2019). 5-Phenyl thiophene amino phenol derivatives: Synthesis, spectroscopic characterization, computational study and antimicrobial activity. J. Mol. Struct..

[16] Fuh M.T., Tseng C.C., Li S.M., Tsai S.E., Chuang T.J., Lu C.H., Yang Y.C., Tsai H.J., Wong F.F. (2021). Design, synthesis and biological evaluation of glycolamide, glycinamide, and β-amino carbonyl 1,2,4-triazole derivatives as DPP-4 inhibitors. Bioorg. Chem..

[17] Jahani R., Abtahi S.R., Nematpour M., Dastjerdi H.F., Chamanara M., Hami Z., Paknejad B. (2020). Design, synthesis, and pharmacological evaluation of novel 1,2,4-triazol-3-amine derivatives as potential agonists of GABA_A subtype receptors with anticonvulsant and hypnotic effects. Bioorg. Chem..

[18] Shahzad S.A., Yar M., Khan Z.A., Shahzadi L., Naqvi S.A.R., Mahmood A., Ullah S., Shaikh A.J., Sherazi T.A., Bale A.T. (2019). Identification of 1,2,4-triazoles as new thymidine phosphorylase inhibitors: Future anti-tumor drugs. Bioorg. Chem..

[19] Mahmoudi Y., Badali H., Hashemi S.M., Ansari M., Fakhim H., Fallah M., Shokrzadeh M., Emami S. (2019). New potent antifungal triazole alcohols containing N-benzylpiperazine carbodithioate moiety: Synthesis, in vitro evaluation and in silico study. Bioorg. Chem..

[20] Bakri Y.E., Marmouzi I., Jemli M.E., Anouar E.H., Karthikeyan S., Harmaoui A., Faouzi M.E.A., Mague J.T., Essassi E.M. (2019). Synthesis, biological activity and molecular modeling of a new series of condensed 1,2,4-triazoles. Bioorg. Chem..

[21] Alagarsamy V., Rupeshkumar M., Kavitha K., Meena S., Shankar D., Siddiqui A.A., Rajesh R. (2008). Synthesis and pharmaco-logical investigation of novel 4-(2-methylphenyl)-1-substituted-4H-[1,2,4]triazolo[4,3-a]quinazolin-5-ones as new class of H1-antihistaminic agents. Eur. J. Med. Chem..

[22] Küçükgüzel Ş.G., Çıkla-Süzgün P. (2015). Recent advances bioactive 1,2,4-triazole-3-thiones. Eur. J. Med. Chem..

[23] Li Q., Qiu L., Tan W., Gu G., Guo Z. (2017). Novel 1,2,3-triazolium-functionalized inulin derivatives: Synthesis, free radical-scavenging activity, and antifungal activity. RSC Adv..

[24] Ünver Y., Tuluk M., Kahriman N., Emirik M., Bektaş E., Direkel Ş. (2019). New Chalcone Derivatives With Schiff Base-Thiophene: Synthesis, Biological Activity And Molecular Docking Studies. Russ. J. Gen. Chem..

[25] Han F., Teng Q., Shen Q., Wang Y., Zhang Y. (2011). Influence of Schiffbase and lanthanide metals on the synthesis, stability, and reactivity of monoamido lan- thanide complexes bearing two Schiff-bases. Inorg. Chem. J..

[26] Ustabaş R., Süleymanoğlu N., Ünver Y., Direkel Ş. (2020). 5-(4-Bromobenzyl)-4-(4-(5-phenyl-1,3,4-oxadiazole-2-yl)phenyl)-2,4-dihydro-3H-1,2,4-triazole-3-one: Synthesis, characterization, DFT study and antimicrobial activity. J. Mol. Struct..

[27] Süleymanoğlu N., Demir E.E., Direkel Ş., Ünver Y. (2020). Theoretical Study and Antimicrobial Activities of New Schiff Base Derivatives with Thiophene. J. Mol. Struct..

[28] Bal T.R., Anand B., Yogeeswari P., Sriram D. (2005). Synthesis and evaluation of anti-HIV activiy of isatin β-thiosemicarbazone derivatives. Bioorg. Med. Chem. Lett..

[29] Bendre R.S., Tadavi S.K., Yadav A.A. (2017). Synthesis and characterization of a novel Schiffbase of 1,2-diaminopropane with substituted salicyaldehyde and its transition metal complexes-Single crystal structures and biological activities. J. Mol. Struct..

[30] Wadher S.J., Puranik M.P., Karande N.A., Yeole P.G. (2009). Synthesis and biological evaluation of Schiff base of dapsone and their derivative as antimicrobial agents. Int. J. Pharm. Tech. Res..

[31] Chinnasamy R.P., Sundararajan R., Govindaraj S. (2010). Synthesis, characterization, and analgesic activity of novel schiff base of isatin derivatives. J. Adv. Pharm. Technol. Res..

[32] Li Y., Yang Z.S., Zhang H., Cao B.J., Wang F.D., Zhang Y., Shi Y., Yang J., Wu B. (2003). Artemisinin derivatives bearing Mannich base group: Synthesis and antimalarial activity. Bioorg. Med. Chem..

[33] Evecen M., Çelik F., Ünver Y., Güler H.B. (2025). 4-Bromobenzene Sulfonate Derivatives: Synthesis, Characterization, DFT and Molecular Docking Study. Acta Chim..

[34] Ünver Y., Evecen M., Çelik F., Aydın A., Güler H.A., İnan Bektaş K.İ., Usta T. (2026). Novel Sulfonate Derivatives Functionalized with Triazole–Hydrazone Moieties: Synthesis, Characterization, DFT, Targeting Brain Tumor via DNA Damage, Cytotoxicity, and MigrationSuppression and Antimicrobial Activity and In siliko Study. Molecules.

[35] Ünver Y., Evecen M., Çelik F., Aydın A., Güler H.A., İnan Bektaş K.İ., Usta T., Öztürk Ö.F. (2027). Synthesis, DFT, In Silico, Antimicrobial and Anticancer Studies of Triazole–Hydrazone Sulfonate Derivatives Targeting Brain Tumors via DNA Damage, Cytotoxicity and Migration Suppression. J. Mol. Struct..

[36] Karthikeyan M.S., Prasad D.J., Poojary B., Bhat K.S., Holla B.S., Kumari N.S. (2006). Synthesis and biological activity of Schiff and Mannich bases bearing 2,4-dichloro-5-fluorophenyl moiety. Bioorg. Med. Chem..

[37] Rozbicki P., Oğuz E., Wolińska E., Türkan F., Cetin A., Branowska D. (2024). Synthesis and examination of 1,2,4-triazine-sulfonamide hybrids as potential inhibitory drugs: Inhibition effects on AChE and GST enzymes in silico and in vitro conditions. Arch. Pharm..

[38] Adigüzel R., Türkan F., Yıldiko Ü., Aras A., Evren E., Önkol T. (2021). Synthesis and in silico studies of novel Ru(II) complexes of Schiff base derivatives of 3-[(4-amino-5-thioxo-1,2,4-triazole-3-yl)methyl]-2(3H)-benzoxazolone compounds as potent glutathione S-transferase and cholinesterases inhibitor. J. Mol. Struct..

[39] Türkan F., Cetin A., Rozbicki P., Oğuz E., Wolińska E., Branowska D. (2024). Pharmacological assessment of disulfide–triazine hybrids: Synthesis, enzyme inhibition, and molecular docking study. Med. Chem. Res..

[40] Bolshette N.B., Thakur K.K., Bidkar A.P., Trandafir C., Kumar P., Gogoi R. (2014). Protein folding and misfolding in the neurodegenerative disorders: A review. Rev. Neurol..

[41] Türkan F., Huyut Z., Atalar M.N. (2018). The toxicological impact of some avermectins on human erythrocytes glutathione S-transferase enzyme. J. Biochem. Mol. Toxicol..

[42] Turhan K., Pektaş B., Türkan F., Tuğcu F.T., Turgut Z., Taslimi P., Karaman H.S., Gülçin İ. (2020). Novel benzo[b]xanthene derivatives: Bismuth(III) triflate-catalyzed one-pot synthesis, characterization, and acetylcholinesterase, glutathione S-transferase, and butyrylcholinesterase inhibitory properties. Arch. Pharm..

[43] İlik Z.A., Cetin A., Türkan F. (2026). Green synthesis of tetrahydrodipyrazolopyridines: In silico and in vitro evaluation against HepG2 liver cancer cells. Chem. Sel..

[44] Kotan G., Aras A., Oğuz E., Türkan F., Yüksek H. (2026). Novel 1-morpholine-3-substituted triazol-5-ones: Synthesis and multi-biological evaluation on HepG2 and U87 cells with SAR and DFT studies. J. Mol. Struct..

[45] Oğuz E., Tülek G.S., Asadove E., Cetin A., Türkan F. (2026). Dual inhibition of AChE and GST by 1,2,4-triazine-3-amine: Integrated biochemical, computational, and hepatotoxicity insights. Biotechnol. Appl. Biochem..

[46] Tokalı F.S., Demir Y., Demircioğlu İ.H., Türkeş C., Kalay E., Şendil K., Beydemir Ş. (2022). Synthesis, biological evaluation, and in silico study of novel library sulfonates containing quinazolin-4(3H)-one derivatives as potential aldose reductase inhibitors. Drug Dev. Res..

[47] Ünver Y., Sancak K., Çelik F., Birinci E., Küçük M., Soylu S., Arslan Burnaz N. (2014). New thiophene-1,2,4-triazole-5(3)-ones: Highly bioactive thiosemicarbazides, structures of Schiff bases and triazole-thiols. Eur. J. Med. Chem..

[48] Beytur M., Tarmaşır E., Oğuz E., Bayrakdar A., Sabancılar İ., Akyıldırım O., Türkan F., Aras A., Yüksek H. (2026). 3-Substituted triazolone–benzoate hybrids: Synthesis, enzyme inhibition, anticancer activity and ADMET evaluation by molecular docking and dynamics studies. Drug Dev. Res..

[49] Çelik F., Ünver Y., Güler H.İ., Oğuz E., Türkan F. (2025). New methyl/benzyl-1,2,4-triazole-3-one derivatives: Synthesis, characterization (IR, NMR), antidiabetic, anti-Alzheimer, and molecular docking study. J. Biochem. Mol. Toxicol..

[50] Türkan F., Cetin A., Ustun M.A. (2025). Pyrimidine based inhibitors targeting glutathione S-transferase in phase II detoxification: Antioxidant, ADMET, docking, and molecular dynamics evaluation. J. Biochem. Mol. Toxicol..

[51] Cheung J., Rudolph M.J., Burshteyn F., Cassidy M.S., Gary E.N., Love J., Franklin M.C., Heigh J.J. (2012). Structures of human acetylcholinesterase in complex with pharmacologically important ligands. J. Med. Chem..

[52] Rosenberry T.L., Brazzolotto X., Macdonald I.R., Wandhammer M., Trovaslet-Leroy M., Darvesh S., Nachon F. (2017). Comparison of the binding of reversible inhibitors to human butyrylcholinesterase and acetylcholinesterase: A crystallographic, kinetic and calorimetric study. Molecules.

[53] De Boer D., Nguyen N., Mao J., Moore J., Sorin E.J. (2021). A comprehensive review of cholinesterase modeling and simulation. Biomolecules.

[54] Hung L.-W., Sanbonmatsu K.Y., Williams R.F., Chen J.C.-H. (2025). Acetylcholinesterase: Structure, dynamics, and interactions with organophosphorus compounds. Protein Sci..

[55] Bozkır İ., İbişoğlu M.S., Kayıkçıoğlu Bozkır İ., Güler H.İ. (2026). Computational prioritization of multi-target inhibitors: Explainable QSAR and docking-based discovery of dual AChE/BACE1 chemotypes. J. Comput. Aided Mol. Des..

[56] Sağlık B.N., Osmaniye D., Acar Çevik U., Levent S., Kaya Çavuşoğlu B., Özkay Y., Kaplancıklı Z.A. (2020). Design, synthesis, and structure–activity relationships of thiazole analogs as anticholinesterase agents for Alzheimer’s disease. Molecules.

[57] Girgin M., Isik S., Kantarci-Carsibasi N. (2023). Proposing novel natural compounds against Alzheimer’s disease targeting acetylcholinesterase. PLoS ONE.

[58] Ahmad V., Alotibi I., Alghamdi A.A., Ahmad A., Jamal Q.M.S., Srivastava S. (2024). Computational approaches to evaluate the acetylcholinesterase binding interaction with taxifolin for the management of Alzheimer’s disease. Molecules.

[59] Usta T., Çelik F., Güler H.İ., Bektaş K.İ., Kılıç M., Ünver Y. (2026). Novel bis-sulfonate Schiff base derivatives as dual AChE inhibitors and antimicrobial agents: Synthesis and molecular docking studies. Russ. J. Gen. Chem..

[60] Valdés-Tresanco M.S., Valdés-Tresanco M.E., Valiente P.A., Moreno E. (2021). gmx_MMPBSA: A new tool to perform end-state free energy calculations with GROMACS. J. Chem. Theory Comput..

[61] Honorio P., Hannongbua S., Saparpakorn P. (2022). Roles of hybrid donepezil scaffolds as potent human acetylcholinesterase inhibitors using in silico interaction analysis, drug-likeness, and pharmacokinetics prediction. Chem.-Biol. Interact..

[62] Habig W.H., Jakoby W.B. (1981). Assays for differentiation of glutathione S-transferases. Methods Enzymol..

[63] Ellman G.L., Courtney K.D., Andres V., Featherstone R.M. (1961). A new and rapid colorimetric determination of acetylcholinesterase activity. Biochem. Pharmacol..

[64] Ertaş Oğuz F., Babaoğlu A.R., Türkan F., Oğuz E., Demirel A.F., Paçal N. (2024). Investigation of interleukins and oxidative stress parameters in cows naturally infected with bovine viral diarrhea virus. Pak. Vet. J..

[65] Meng X.-Y., Zhang H.-X., Mezei M., Cui M. (2011). Molecular docking: A powerful approach for structure-based drug discovery. Curr. Comput. Aided Drug Des..

[66] Pettersen E.F., Goddard T.D., Huang C.C., Meng E.C., Couch G.S., Croll T.I., Morris J.H., Ferrin T.E. (2021). UCSF ChimeraX: Structure visualization for researchers, educators, and developers. Protein Sci..

[67] Hanwell M.D., Curtis D.E., Lonie D.C., Vandermeersch T., Zurek E., Hutchison G.R. (2012). Avogadro: An advanced semantic chemical editor, visualization, and analysis platform. J. Cheminform..

[68] Morris G.M., Huey R., Lindstrom W., Sanner M.F., Belew R.K., Goodsell D.S., Olson A.J. (2009). AutoDock4 and AutoDockTools4: Automated docking with selective receptor flexibility. J. Comput. Chem..

[69] Dassault Systèmes BIOVIA (2016). Discovery Studio Visualizer 2021.

[70] Hollingsworth S.A., Dror R.O. (2018). Molecular dynamics simulation for all. Neuron.

[71] Wang J., Wolf R.M., Caldwell J.W., Kollman P.A., Case D.A. (2004). Development and testing of a general Amber force field. J. Comput. Chem..

[72] Darden T., York D., Pedersen L. (1993). Particle mesh Ewald: An N·log(N) method for Ewald sums in large systems. J. Chem. Phys..

[73] Hess B., Bekker H., Berendsen H.J.C., Fraaije J.G.E.M. (1997). LINCS: A linear constraint solver for molecular simulations. J. Comput. Chem..

[74] Daina A., Michielin O., Zoete V. (2017). SwissADME: A free web tool to evaluate pharmacokinetics, drug-likeness and medicinal chemistry friendliness of small molecules. Sci. Rep..

