# Supplementary material for: Synthesis, Biological Evaluation, and Molecular Dynamics Studies of Novel 1,2,4-Triazole-Functionalized Benzene-Sulfonate Derivatives as Potential Enzyme Inhibitors and Cytotoxicity in HepG2 Cells
Source: Pharmaceuticals (Basel). 2026 Oct 19;19(10):1645. doi: 10.3390/ph19101645 (PMC13708584; doi:10.3390/ph19101645)
Supplement: Supplementary file 1 [file pharmaceuticals-19-01645-s001.zip › pharmaceuticals-4603013-supplementary.pdf]

Table S1. Predicted binding energies of the synthesized compounds against human acetylcholinesterase (hAChE).

Ligand Name	Binding Energy (kcal/mol)	Ligand Name	Binding Energy (kcal/mol)
3a	-13.65	4e	-14.06
3b	-13.53	4f	-13.19
3c	-14.17	4g	-14.03
3d	-14.30	5a	-13.58
3e	-14.67	5b	-13.43
3f	-13.62	5c	-13.82
3g	-13.84	5d	-14.18
4a	-13.48	5e	-14.24
4b	-13.10	5f	-13.28
4c	-14.06	5g	-13.60
4d	-13.85		

Table S2. Predicted physicochemical, pharmacokinetic, and drug-likeness properties of selected lead compounds using SwissADME.

Compound	MW (g/mol)	TPSA (Å²)	Consensus LogP	GI absorption	BBB permeant	P-gp substrate	Lipinski violations	Bioavailability score
3e	560.36	114.79	4.06	High	No	No	2	0.17
3f	464.49	124.02	3.27	Low	No	No	0	0.55
4a	448.49	114.79	3.53	High	No	No	0	0.55

Formulas of synthesized compounds

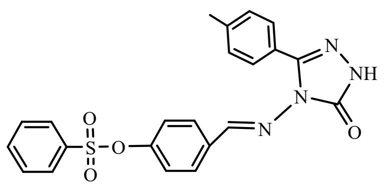

3a

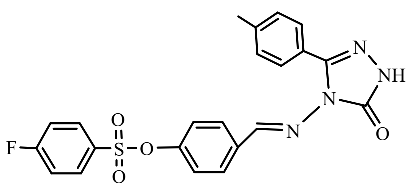

3b

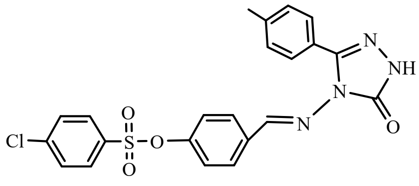

3c

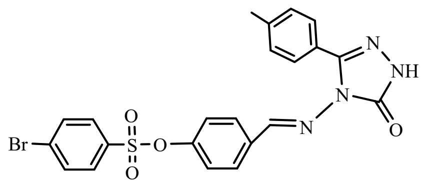

3d

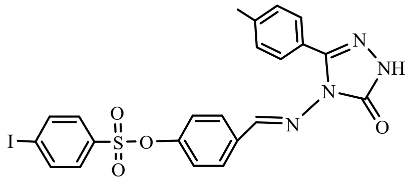

3e

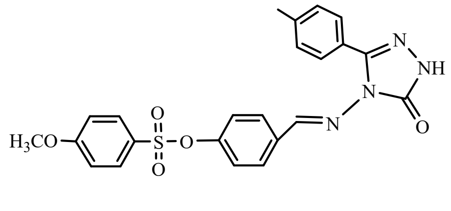

3f

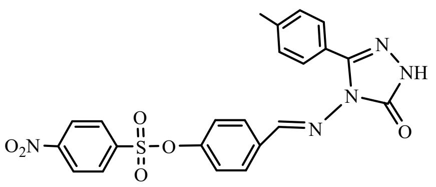

3g

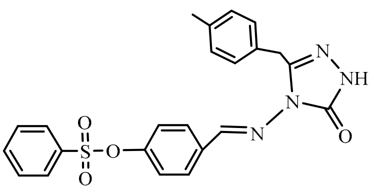

4a

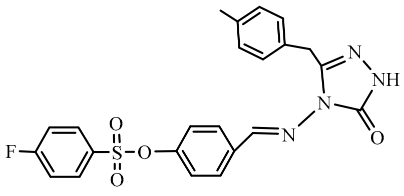

4b

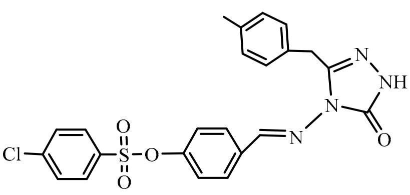

4c

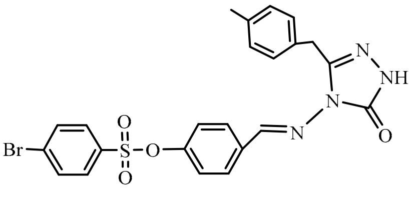

4d

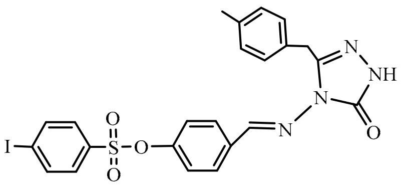

4e

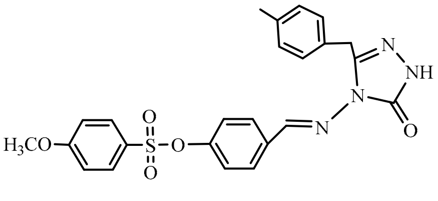

4f

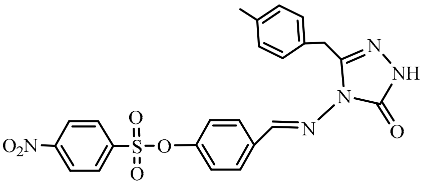

4g

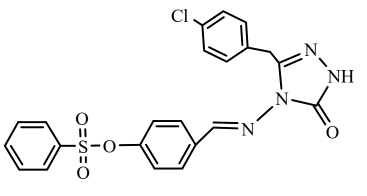

5a

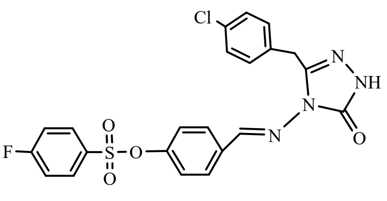

5b

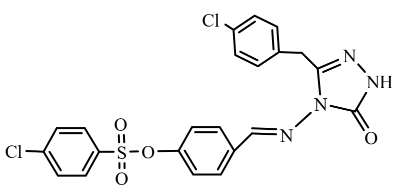

5c

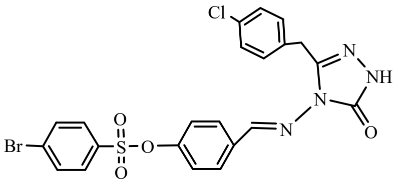

5d

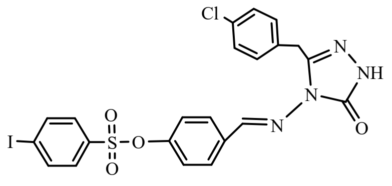

5e

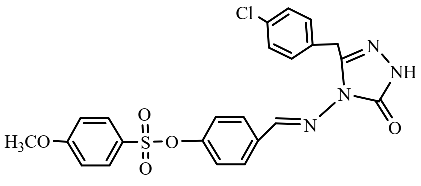

5f

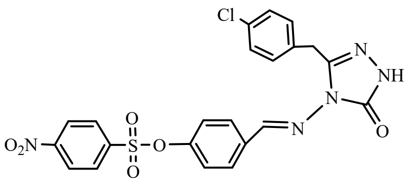

5g

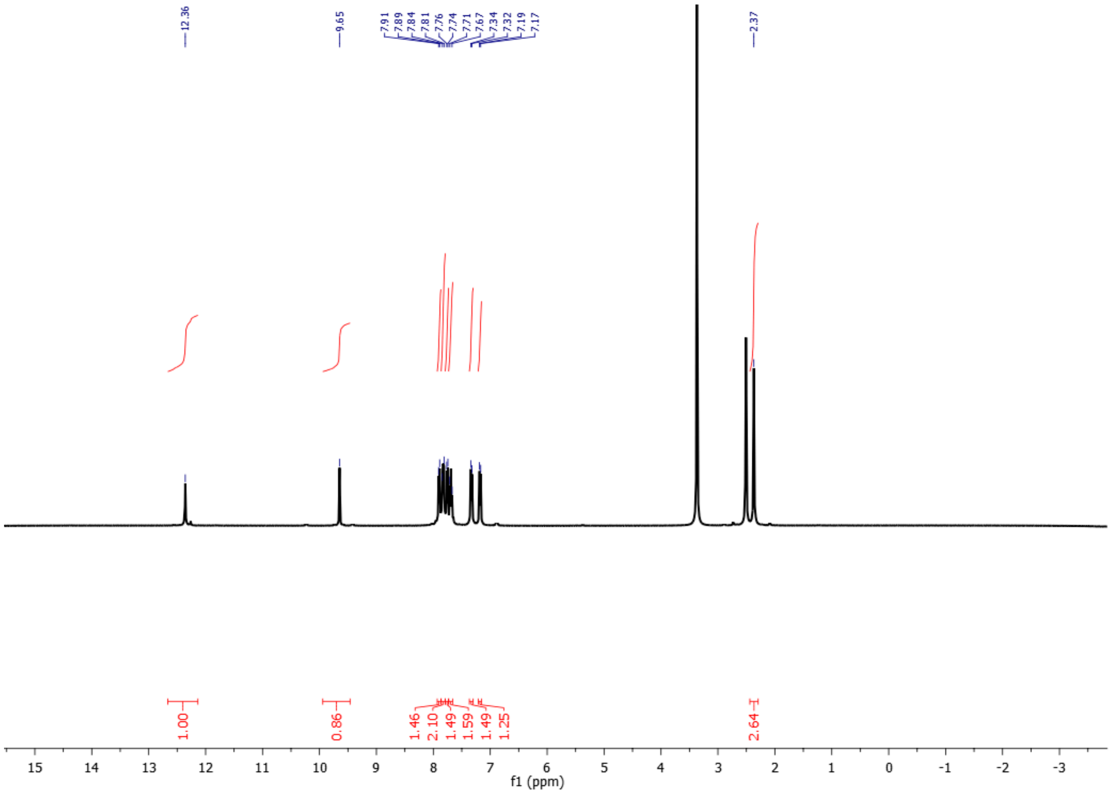

Figure S1. ^{1}H-NMR spectrum of compound 3a

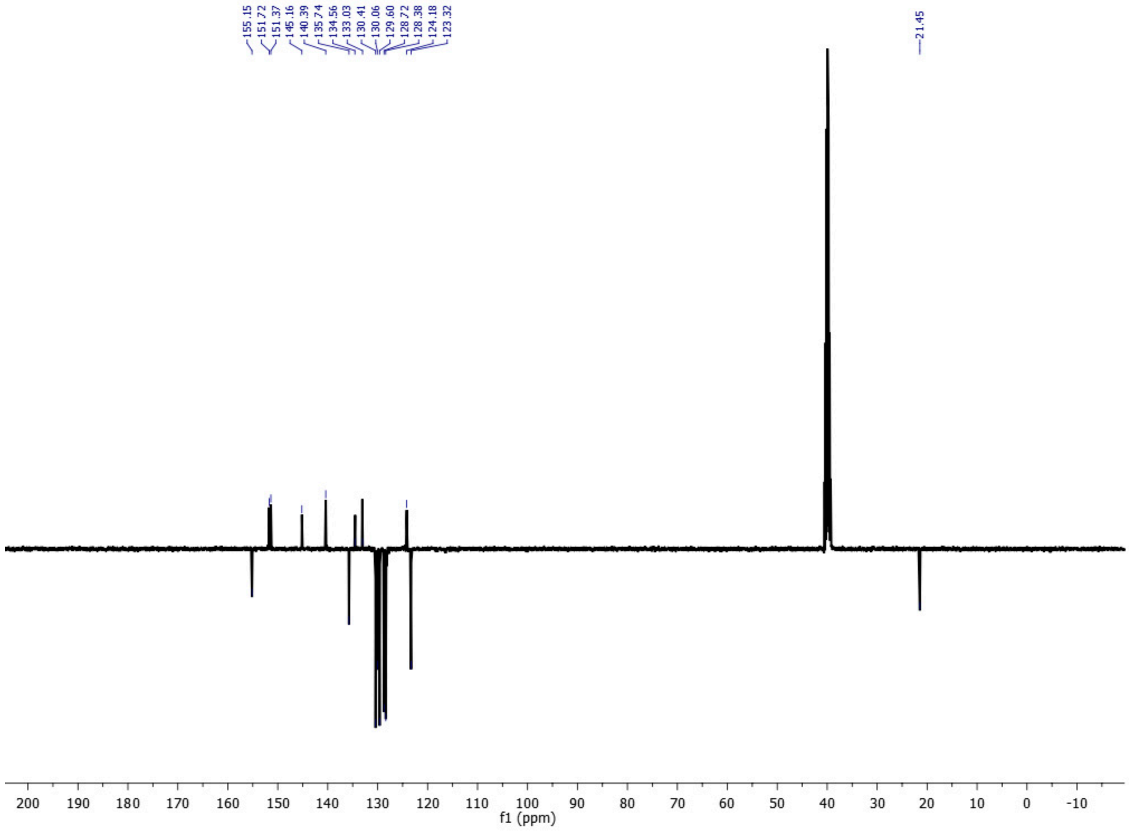

Figure S2. ^{13}C-NMR (APT) spectrum of compound 3a

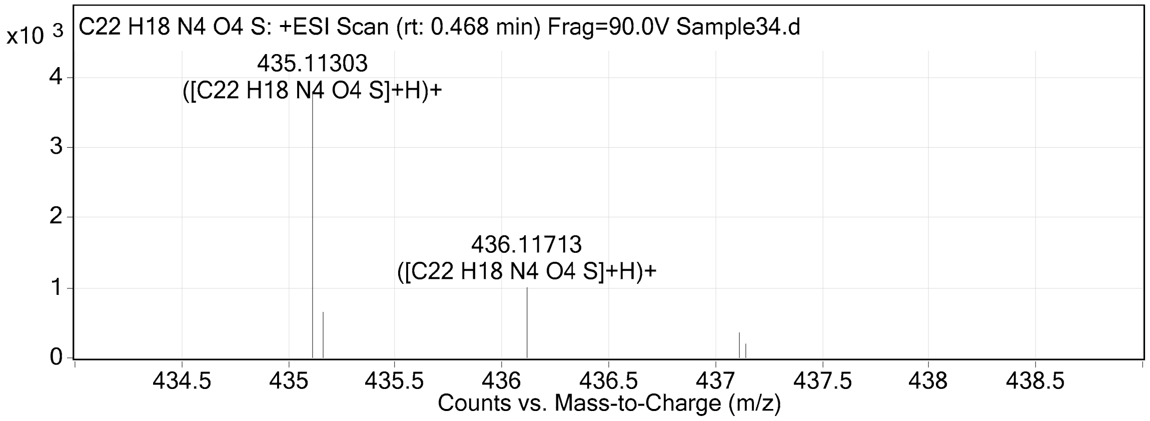

Figure S3. LC Q-TOF spectrum of compound 3a

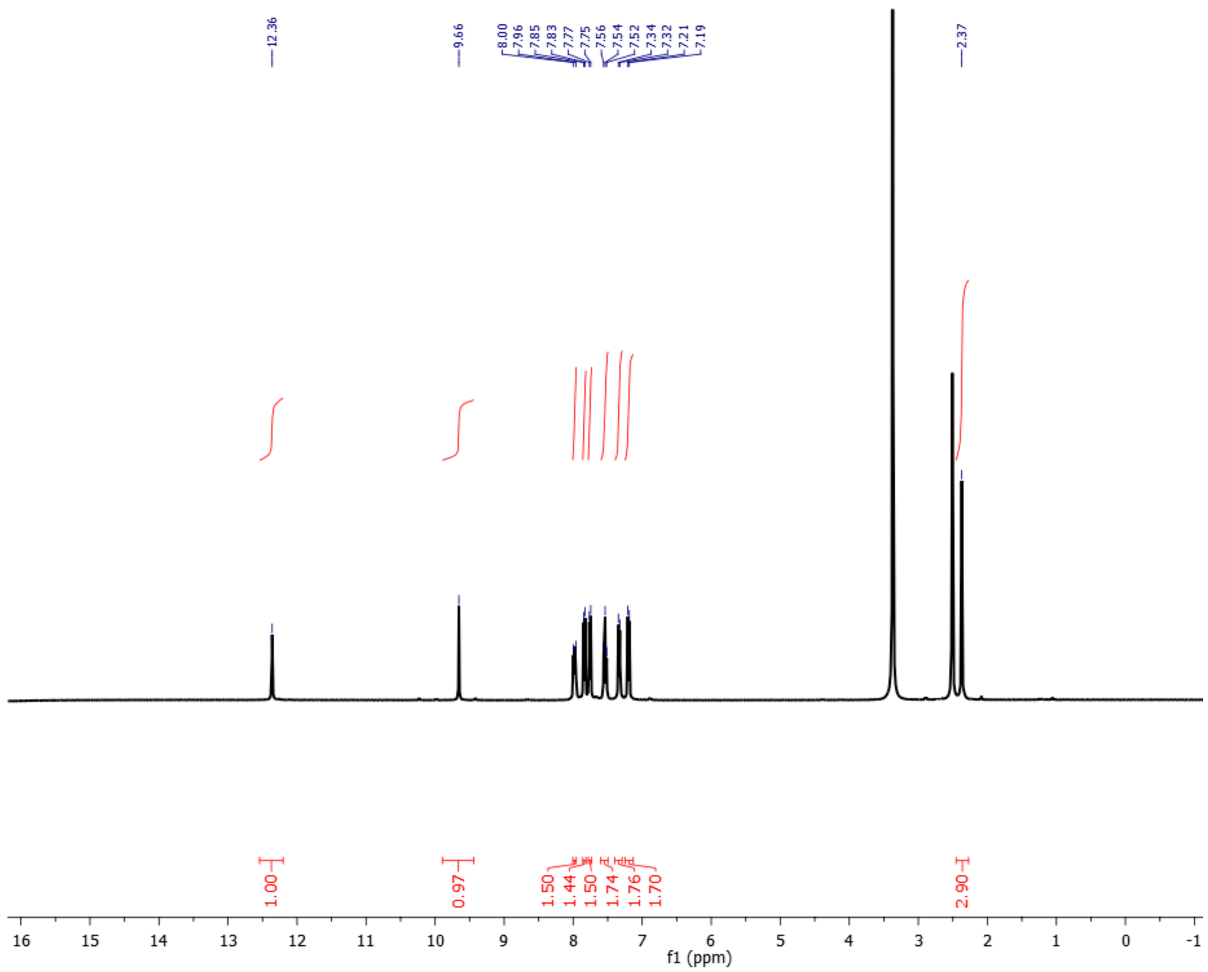

Figure S4. ^{1}H-NMR spectrum of compound 3b

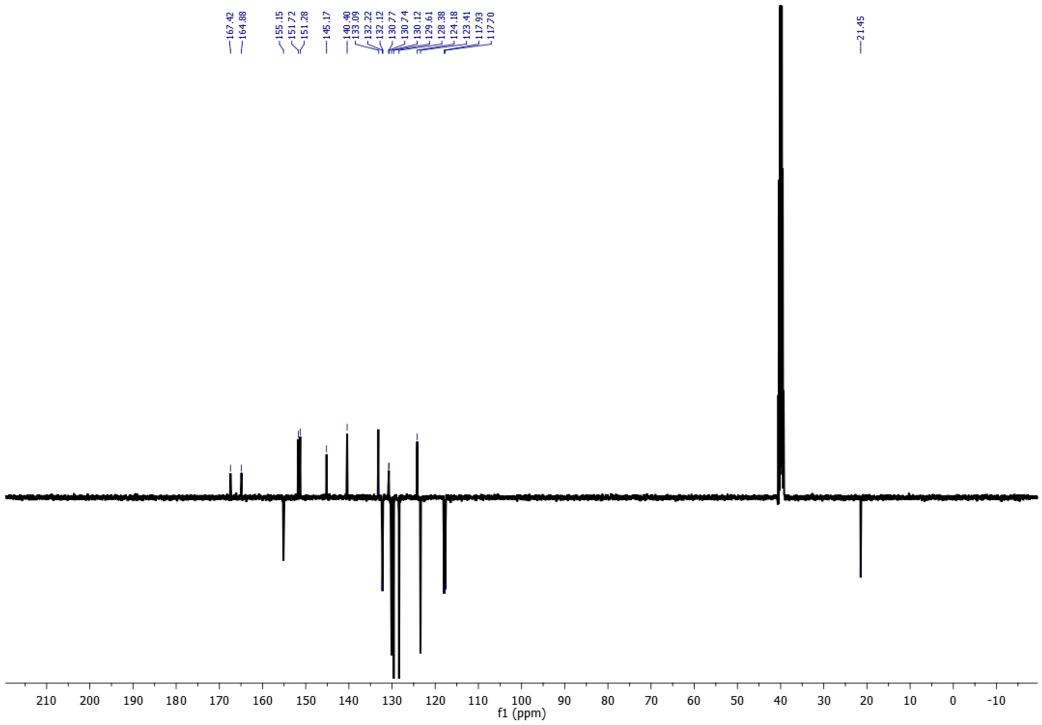

Figure S5. ^{13}C-NMR (APT) spectrum of compound 3b

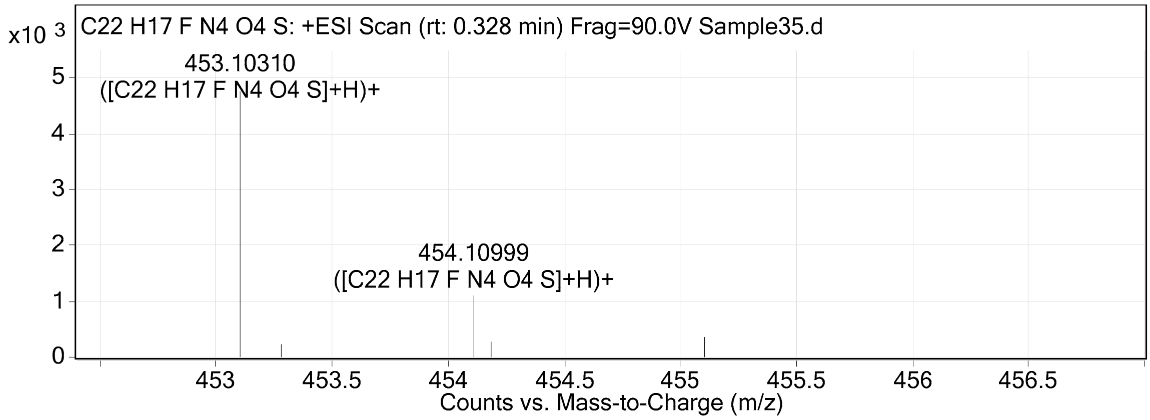

Figure S6. LC Q-TOF spectrum of compound 3b

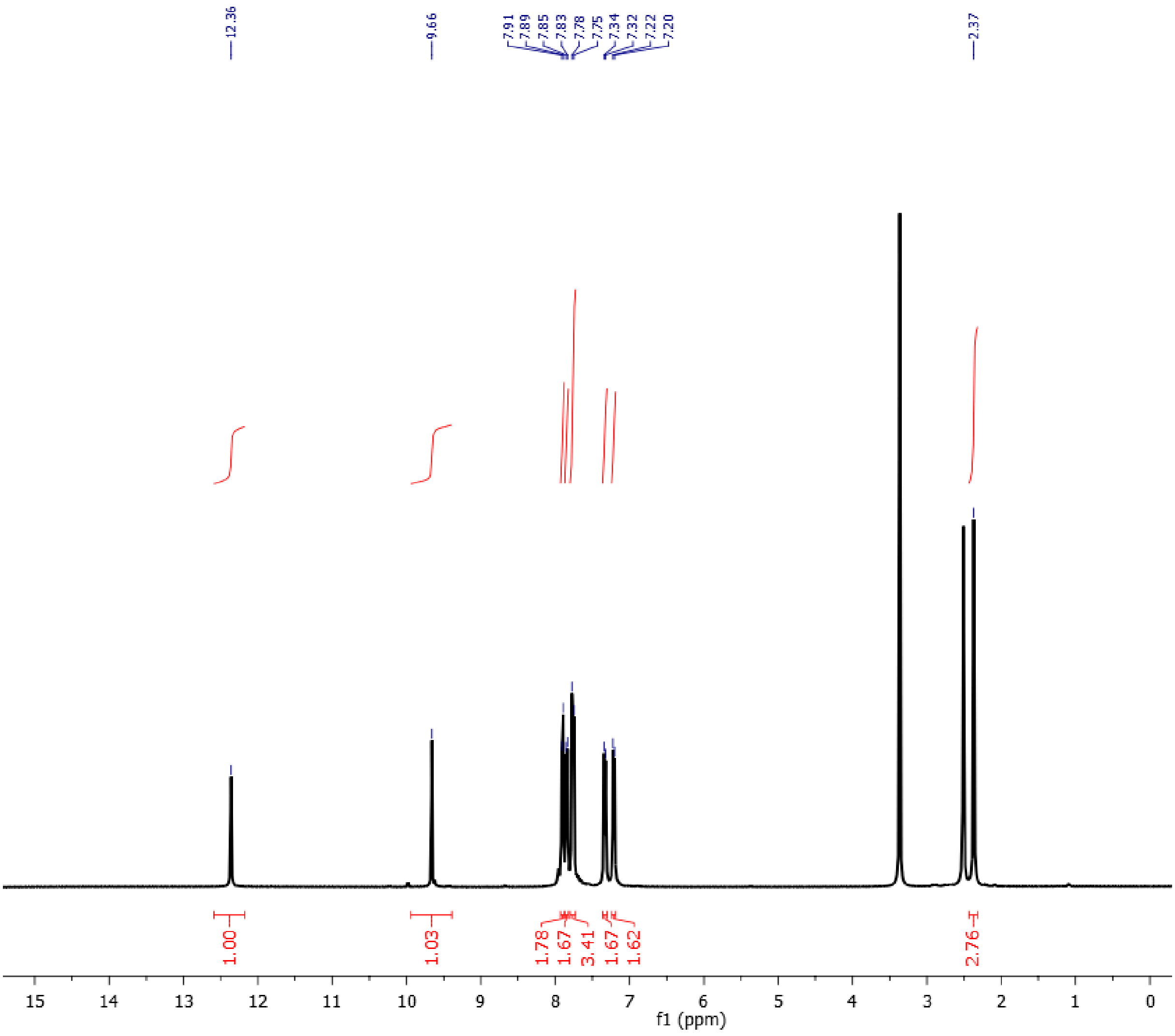

Figure S7. ^{1}H-NMR spectrum of compound 3c

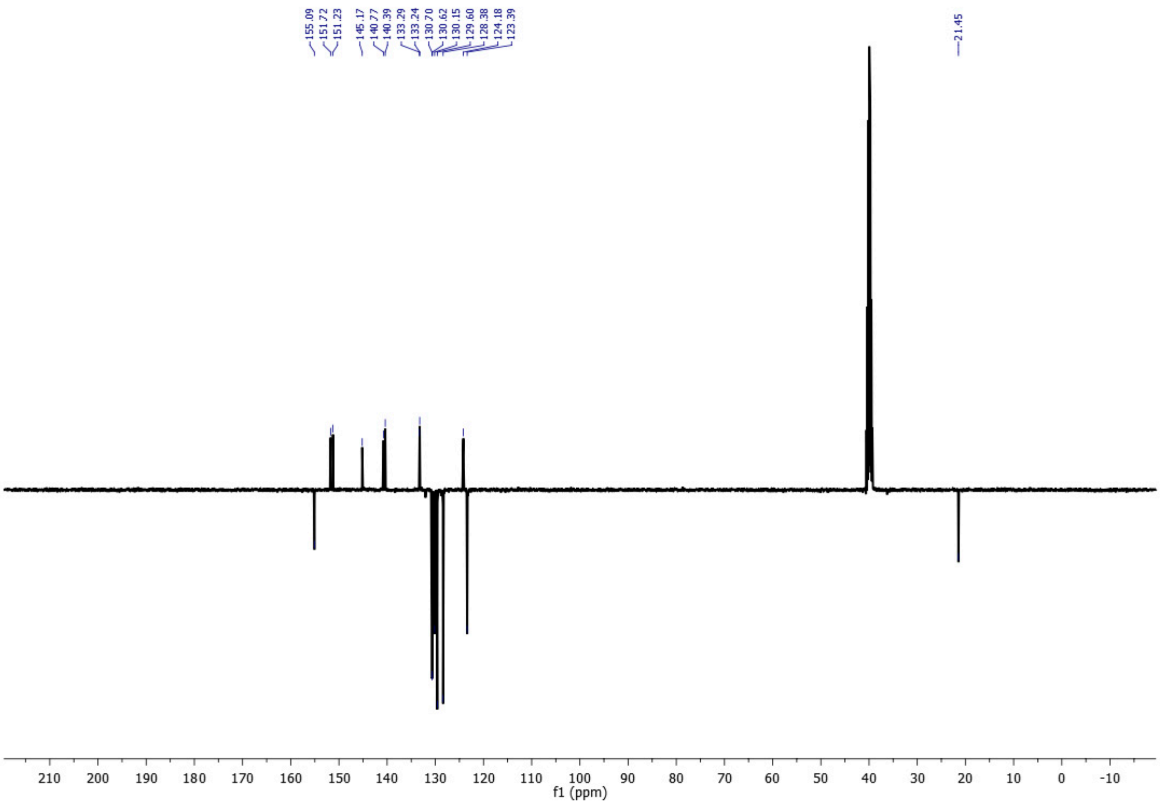

Figure S8. ^{13}C-NMR (APT) spectrum of compound 3c

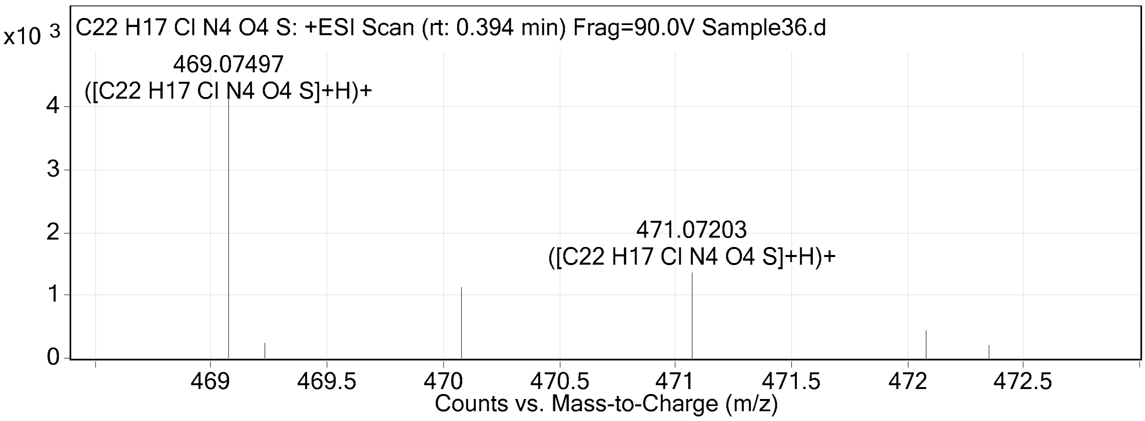

Figure S9. LC Q-TOF spectrum of compound 3c

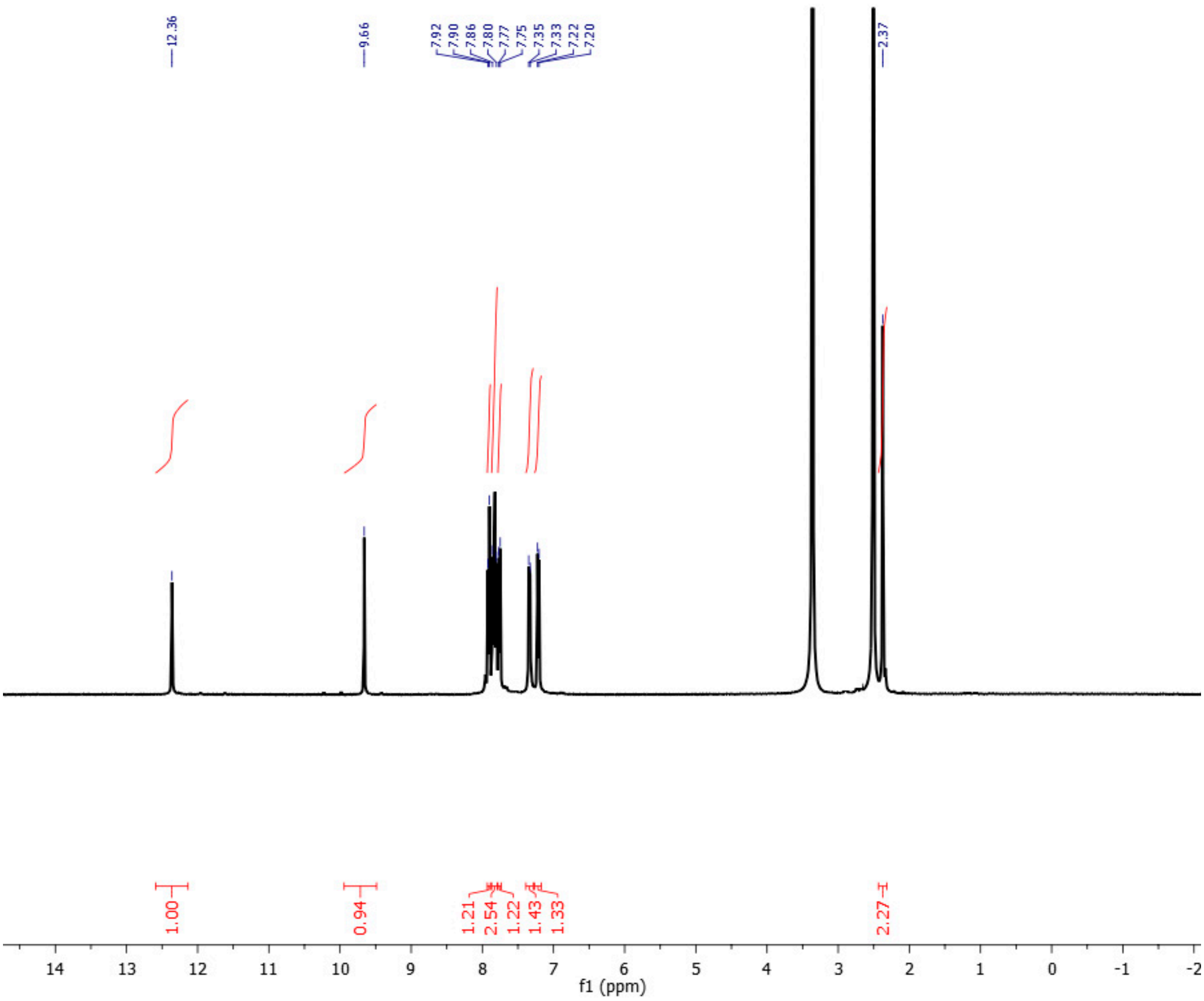

Figure S10. ^{1}H-NMR spectrum of compound 3d

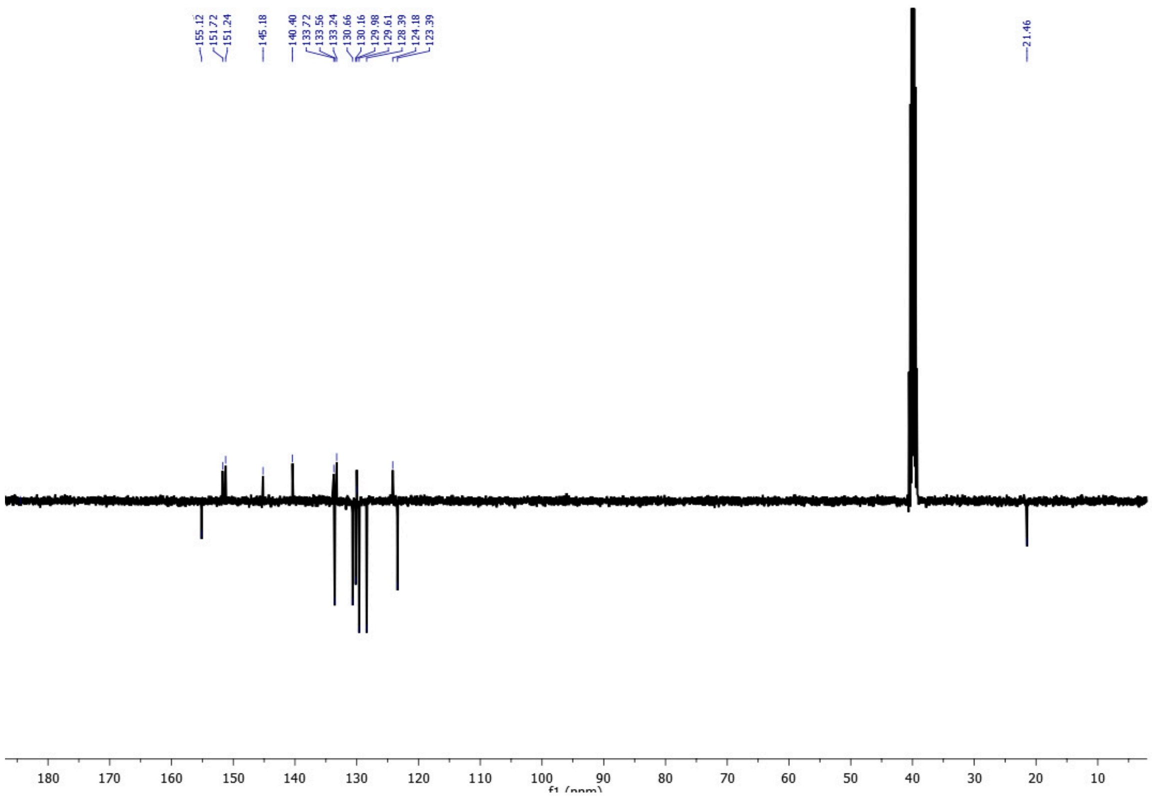

Figure S11. ^{13}C-NMR (APT) spectrum of compound 3d

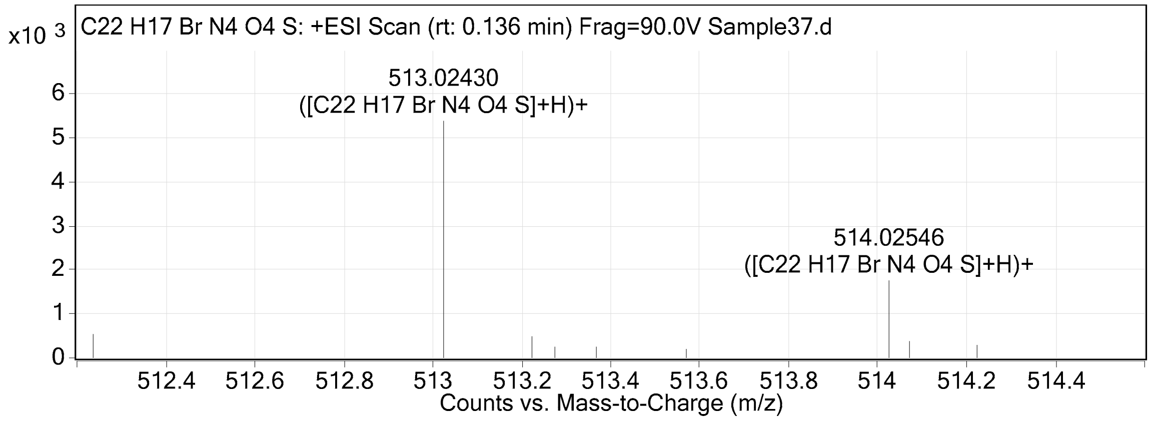

Figure S12. LC Q-TOF spectrum of compound 3d

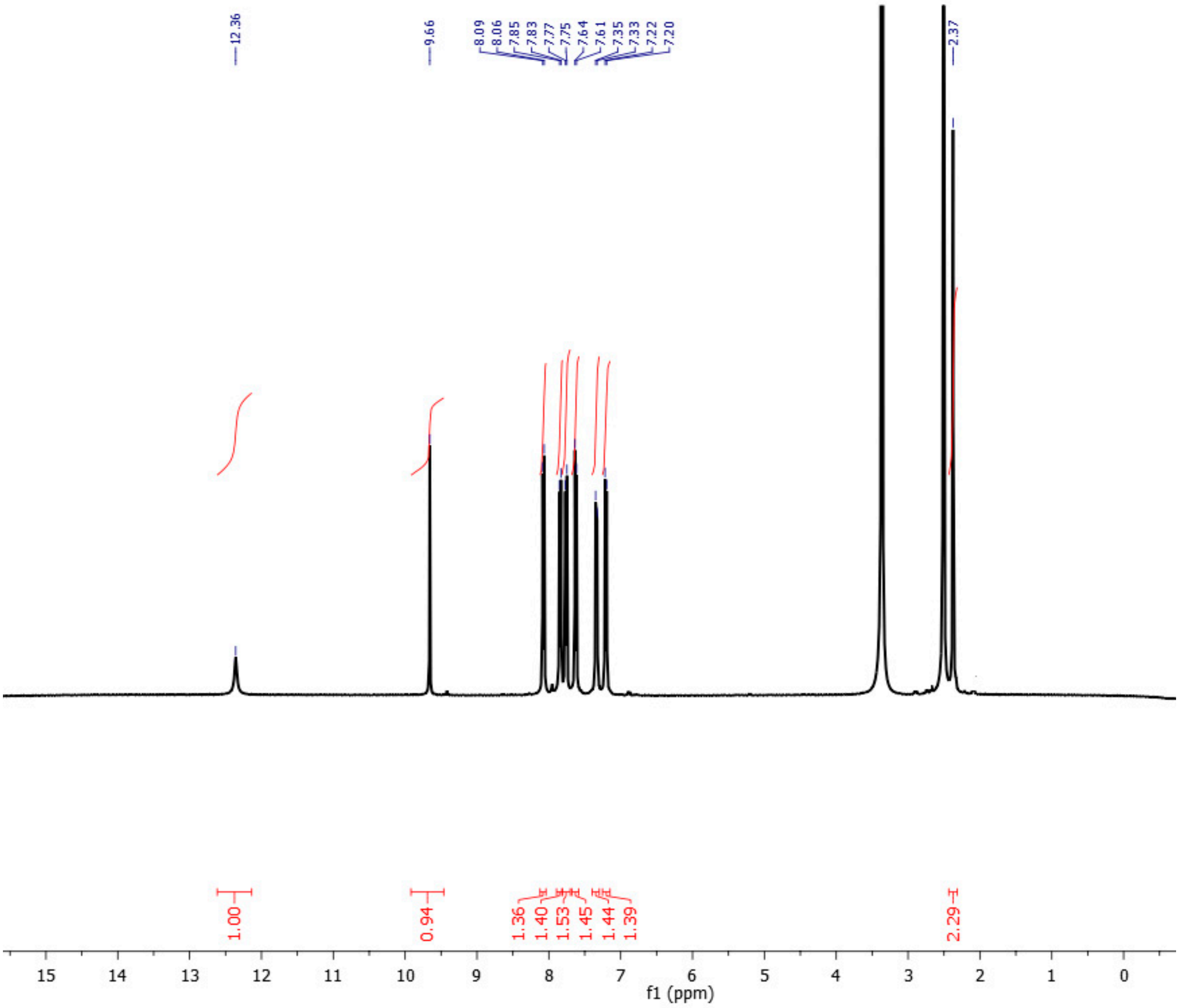

Figure S13. ^{1}H-NMR spectrum of compound 3e

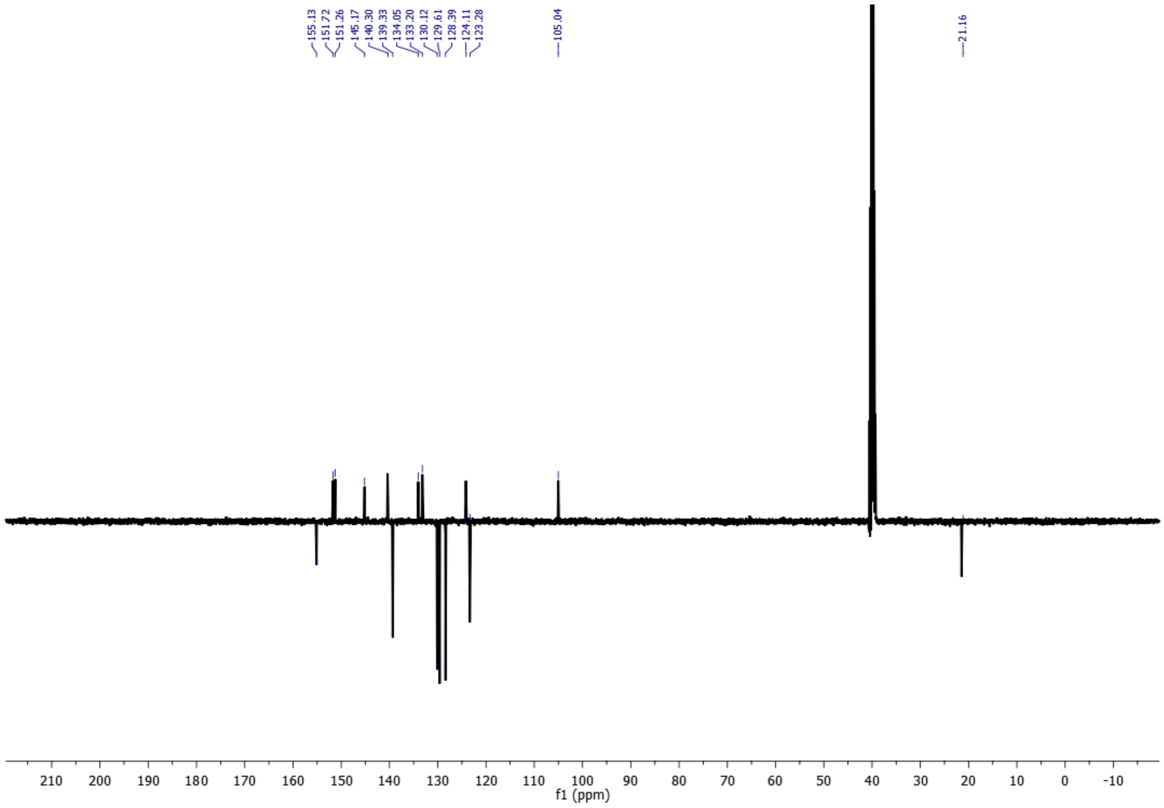

Figure S14. ^{13}C-NMR (APT) spectrum of compound 3e

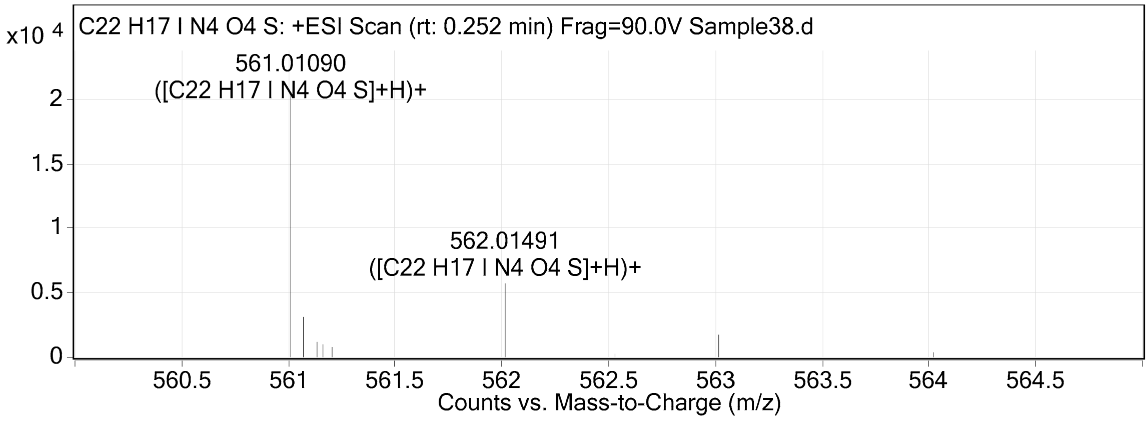

Figure S15. LC Q-TOF spectrum of compound 3e

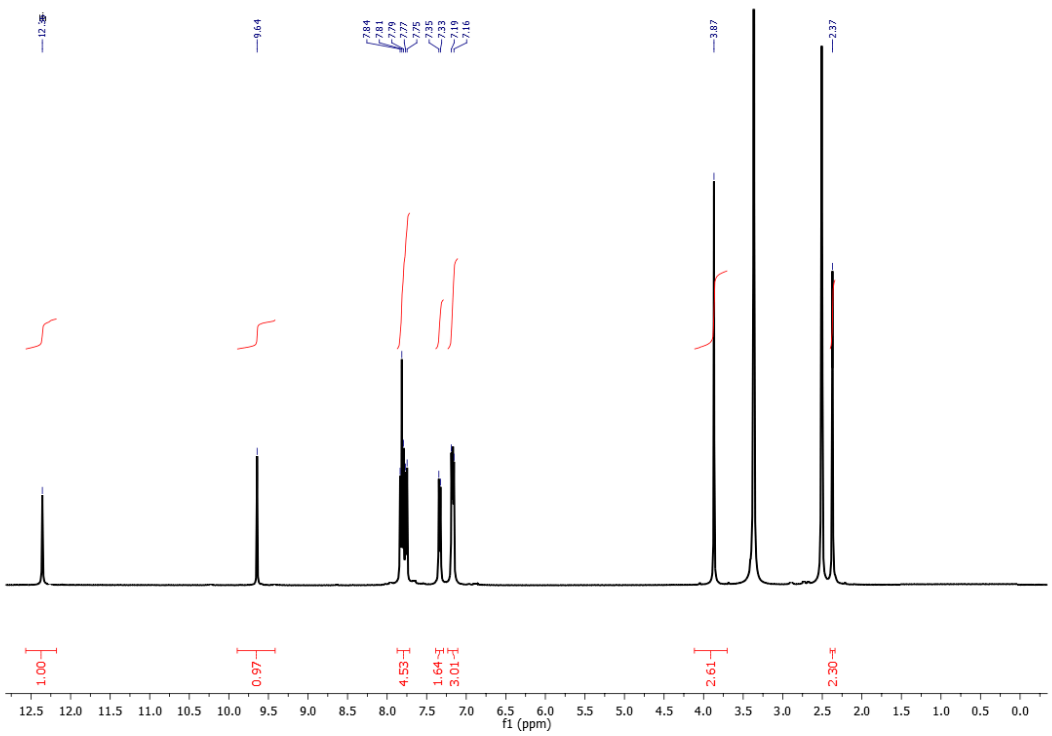

Figure S16. ^{1}H-NMR spectrum of compound 3f

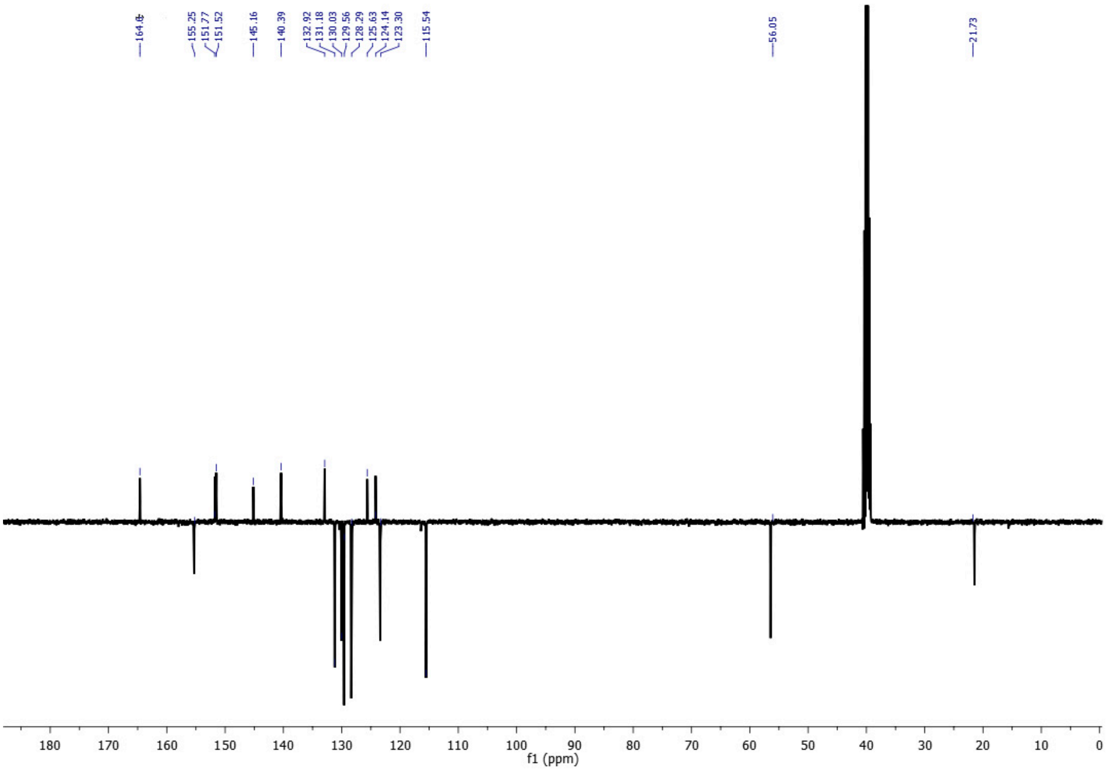

Figure S17. ^{13}C-NMR (APT) spectrum of compound 3f

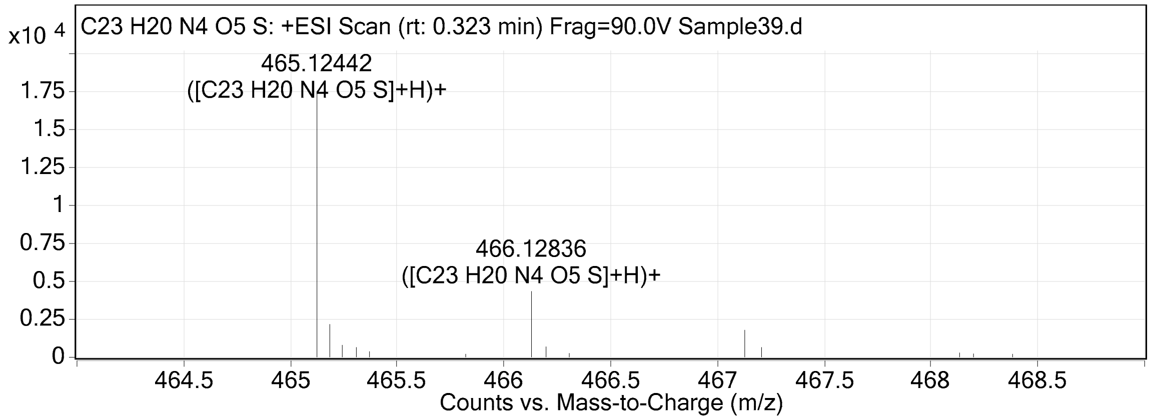

Figure S18. LC Q-TOF spectrum of compound 3f

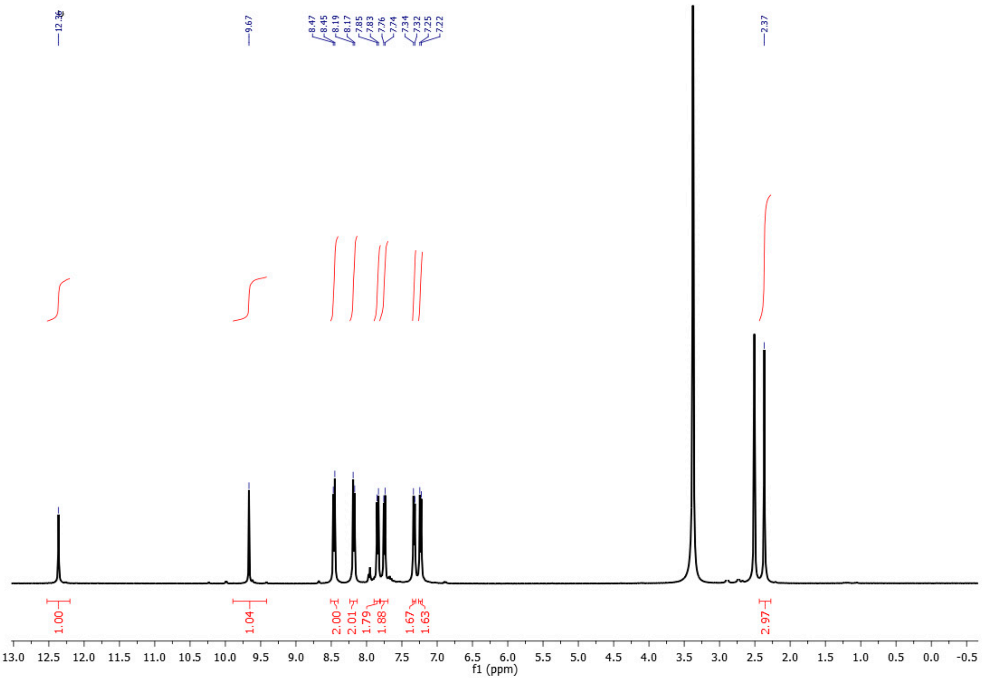

Figure S19. ^{1}H-NMR spectrum of compound 3g

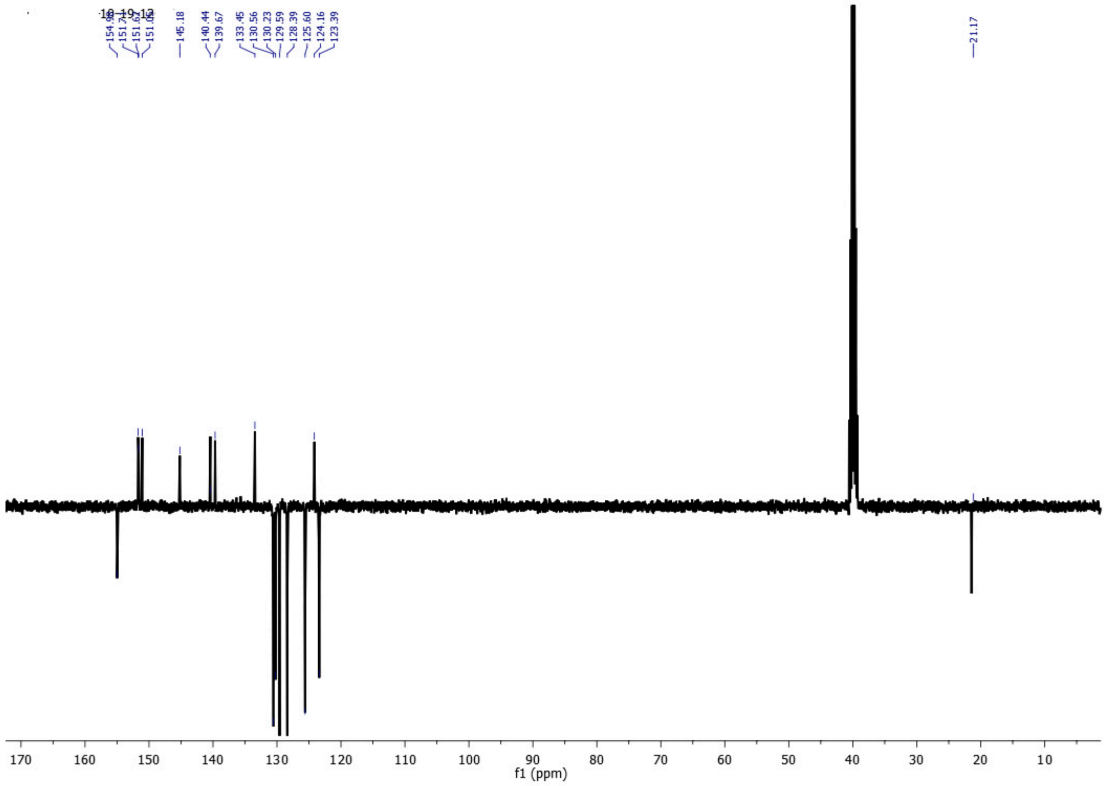

Figure S20. ^{13}C-NMR (APT) spectrum of compound 3g

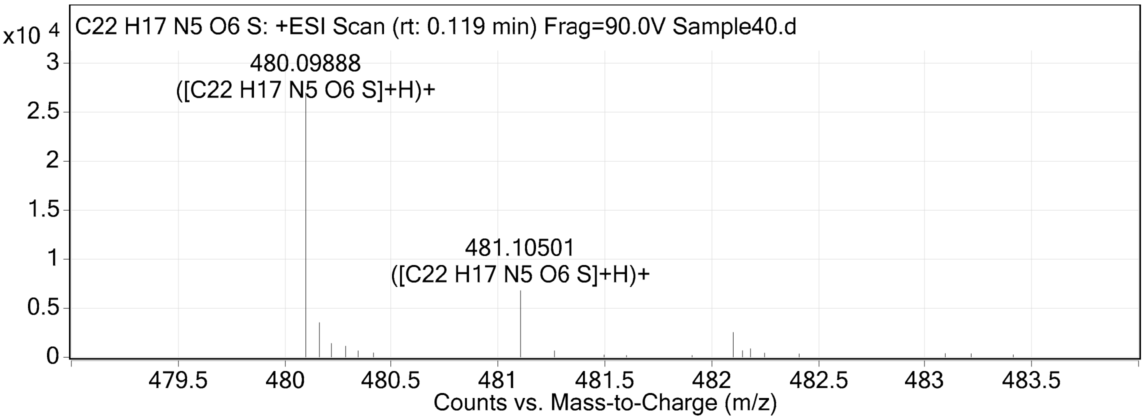

Figure S21. LC Q-TOF spectrum of compound 3g

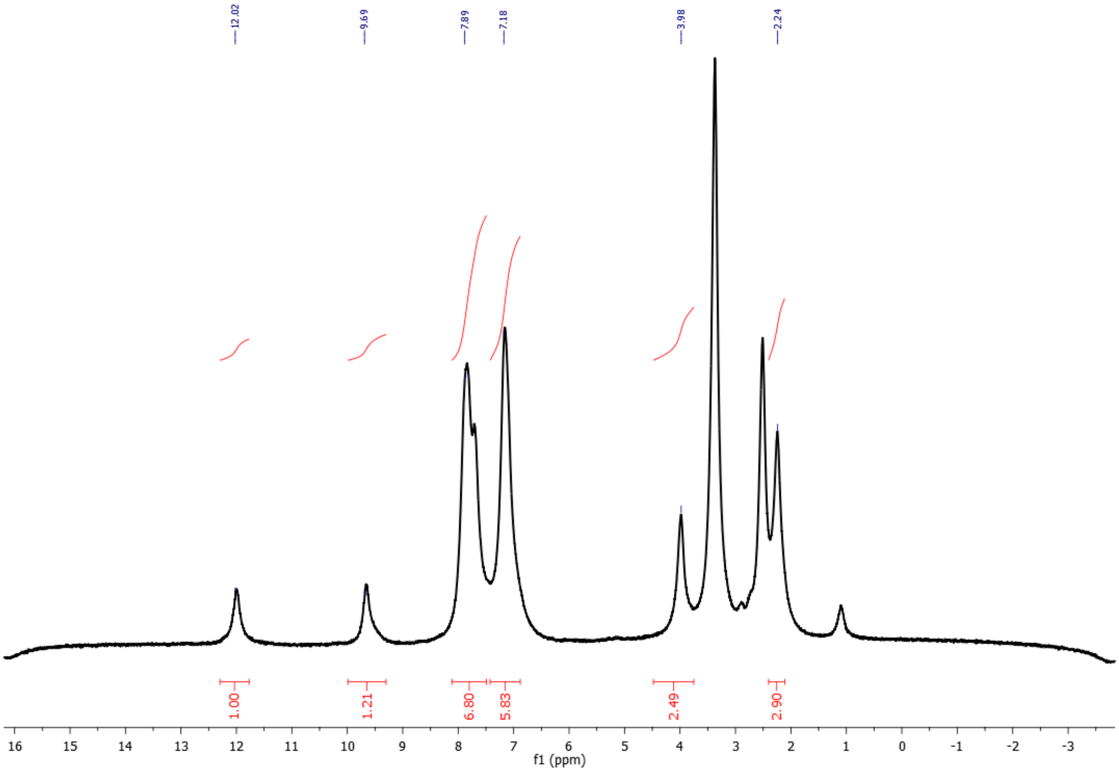

Figure S22. ^{1}H-NMR spectrum of compound 4a

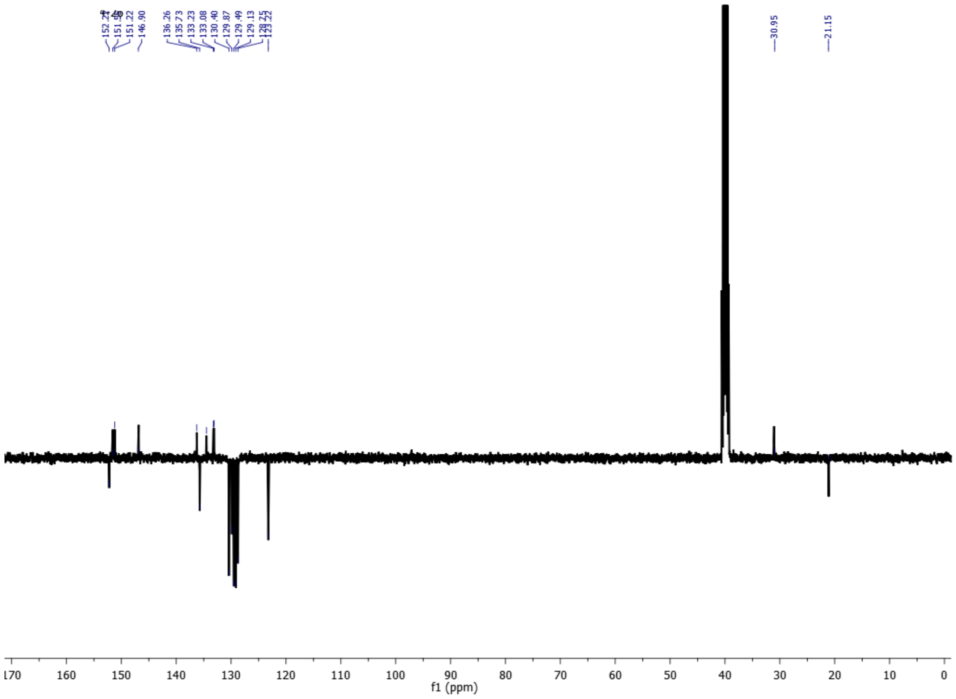

Figure S23. ^{13}C-NMR (APT) spectrum of compound 4a

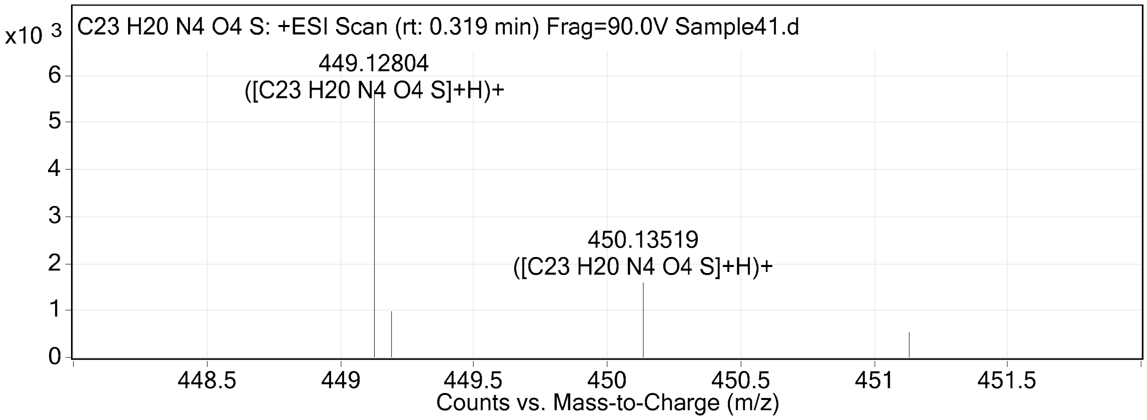

Figure S24. LC Q-TOF spectrum of compound 4a

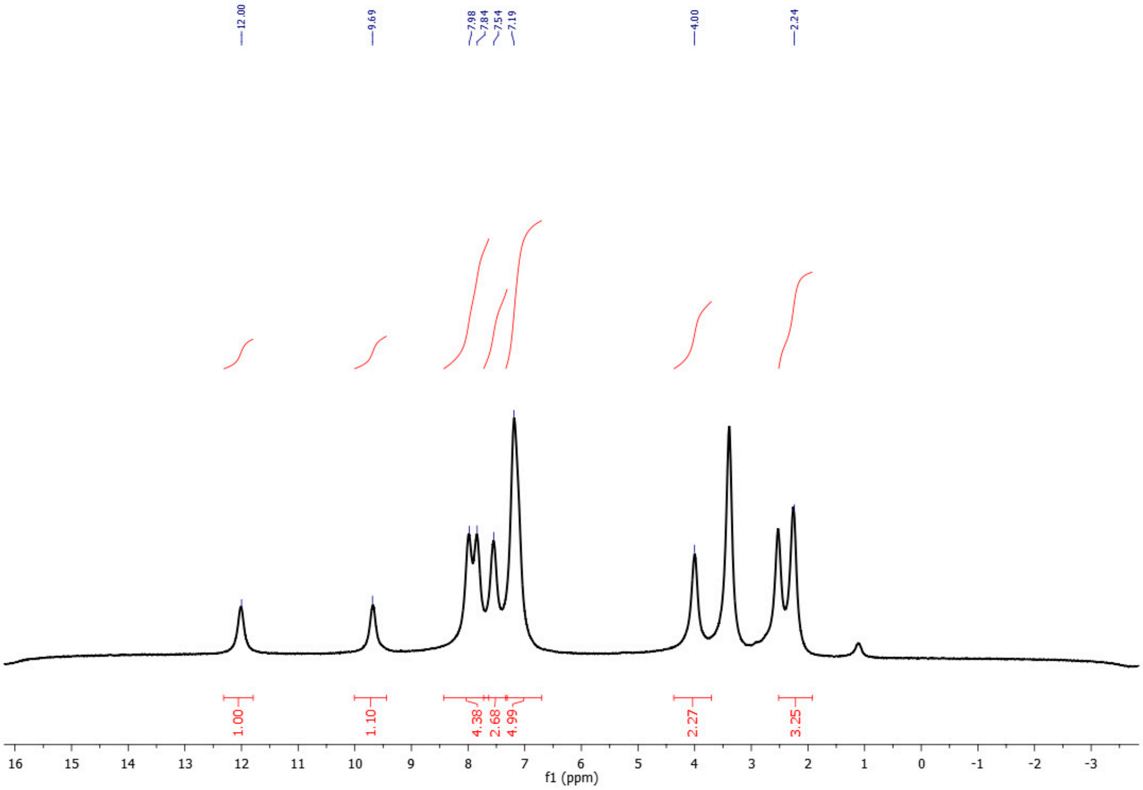

Figure S25. ^{1}H-NMR spectrum of compound 4b

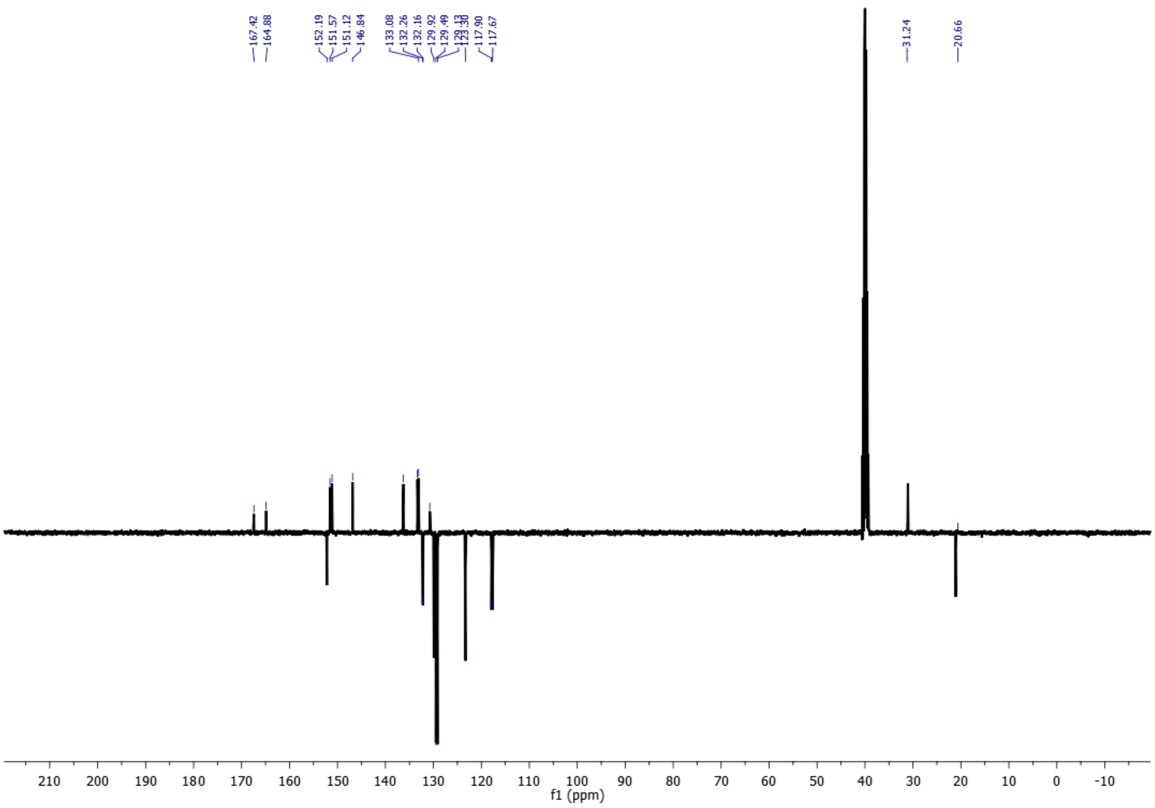

Figure S26. ^{13}C-NMR (APT) spectrum of compound 4b

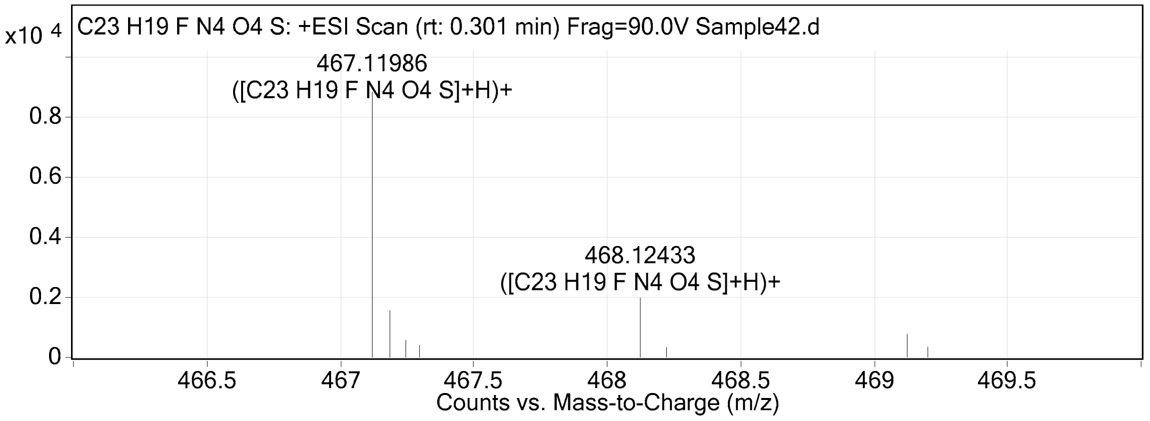

Figure S27. LC Q-TOF spectrum of compound 4b

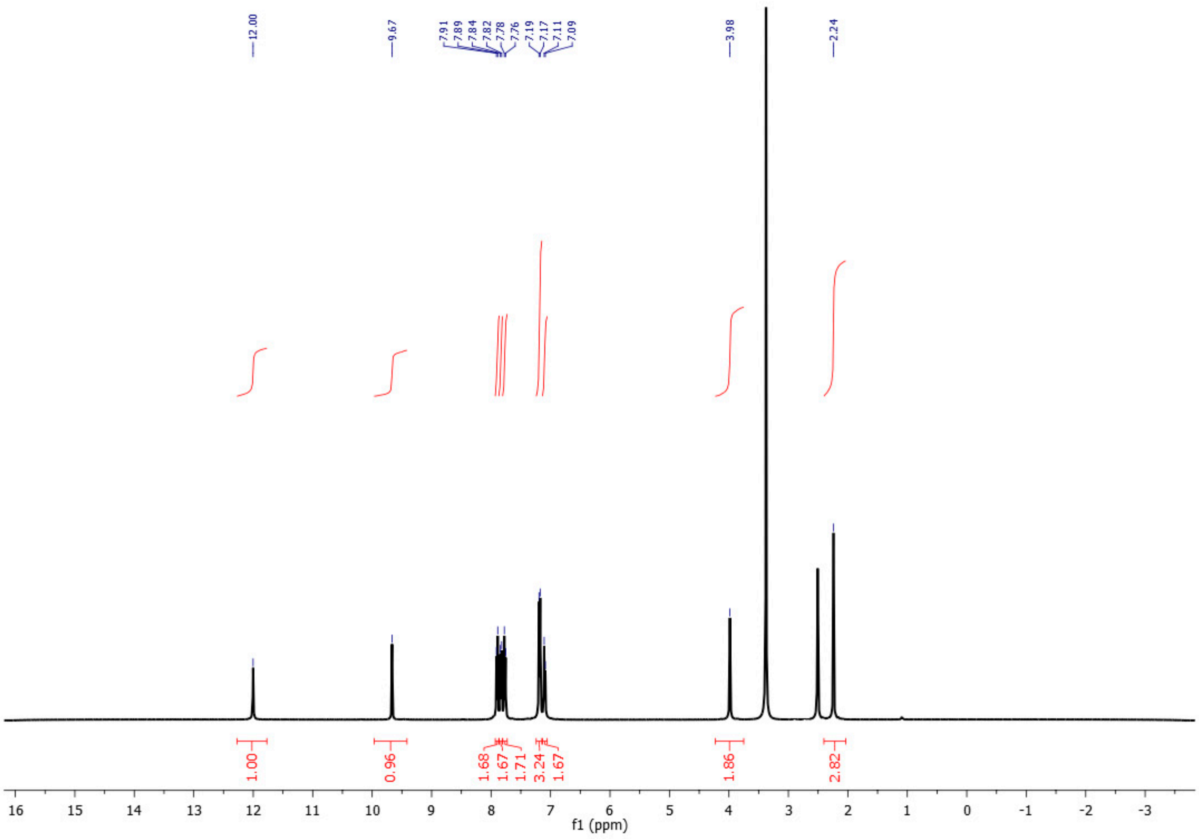

Figure S28. ^{1}H-NMR spectrum of compound 4c

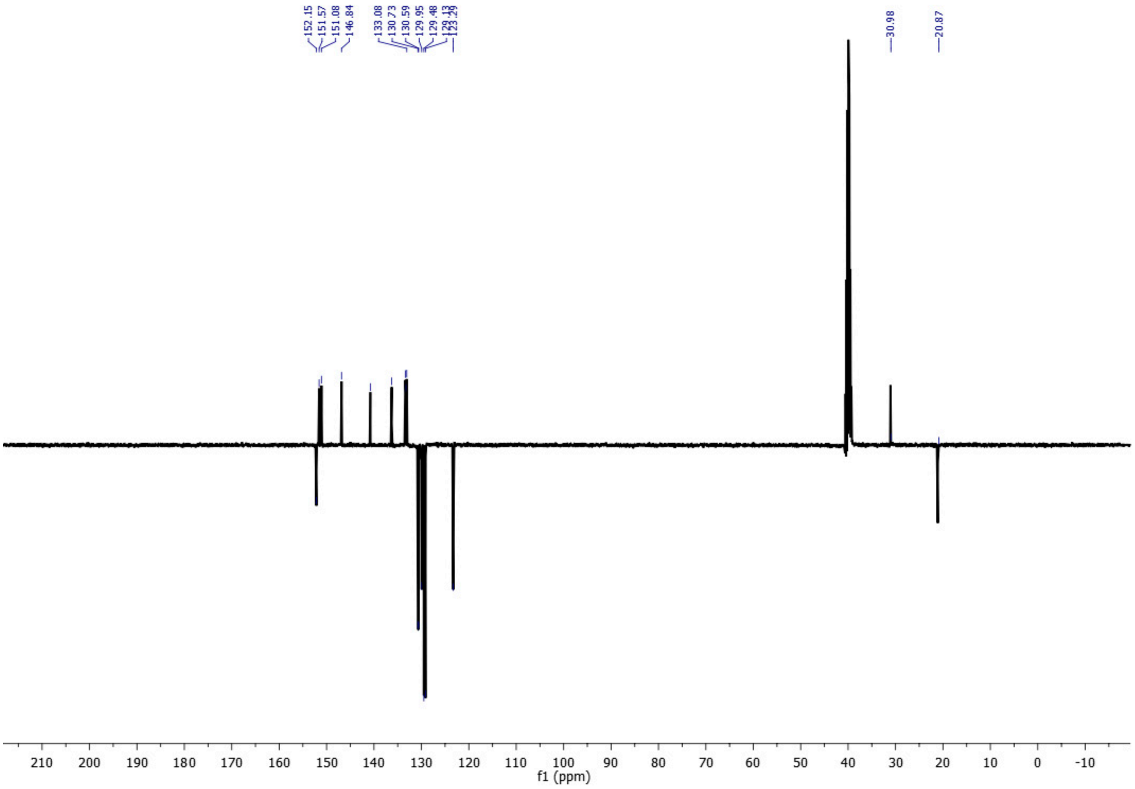

Figure S29. ^{13}C-NMR (APT) spectrum of compound 4c

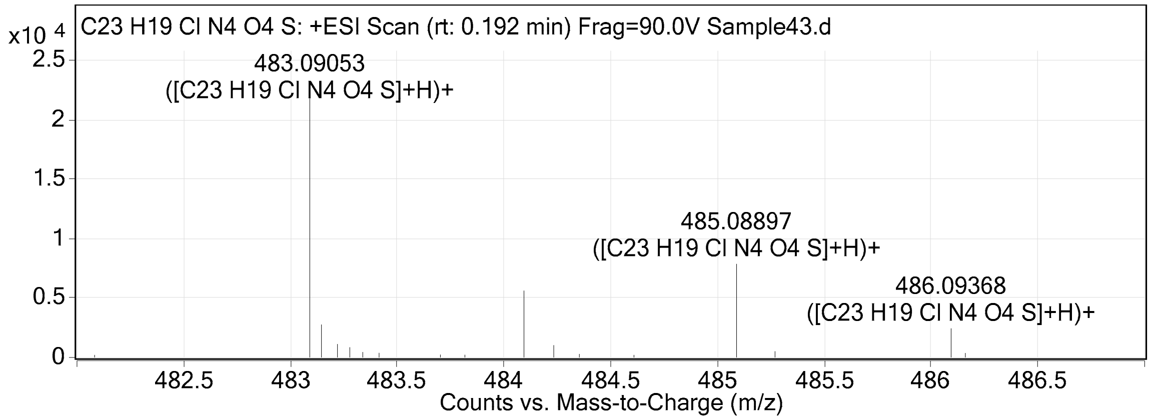

Figure S30. LC Q-TOF spectrum of compound 4c

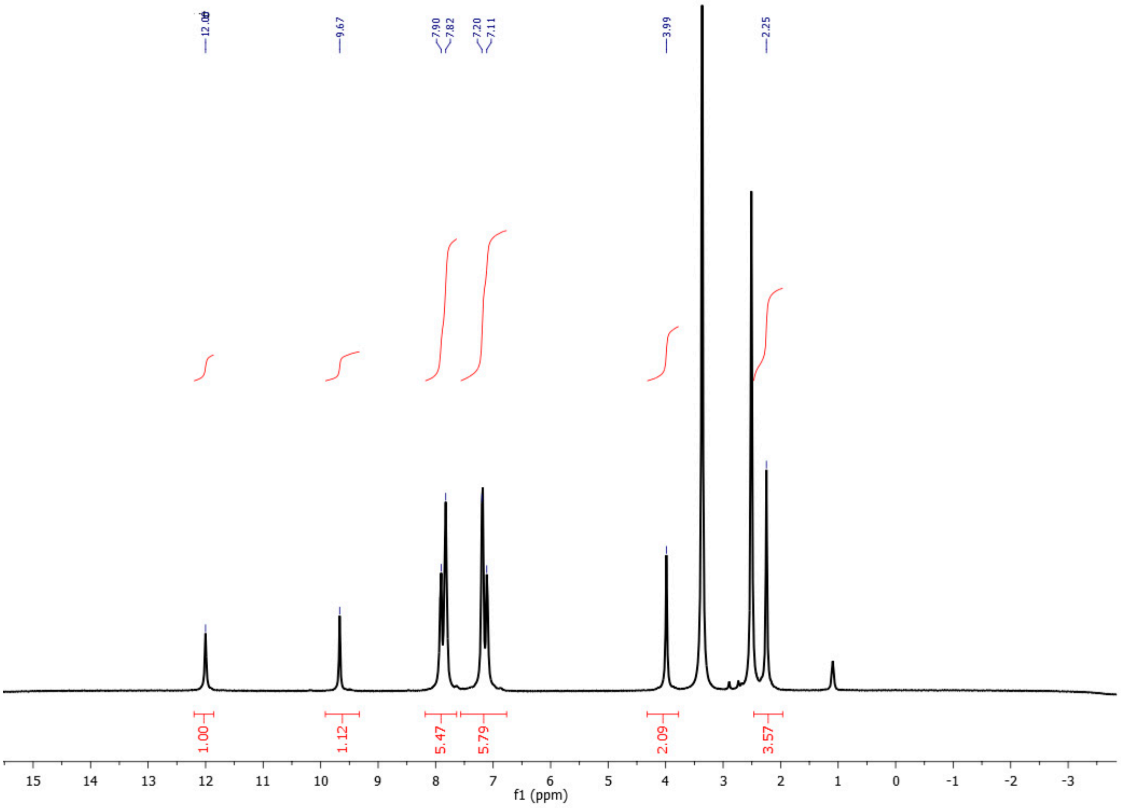

Figure S31. ^{1}H-NMR spectrum of compound 4d

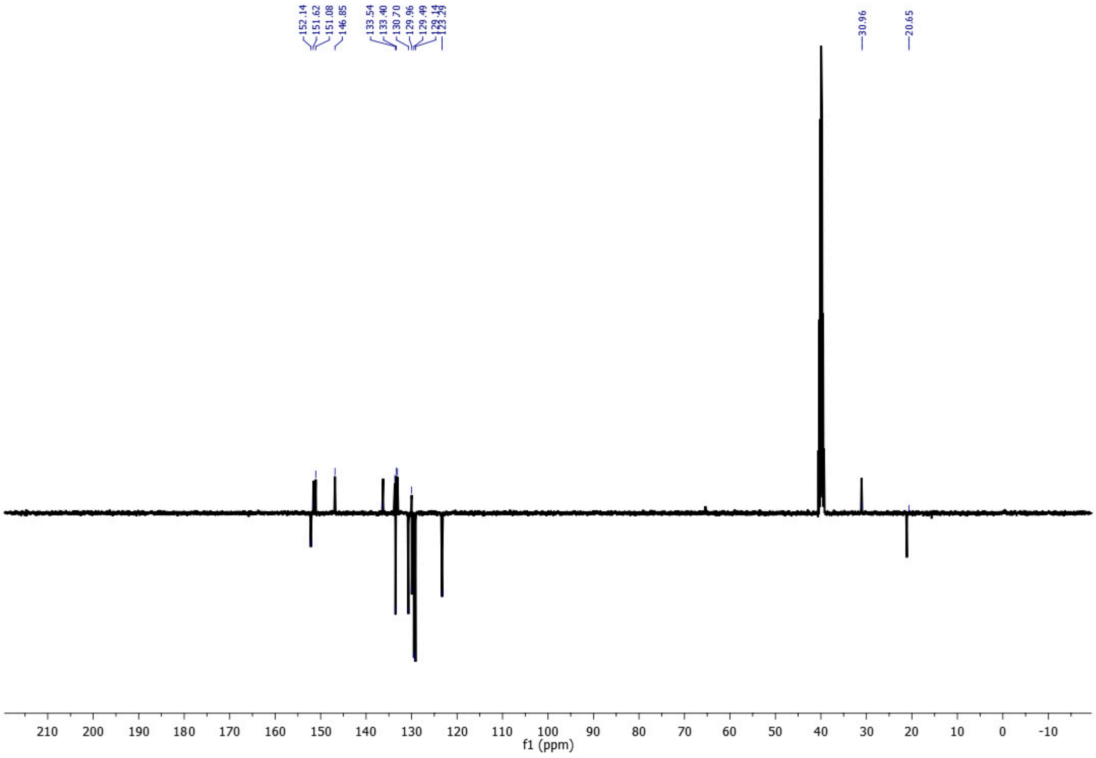

Figure S32. ^{13}C-NMR (APT) spectrum of compound 4d

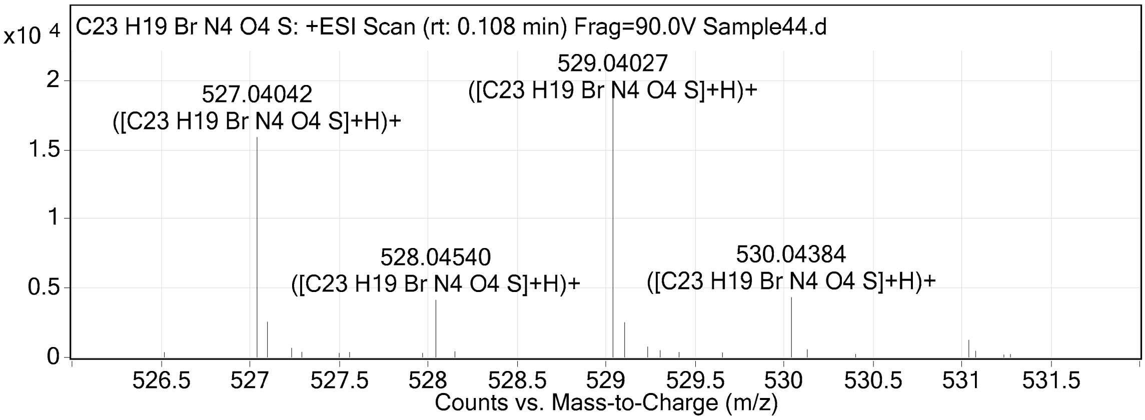

Figure S33. LC Q-TOF spectrum of compound 4d

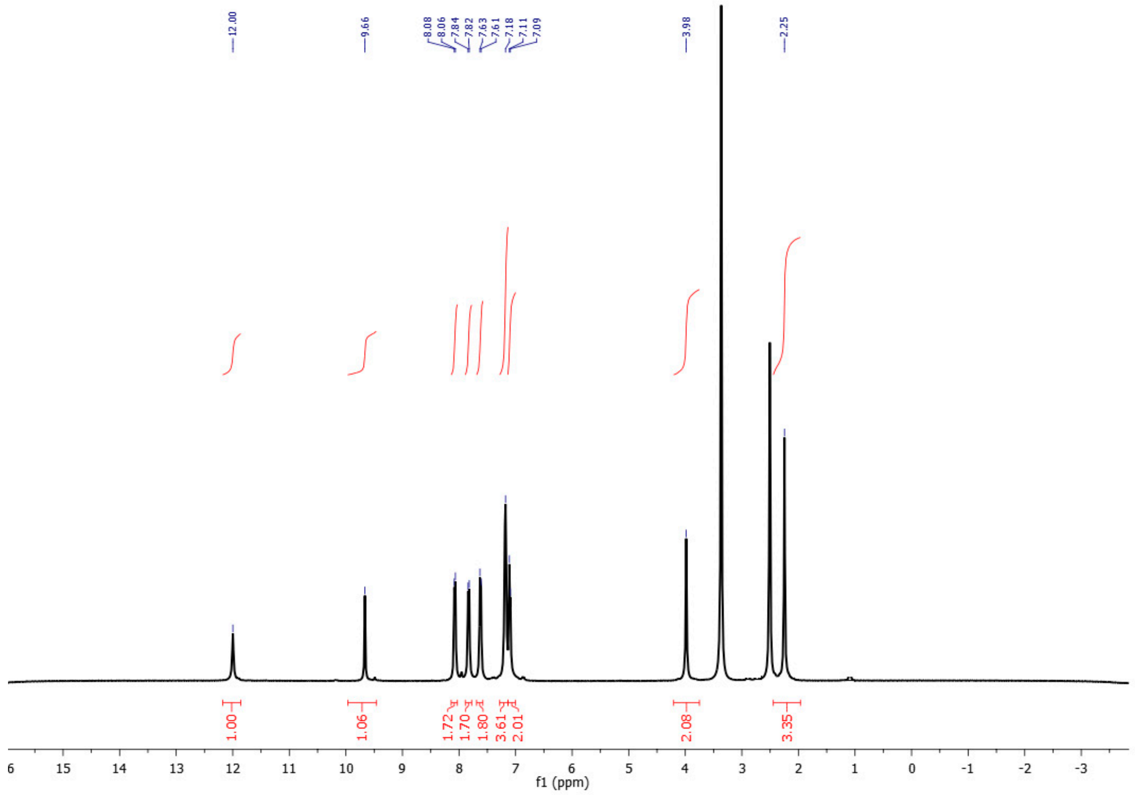

Figure S34. ^{1}H-NMR spectrum of compound 4e

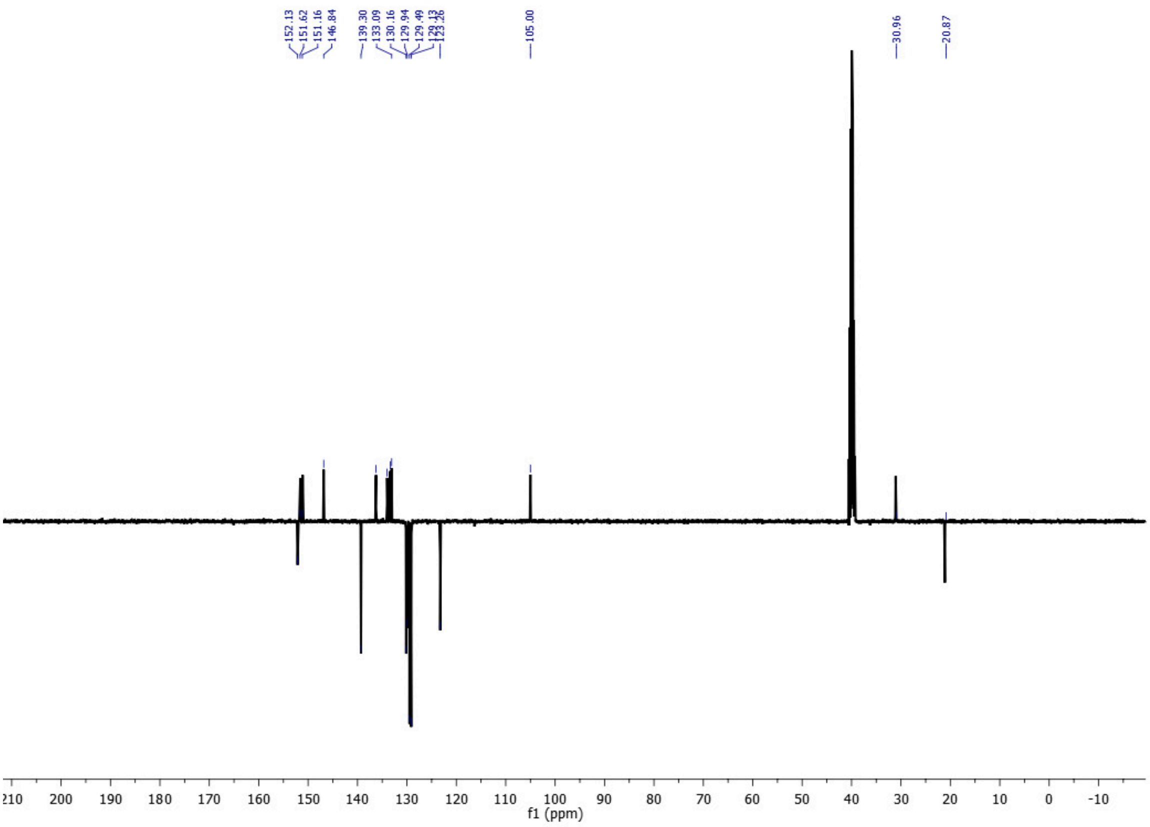

Figure S35. ^{13}C-NMR (APT) spectrum of compound 4e

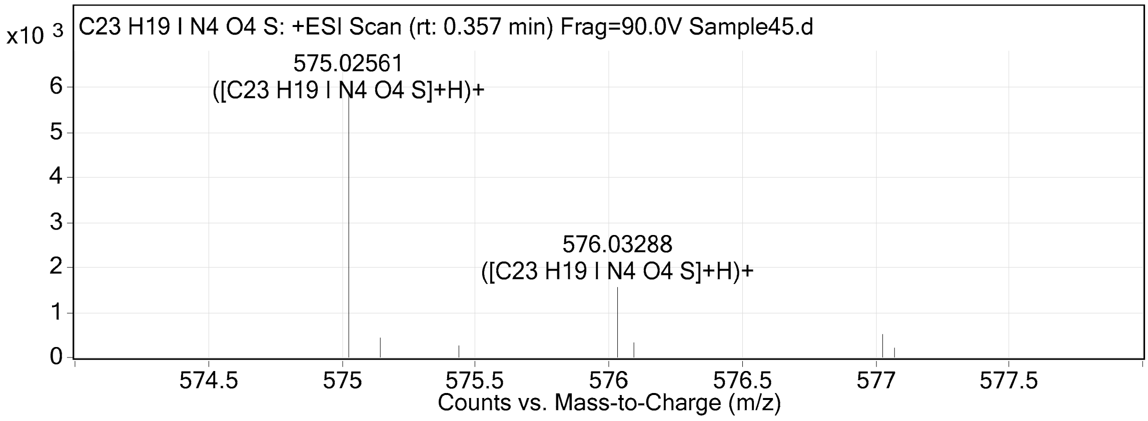

Figure S36. LC Q-TOF spectrum of compound 4e

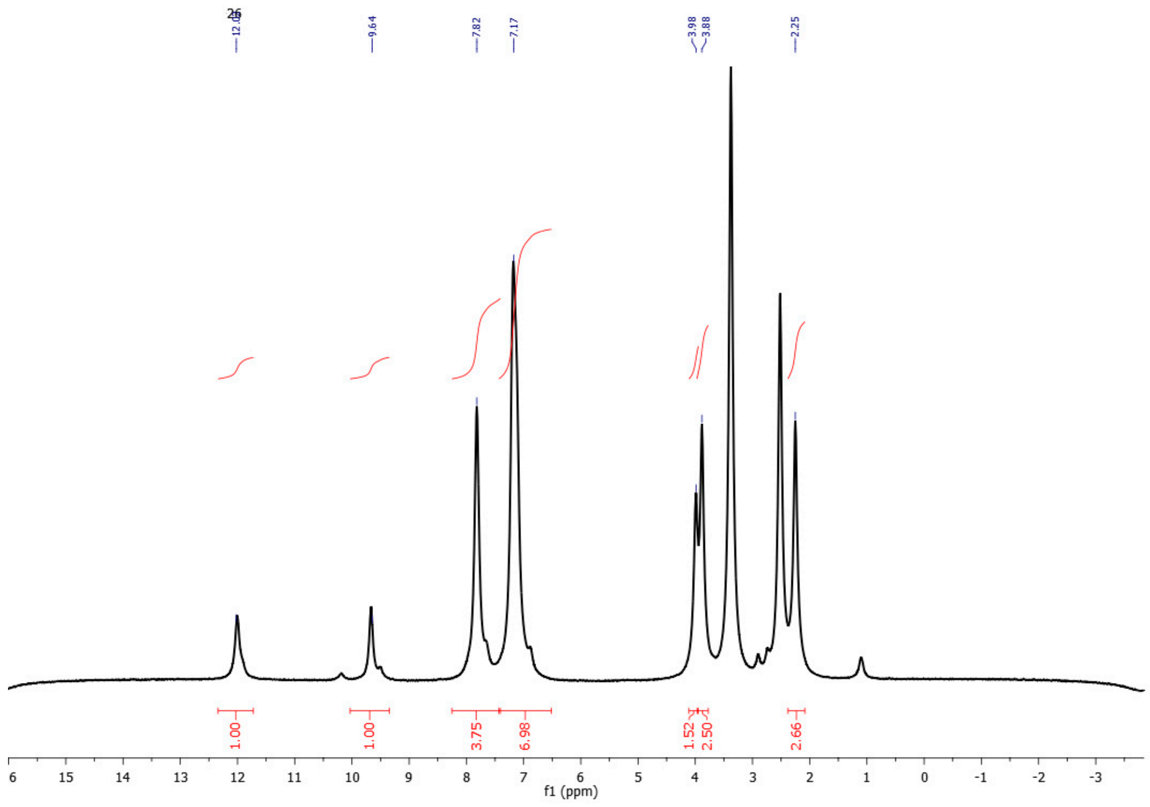

Figure S37. ^{1}H-NMR spectrum of compound 4f

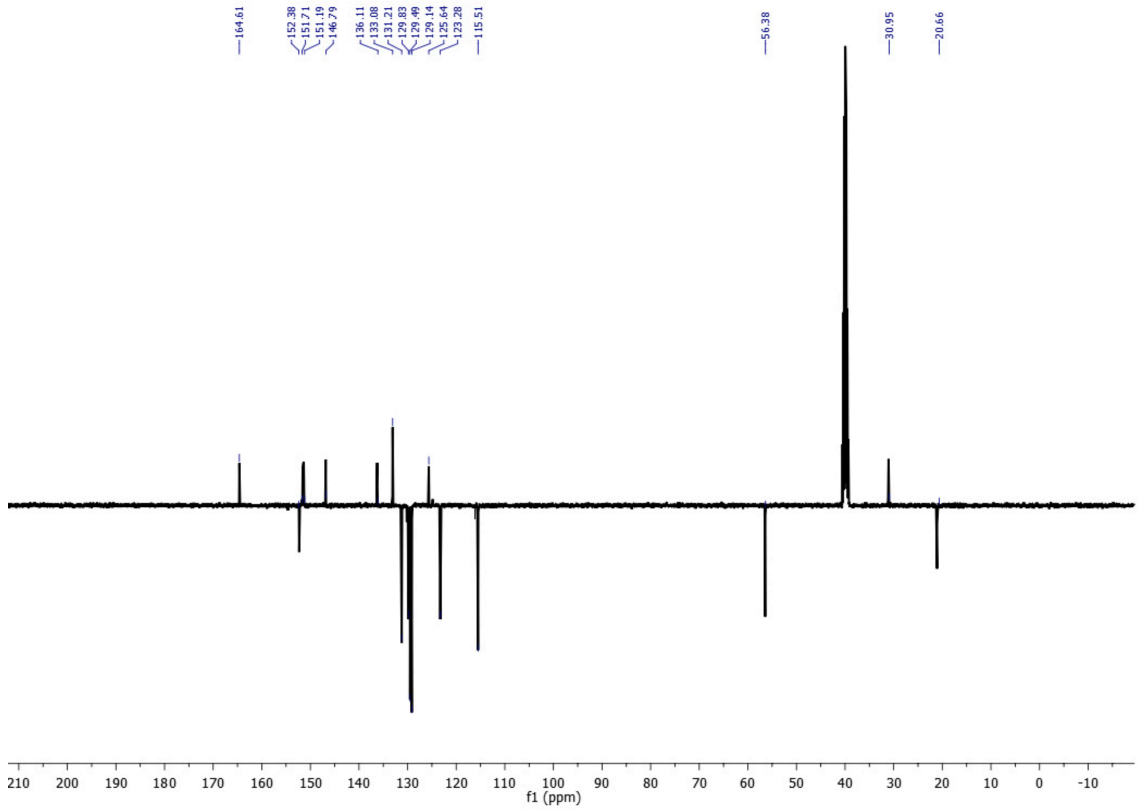

Figure S38. ^{13}C-NMR (APT) spectrum of compound 4f

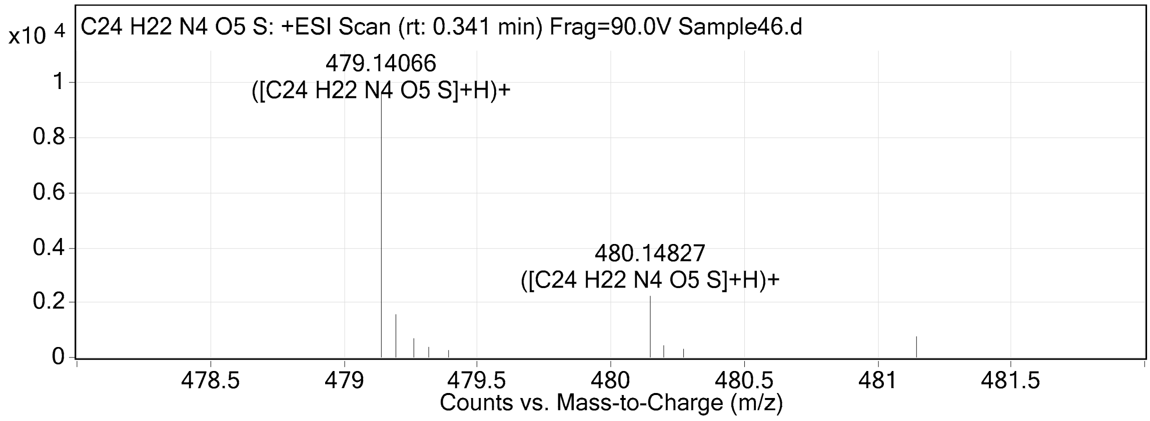

Figure S39. LC Q-TOF spectrum of compound 4f

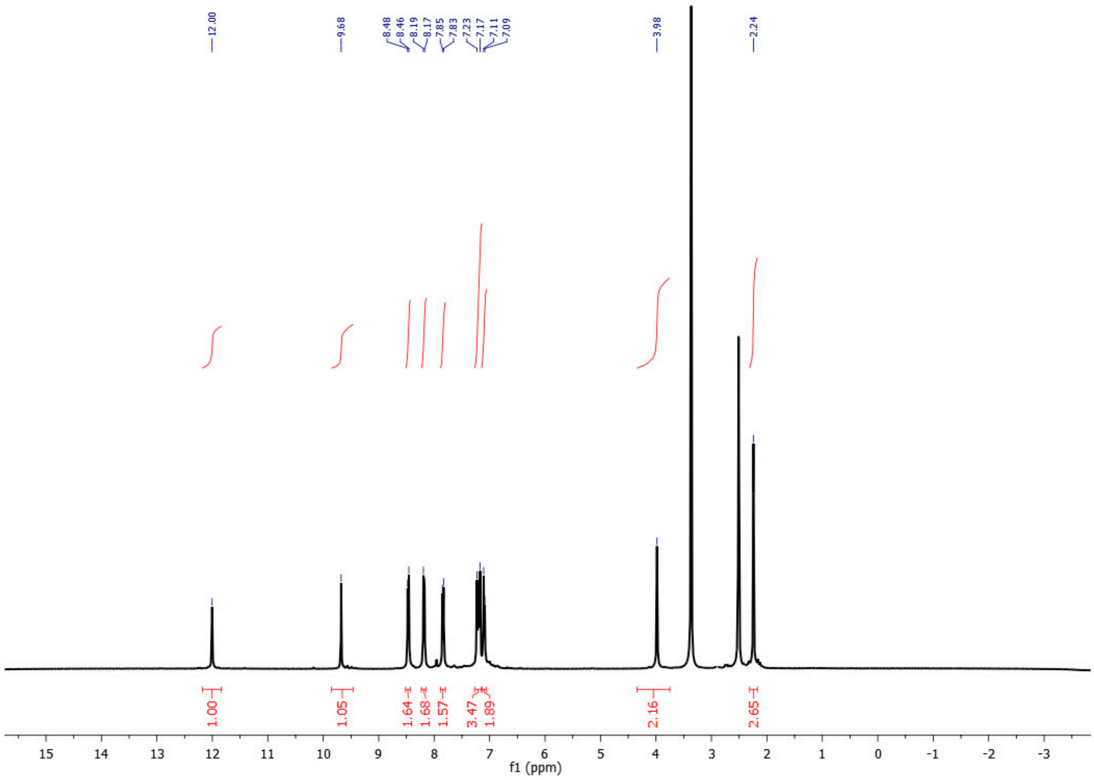

Figure S40. ^{1}H-NMR spectrum of compound 4g

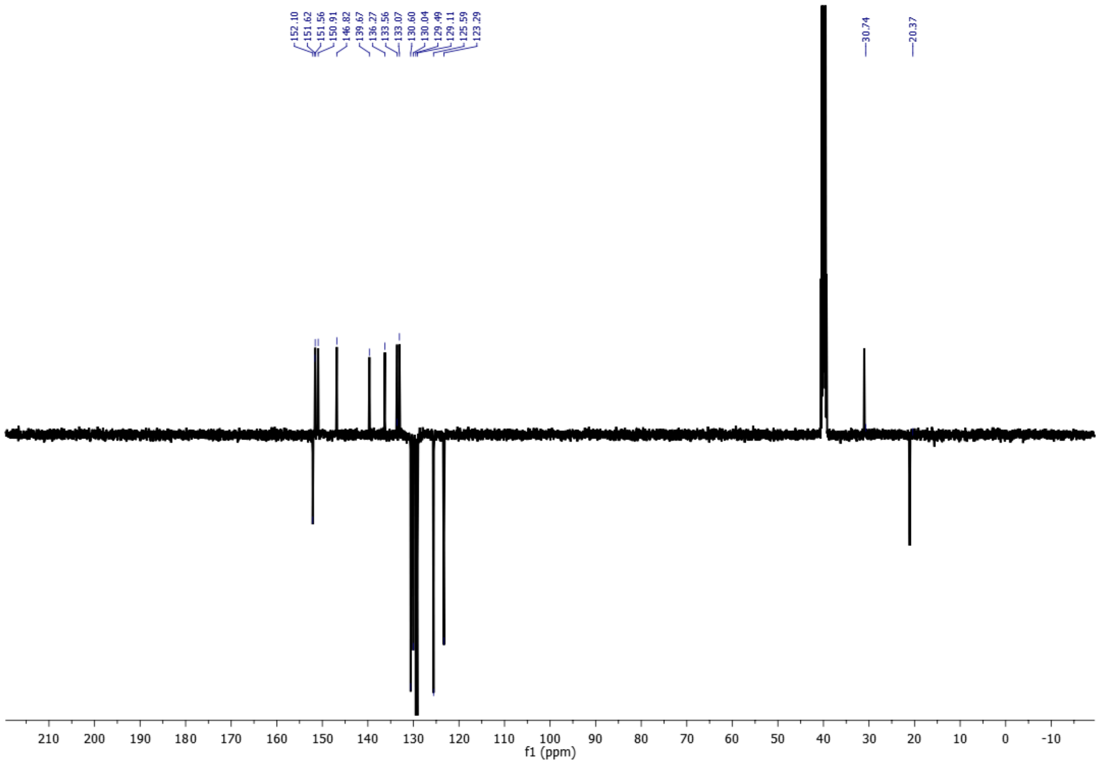

Figure S41. ^{13}C-NMR (APT) spectrum of compound 4g

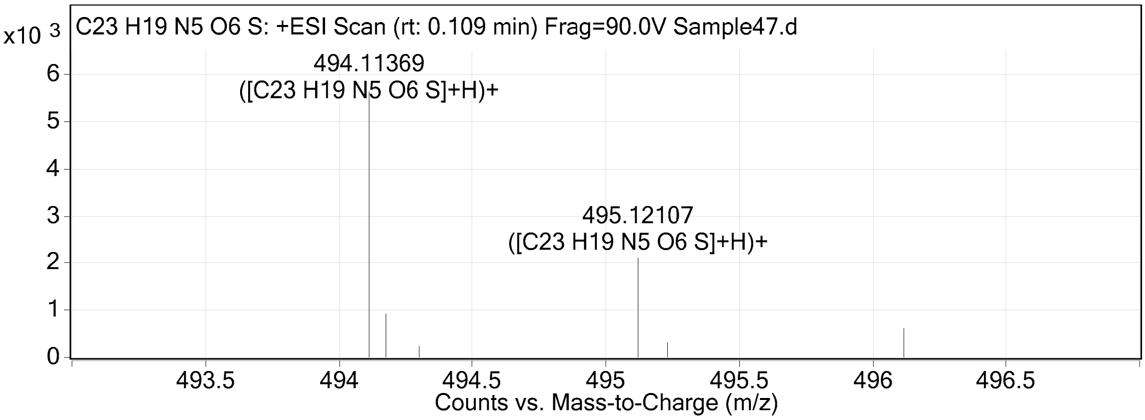

Figure S42. LC Q-TOF spectrum of compound 4g

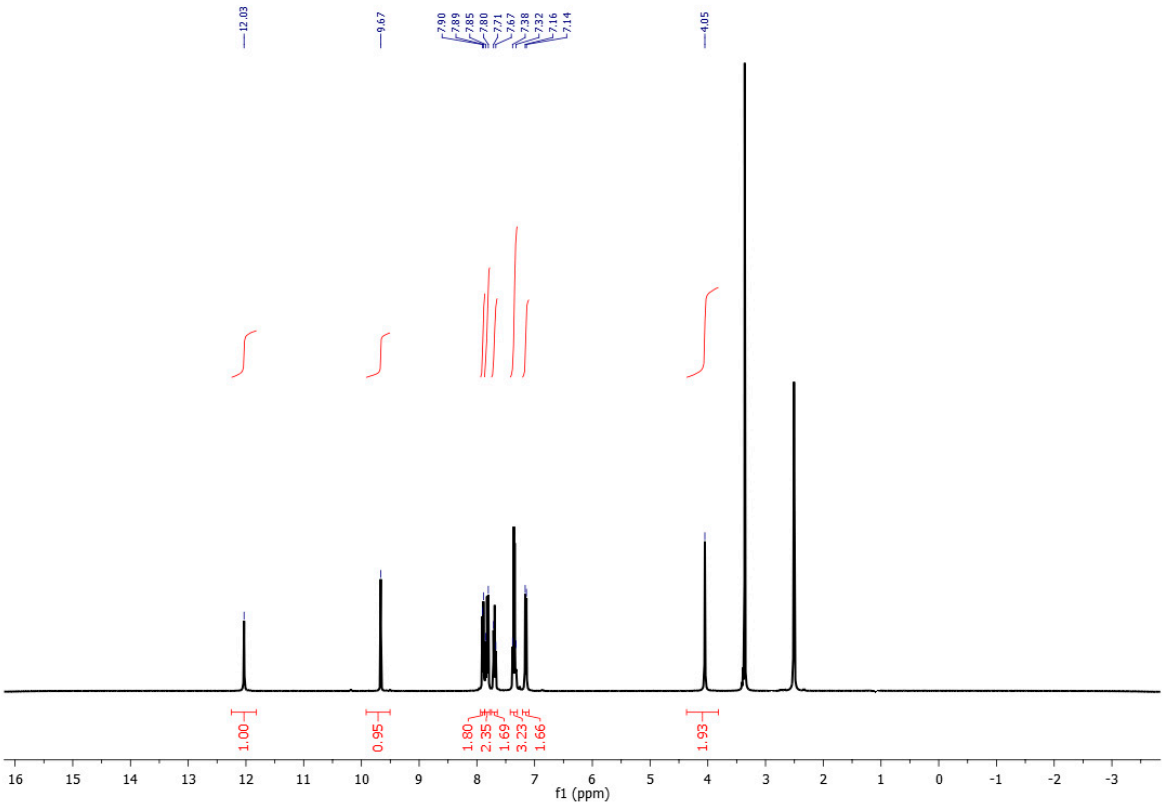

Figure S43. ^{1}H-NMR spectrum of compound 5a

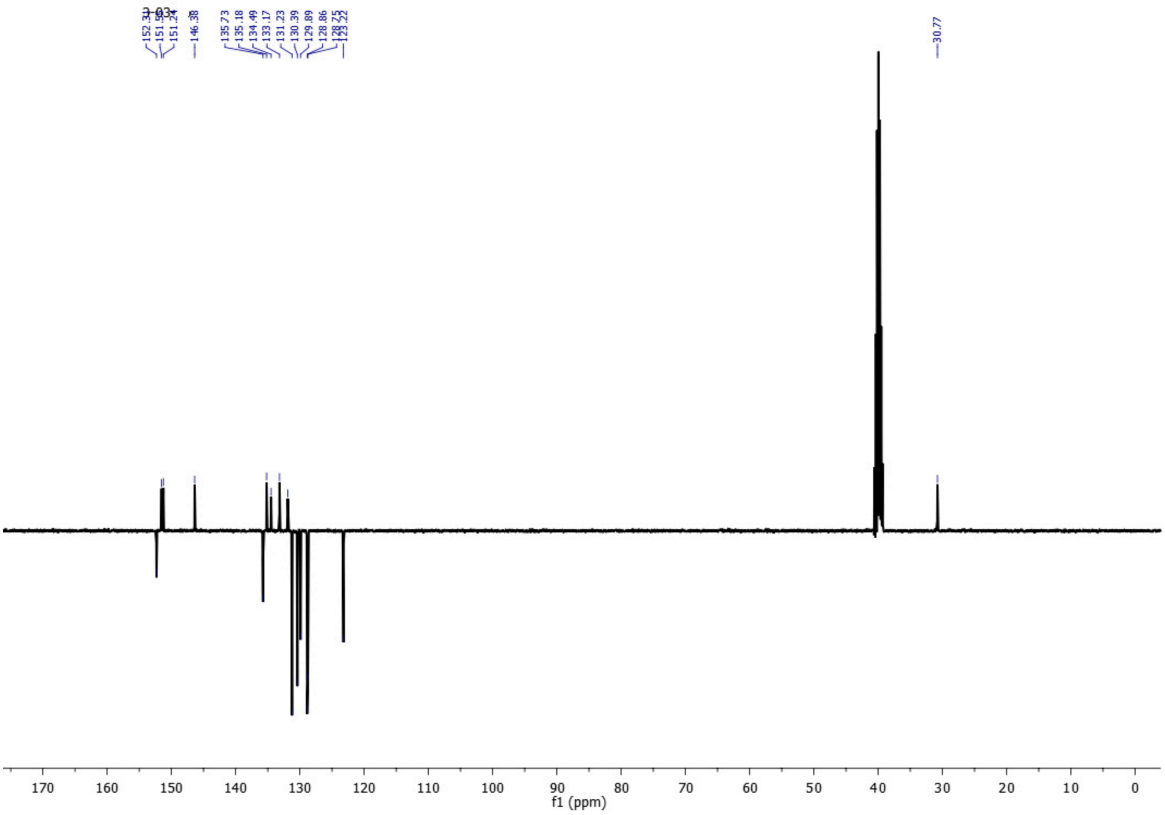

Figure S44. ^{13}C-NMR (APT) spectrum of compound 5a

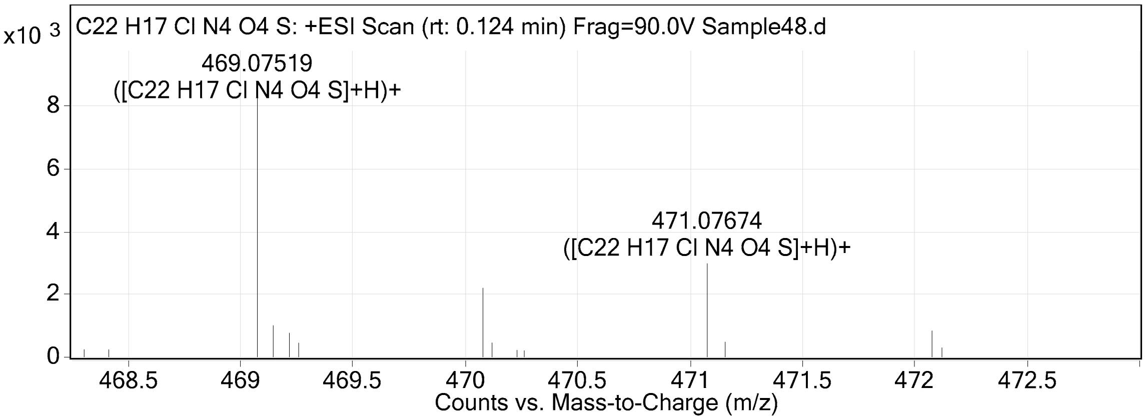

Figure S45. LC Q-TOF spectrum of compound 5a

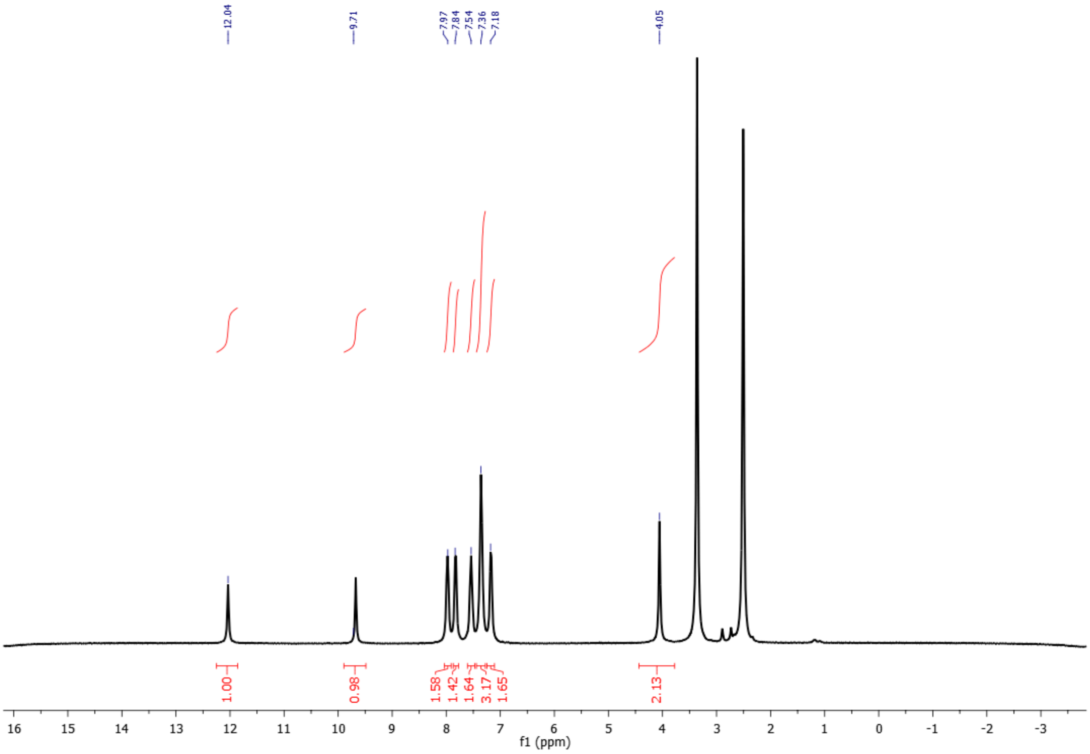

Figure S46. ^{1}H-NMR spectrum of compound 5b

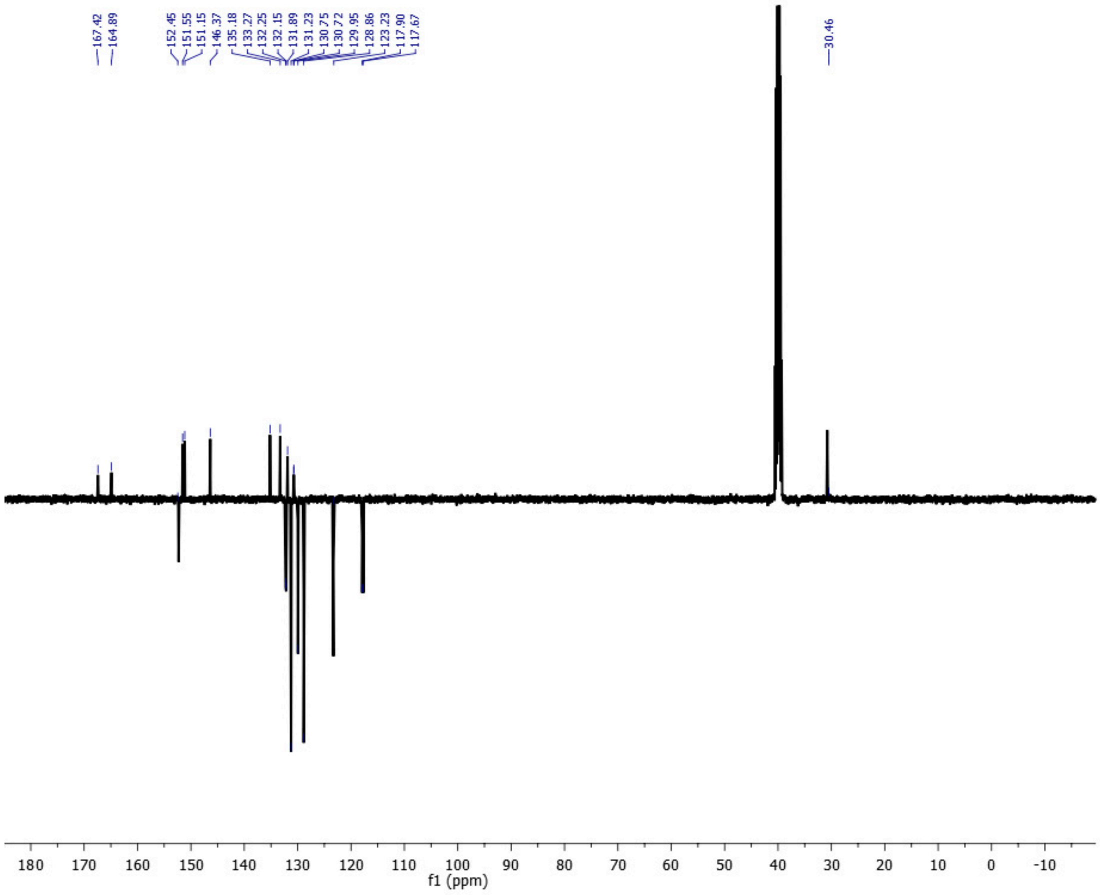

Figure S47. ^{13}C-NMR (APT) spectrum of compound 5b

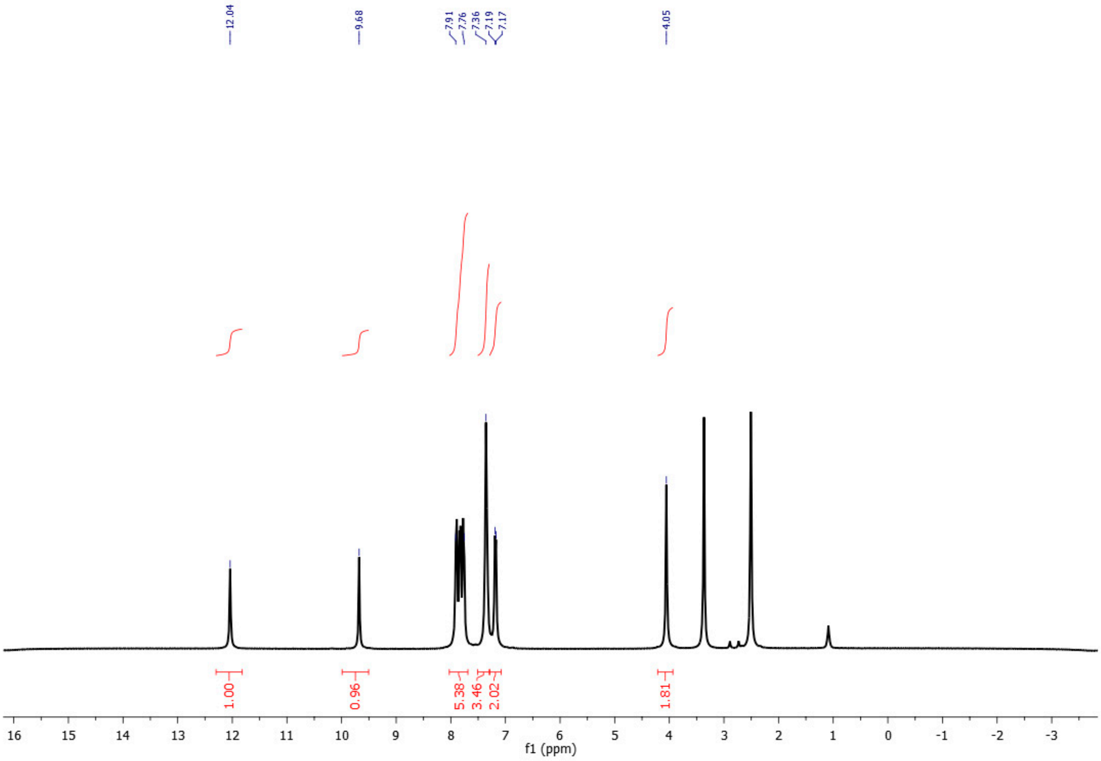

Figure S48. ^{1}H-NMR spectrum of compound 5c

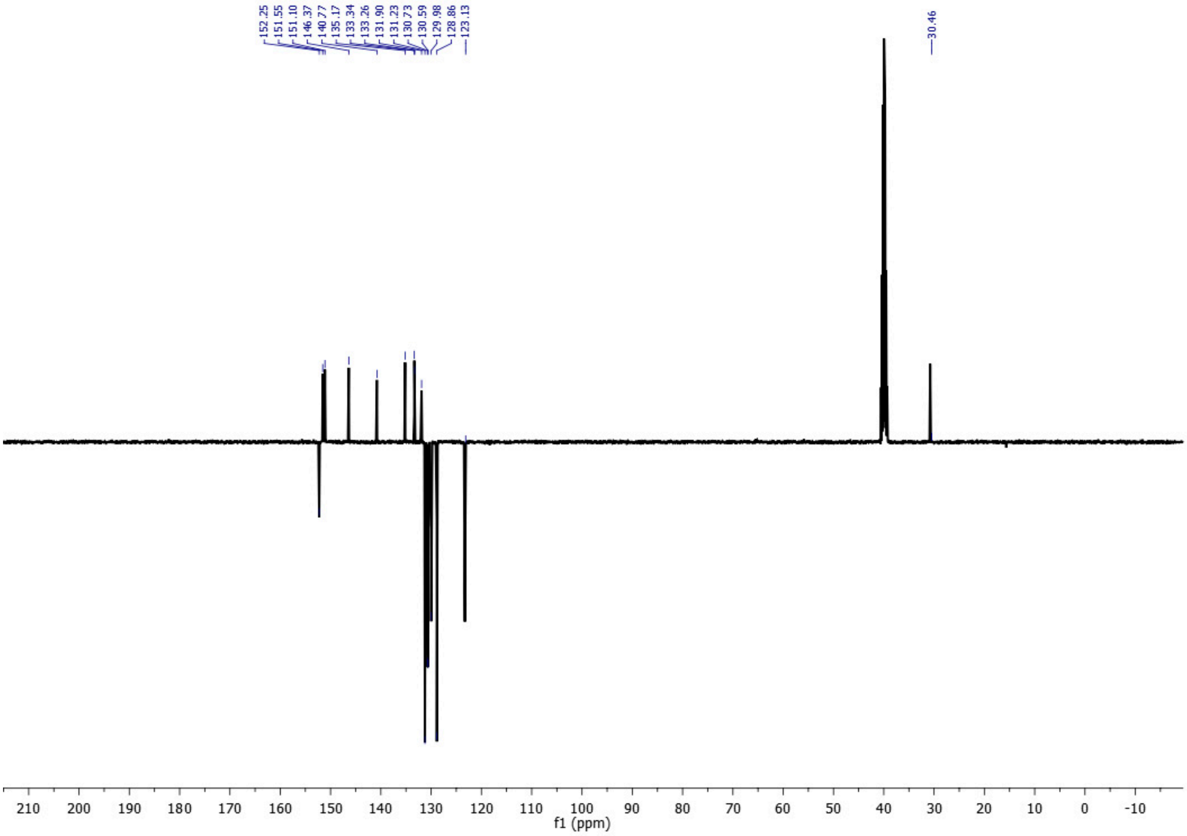

Figure S49. ^{13}C-NMR (APT) spectrum of compound 5c

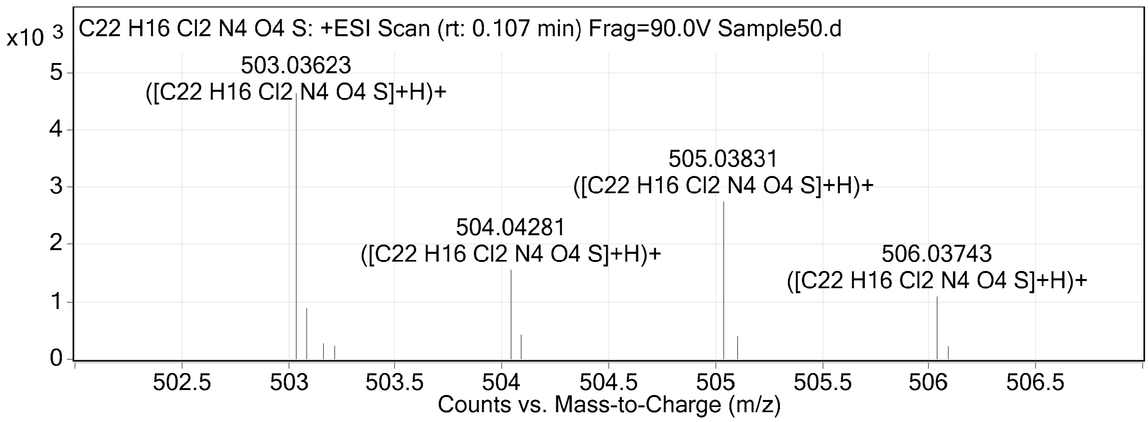

Figure S50. Q-TOF spectrum of compound 5c

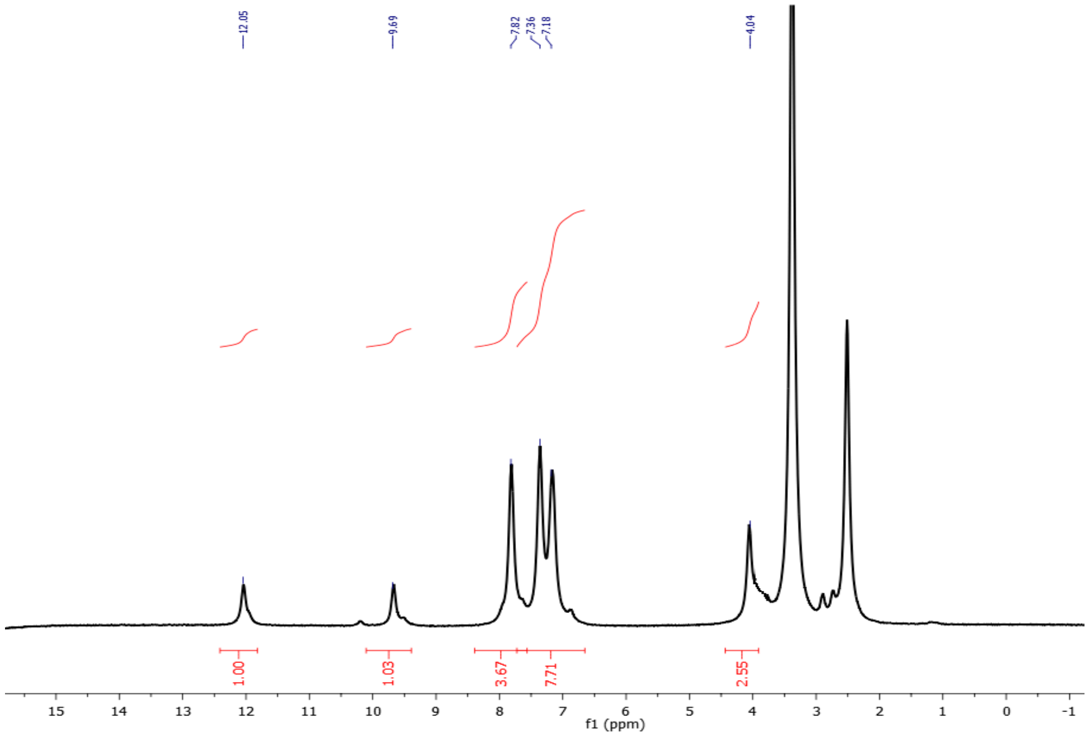

Figure S51. ^{1}H-NMR spectrum of compound 5d

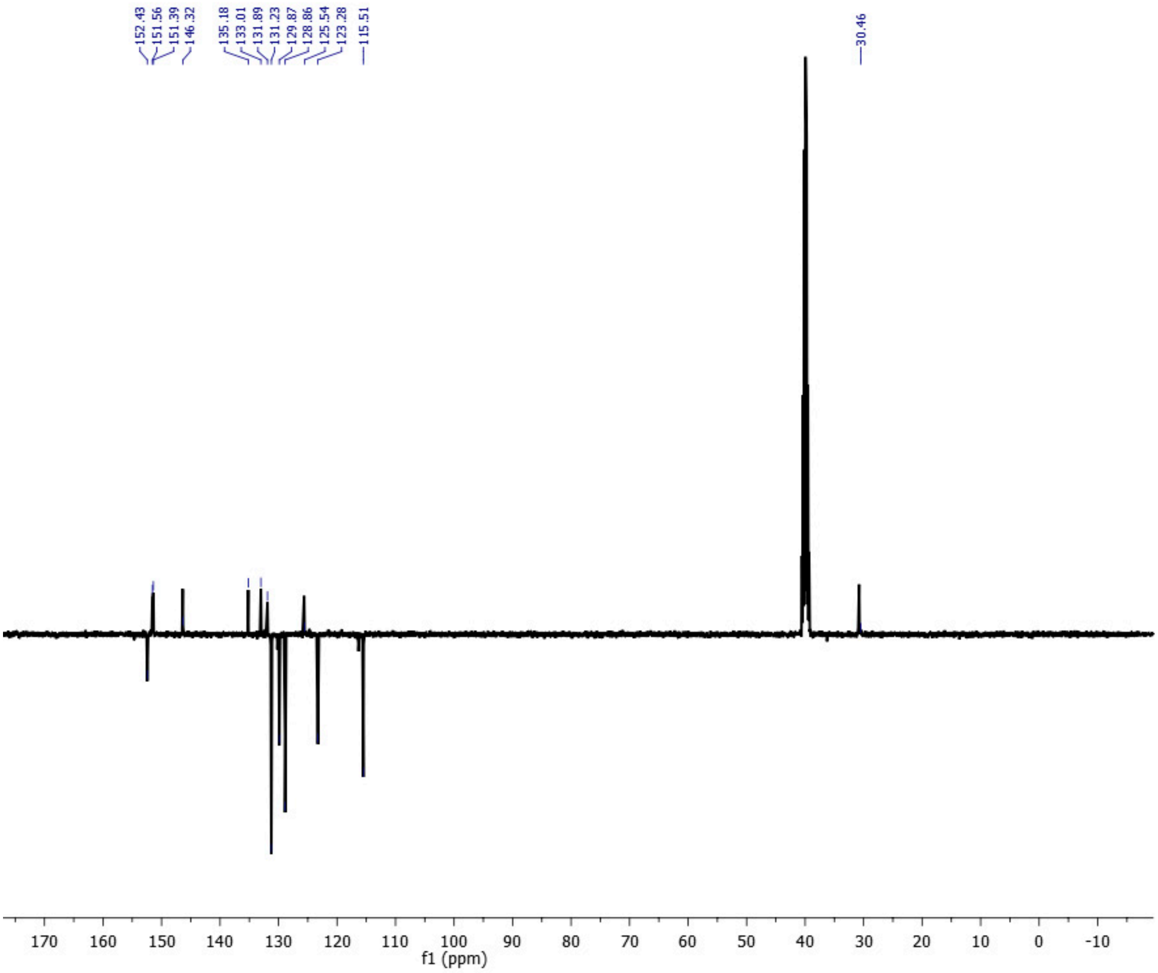

Figure S52. ^{13}C-NMR (APT) spectrum of compound 5d

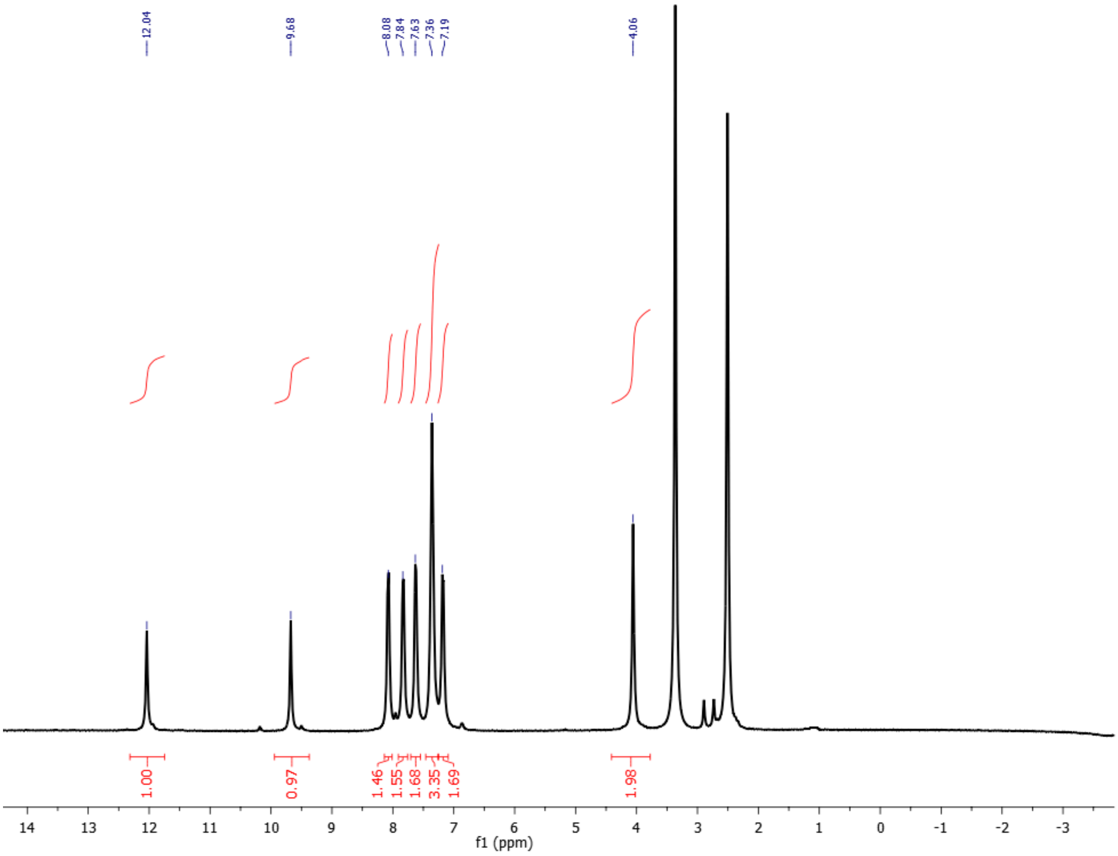

Figure S53. ^{1}H-NMR spectrum of compound 5e

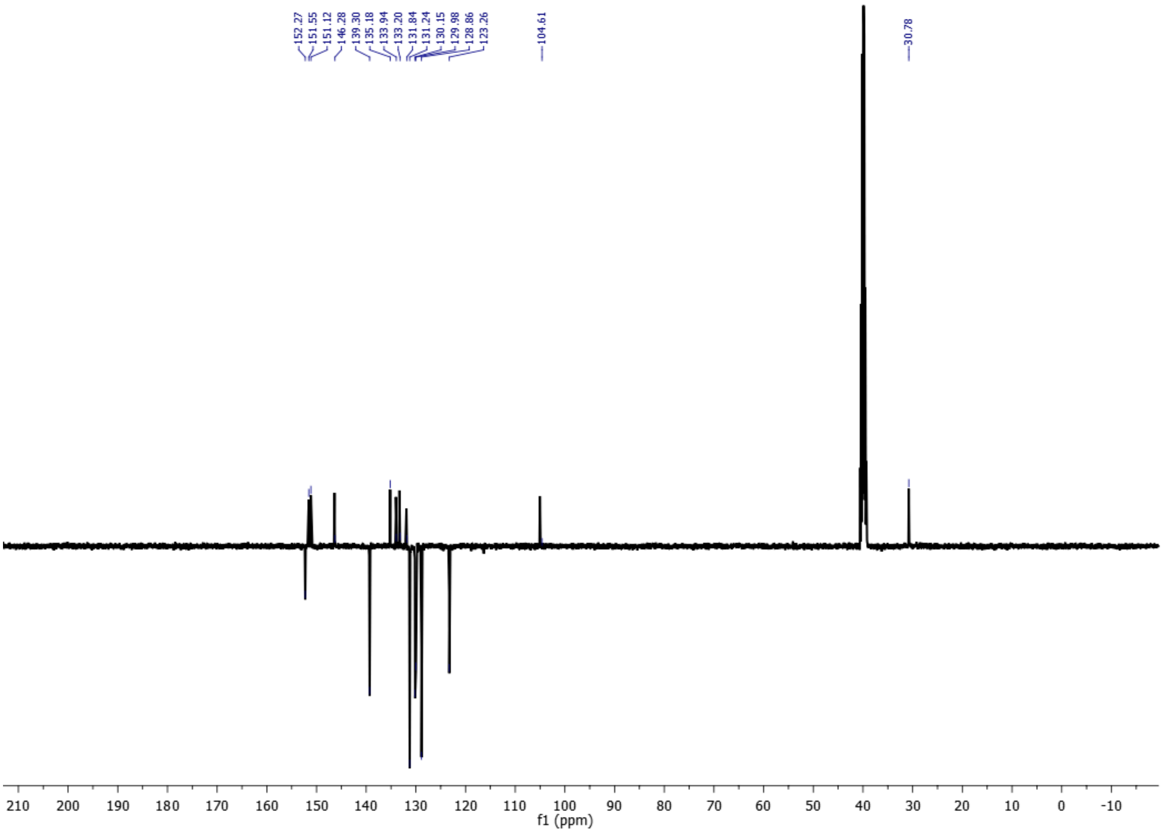

Figure S54. ^{13}C-NMR (APT) spectrum of compound 5e

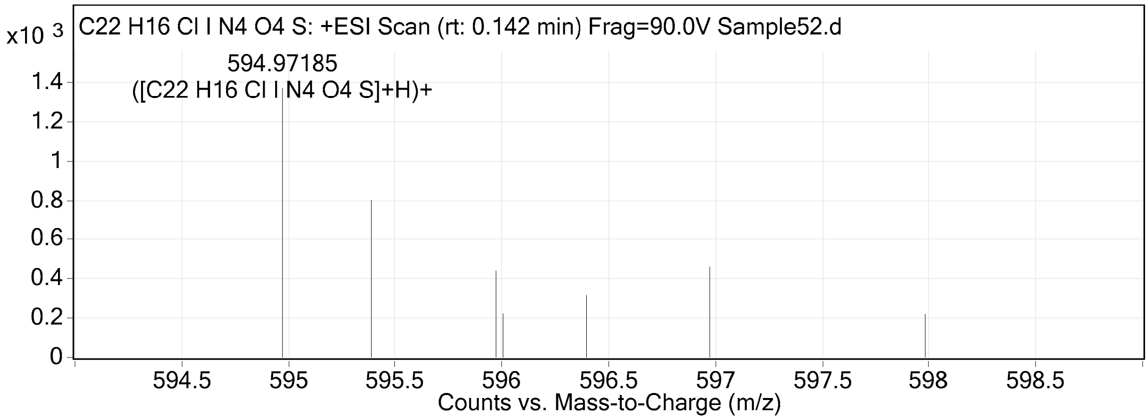

Figure S55. Q-TOF spectrum of compound 5e

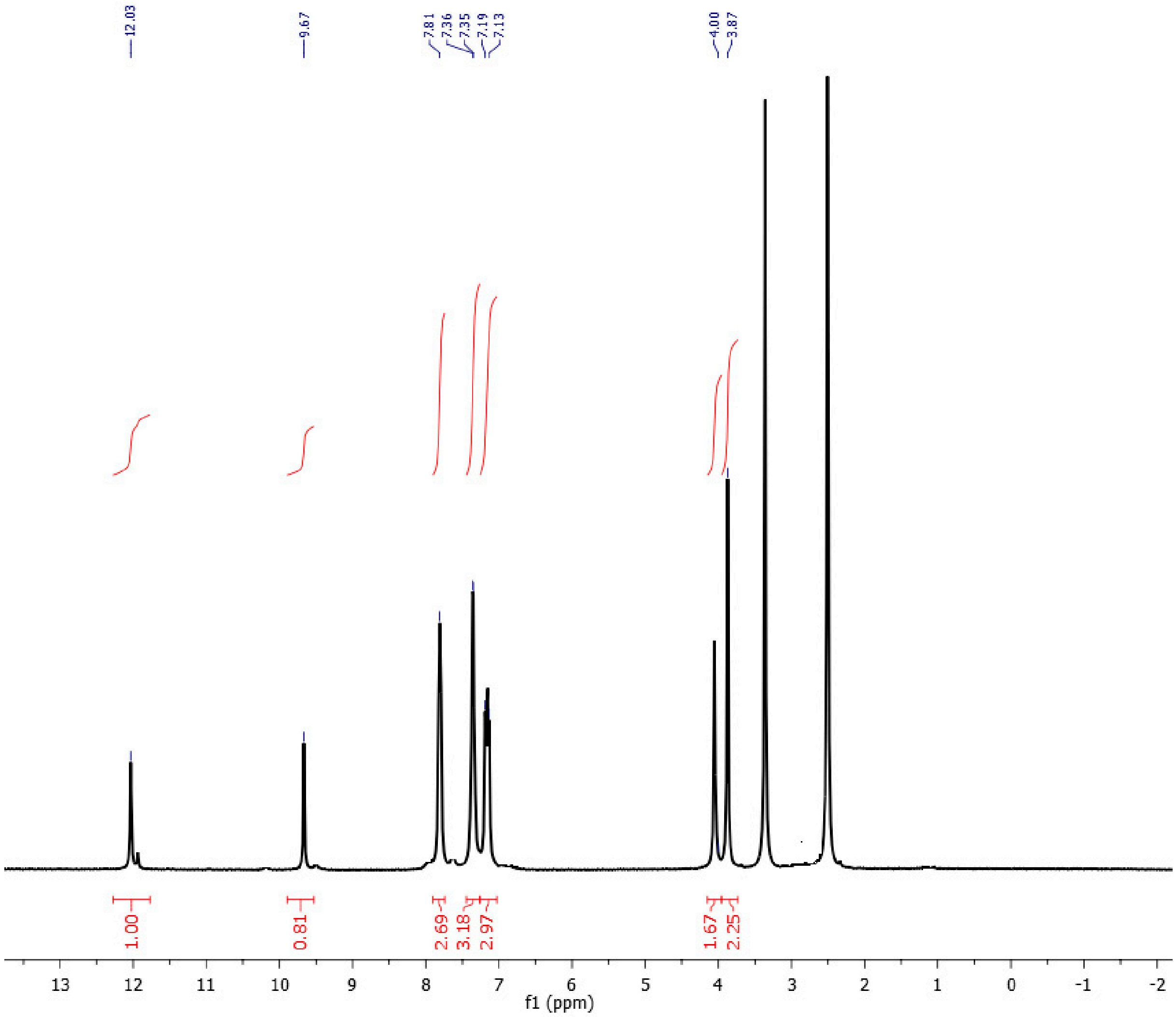

Figure S56. ^{1}H-NMR spectrum of compound 5f

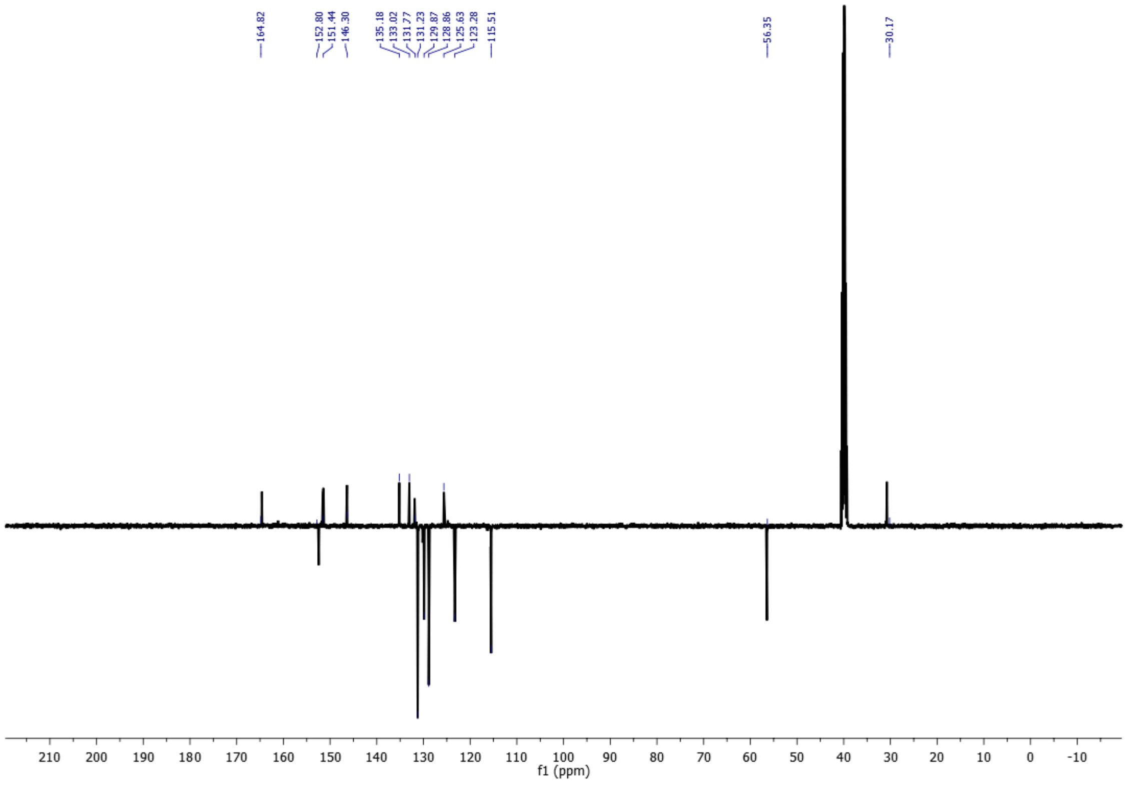

Figure S57. ^{13}C-NMR (APT) spectrum of compound 5f

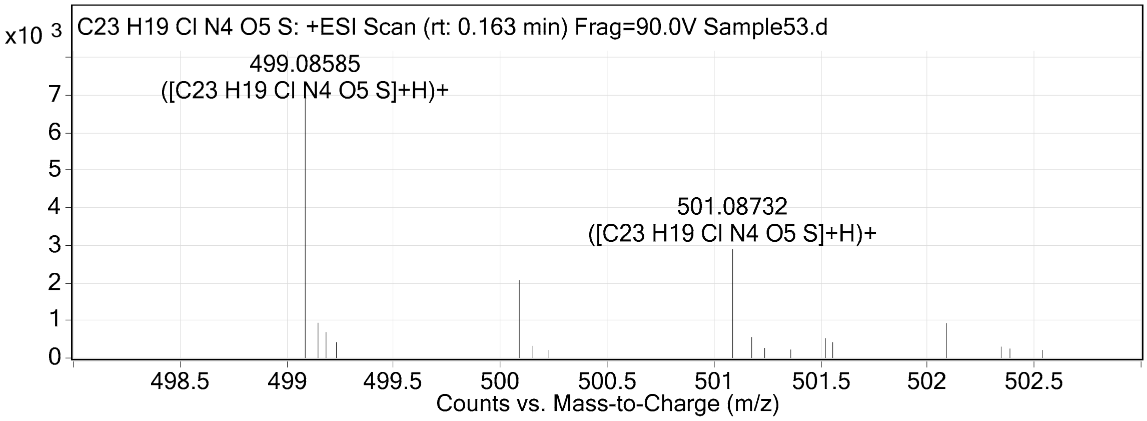

Figure S58. Q-TOF spectrum of compound 5f

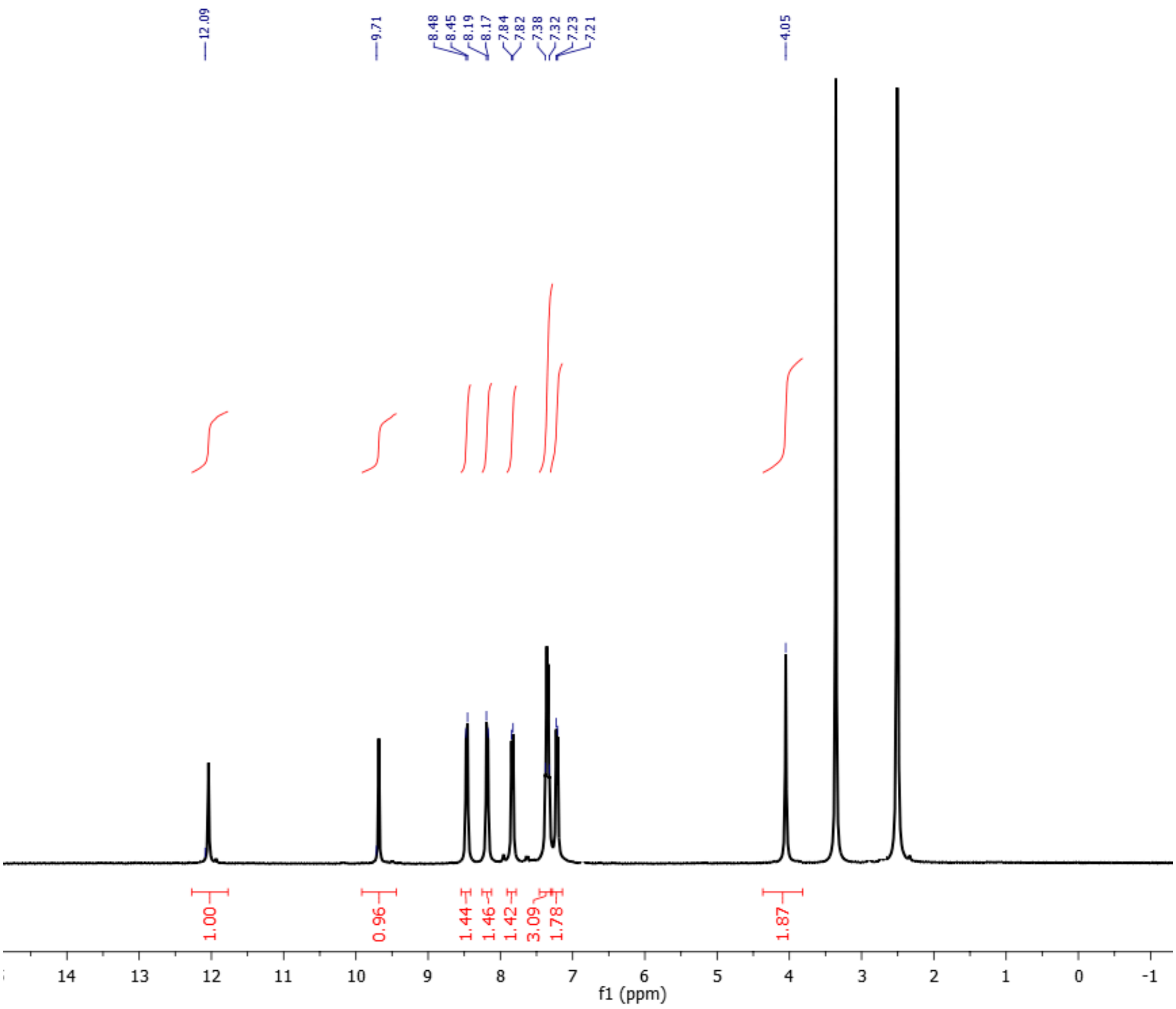

Figure S59. ^{1}H-NMR spectrum of compound 5g

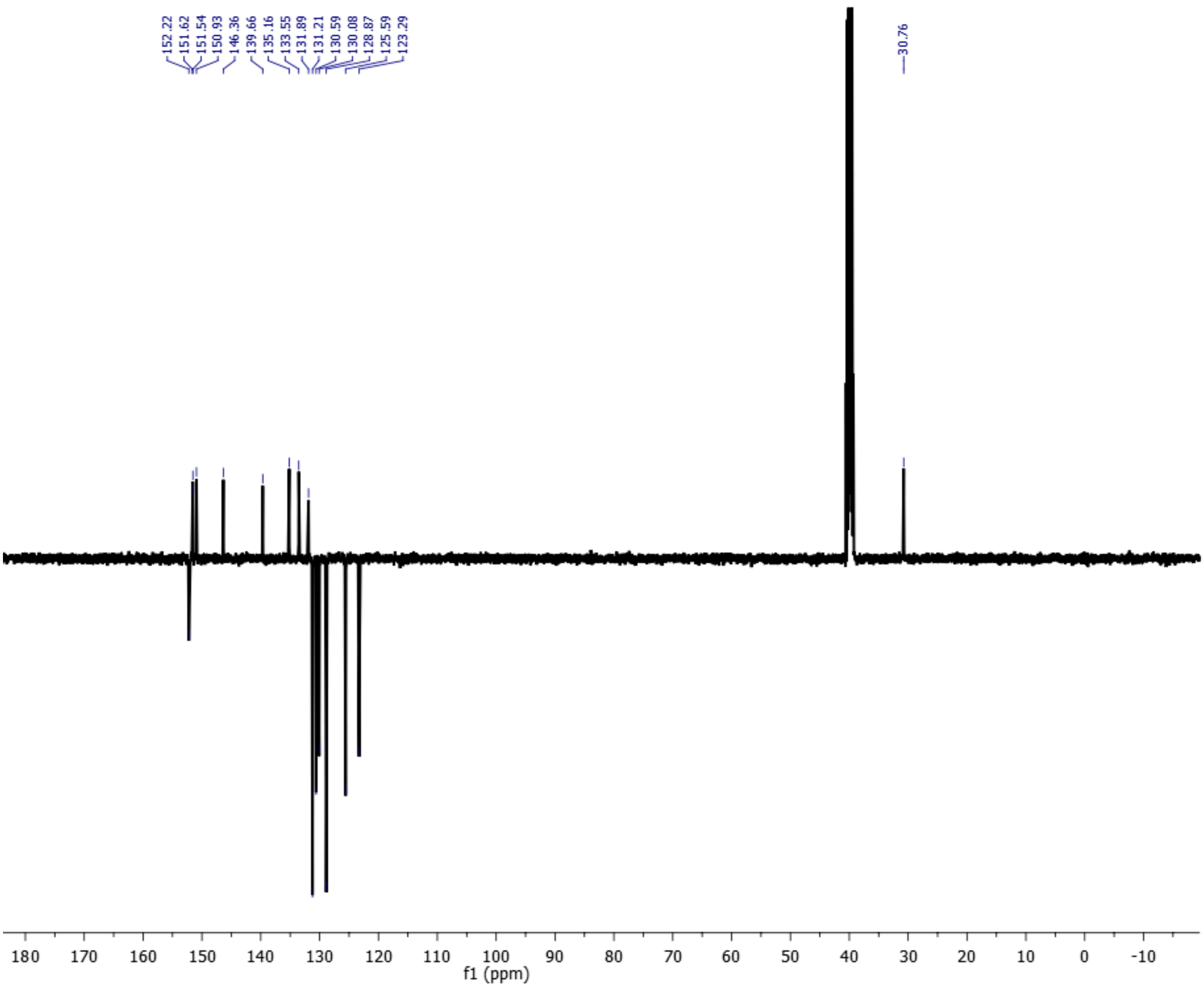

Figure S60. ^{13}C-NMR (APT) spectrum of compound 5g

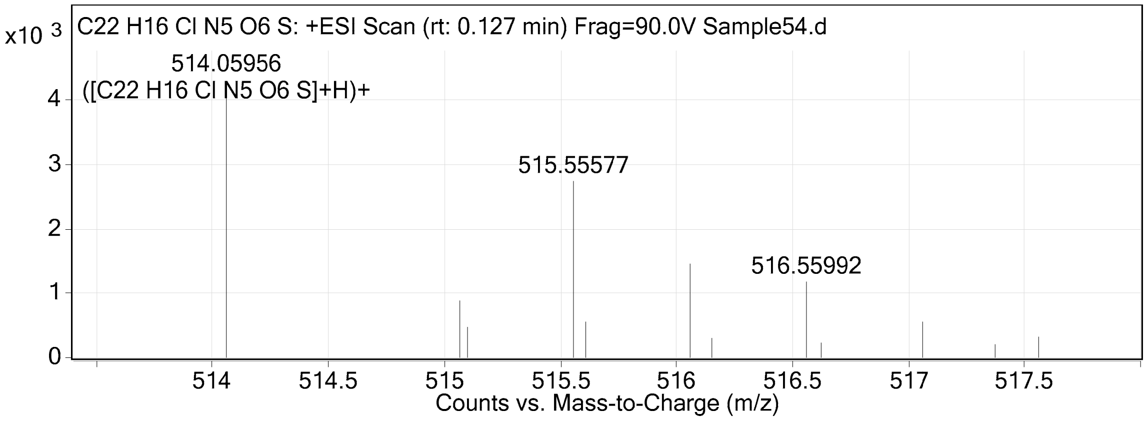

Figure S61. ^{1}H-NMR spectrum of compound 5g

AChE	GST
3a 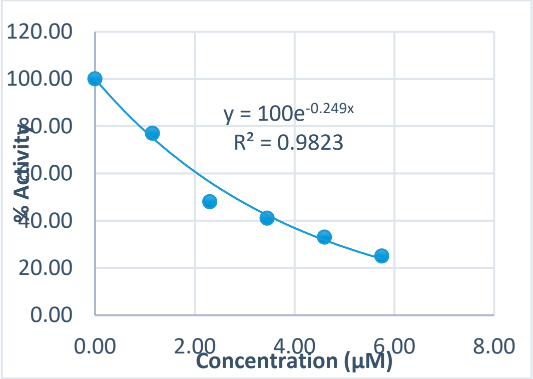	3a 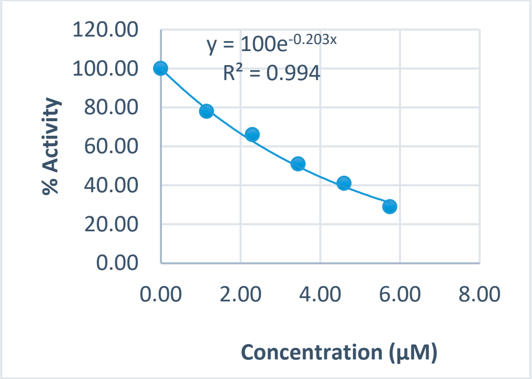
3b 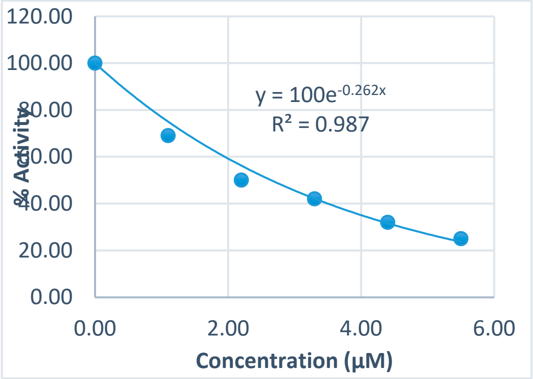	3b 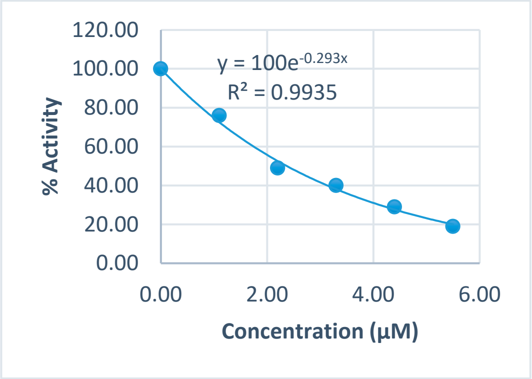
3c	3c

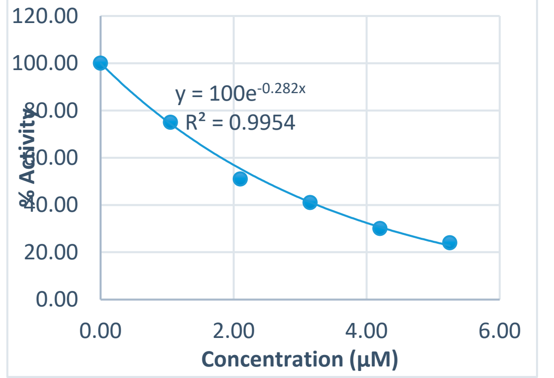

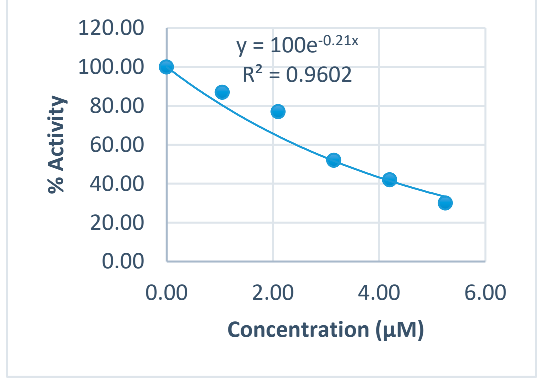

3d | 3d

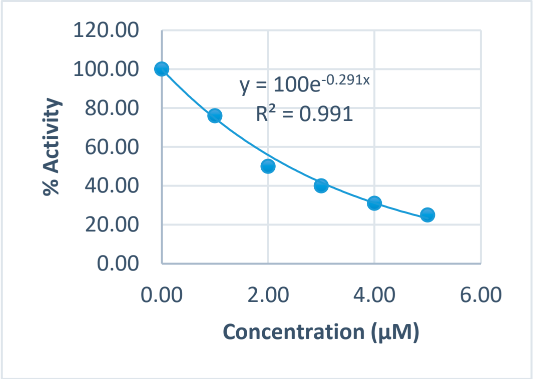

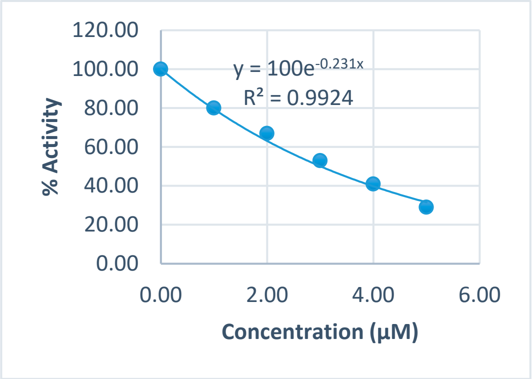

3e | 3e

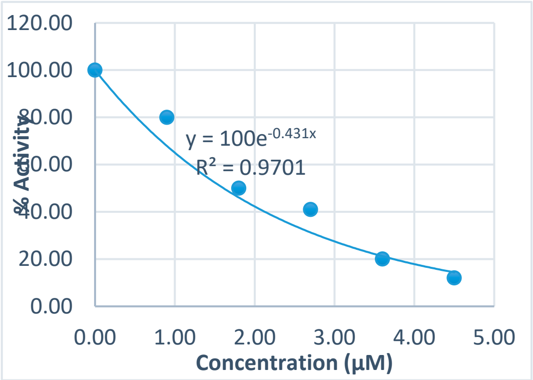

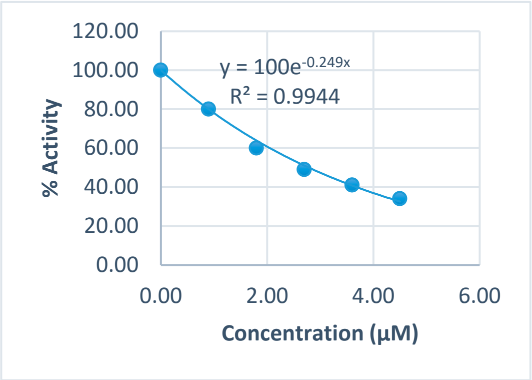

3f | 3f

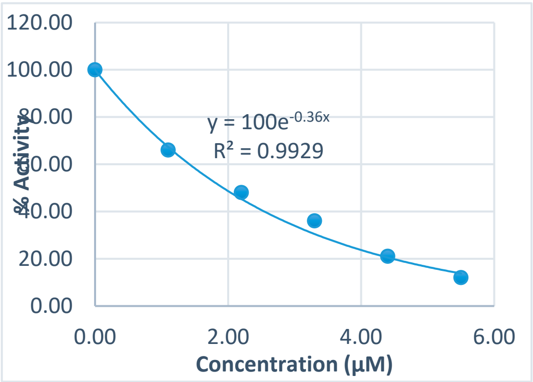

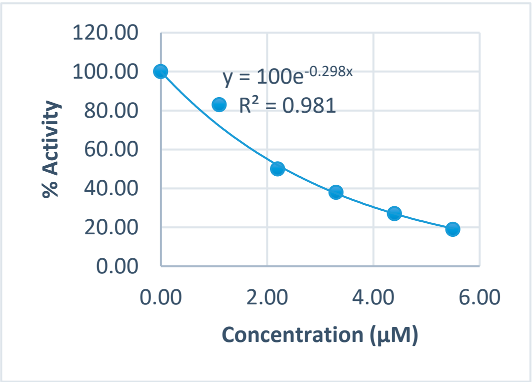

3g | 3g

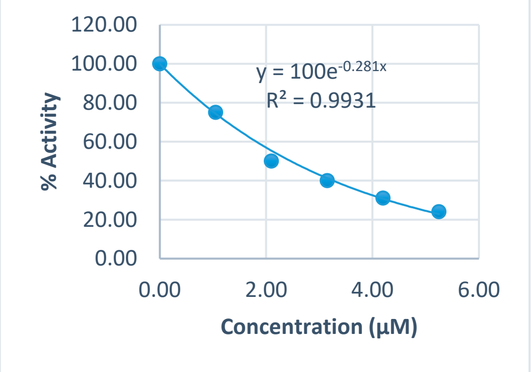

4a

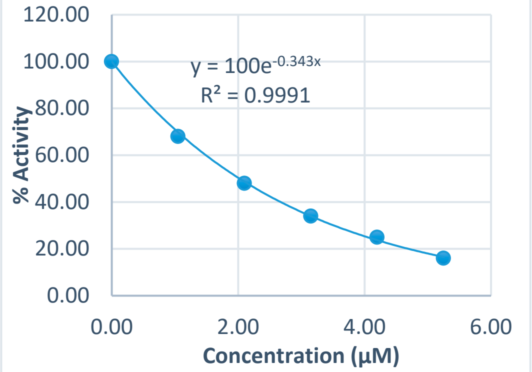

4a

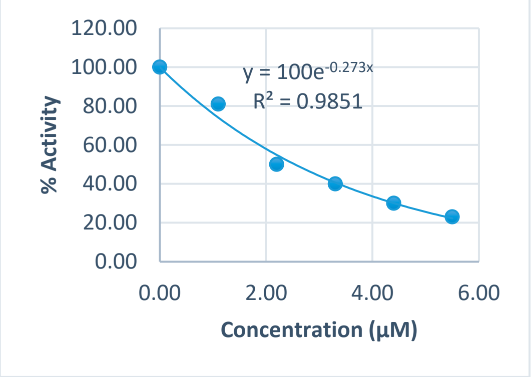

4b

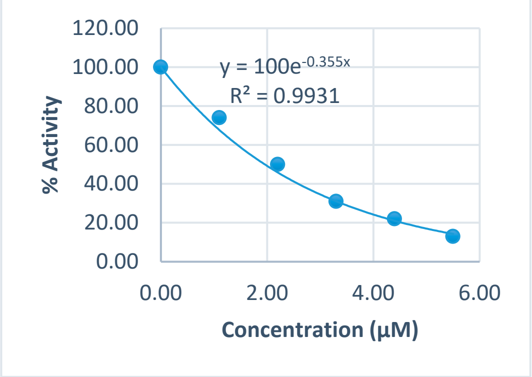

4b

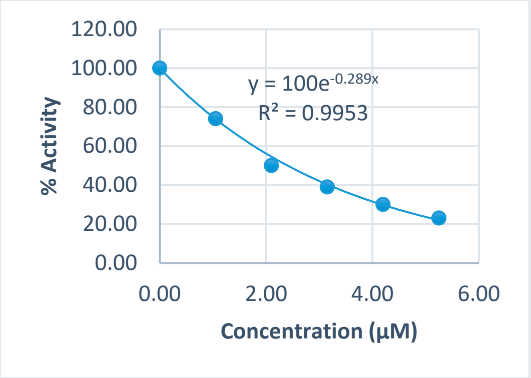

4c

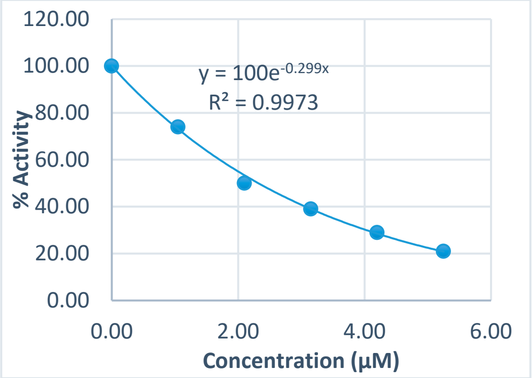

4c

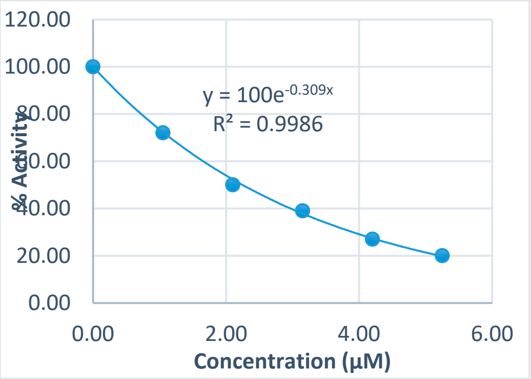

4d

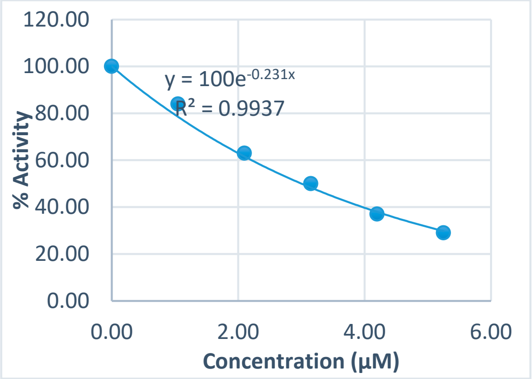

4d

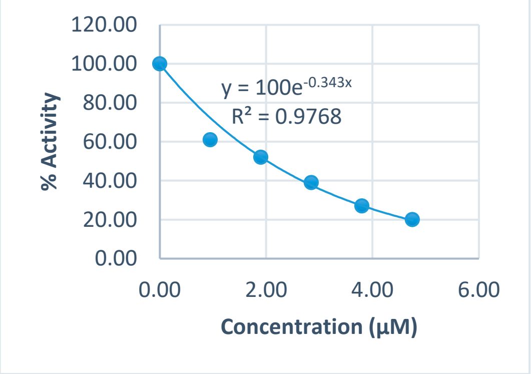	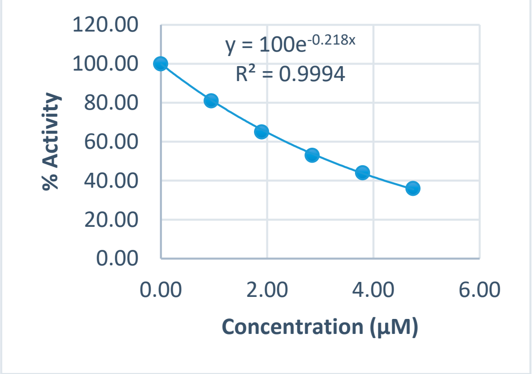
4e	4e
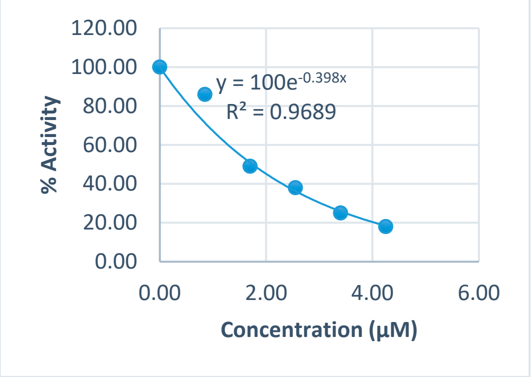	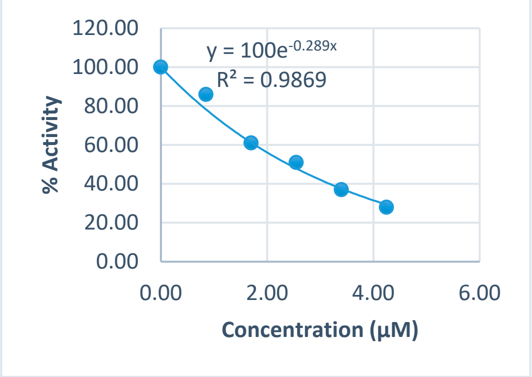
4f	4f
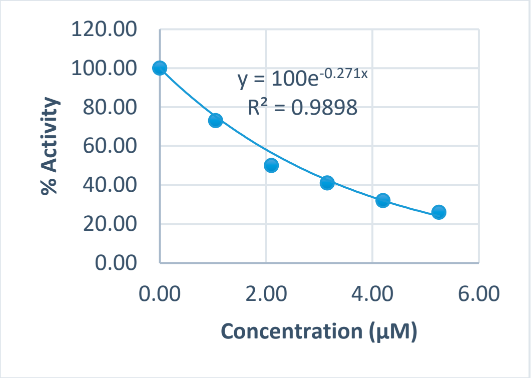	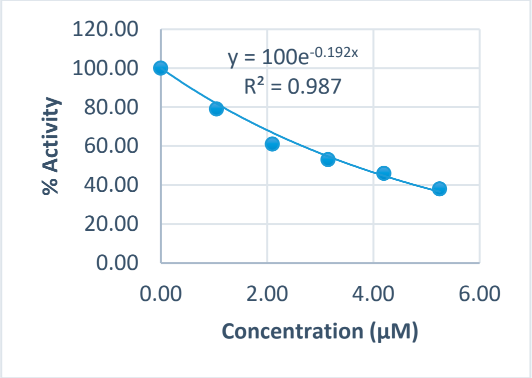
4g	4g
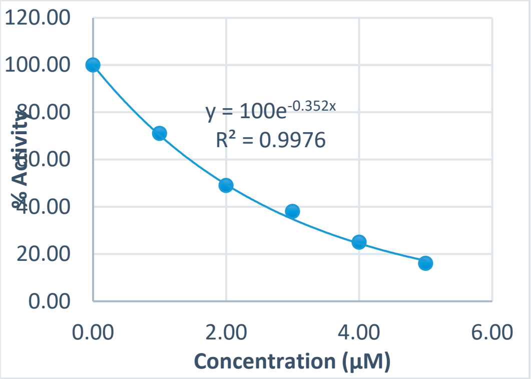	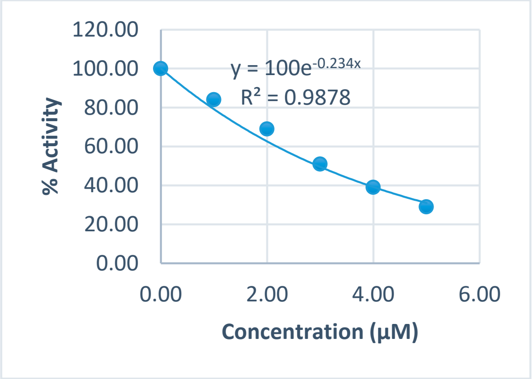
5a	5a

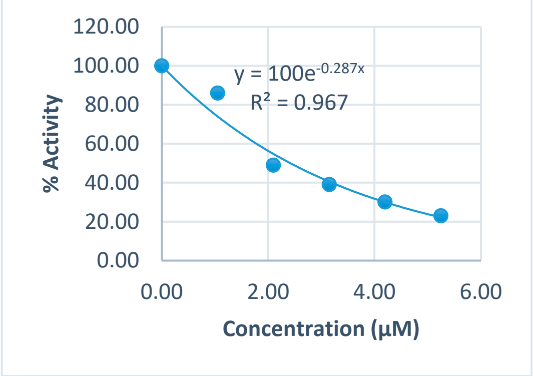

5b

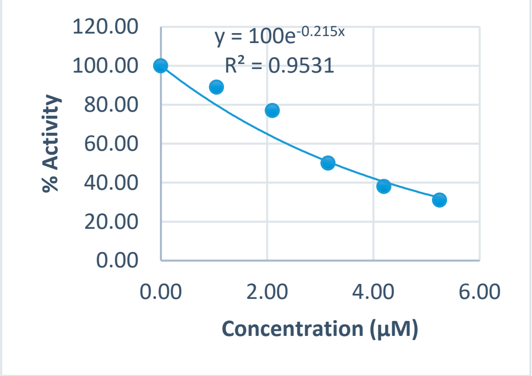

5b

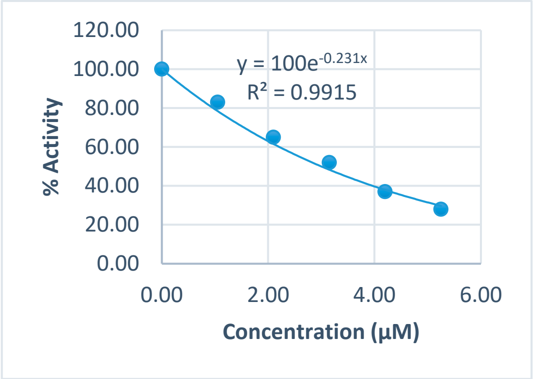

5c

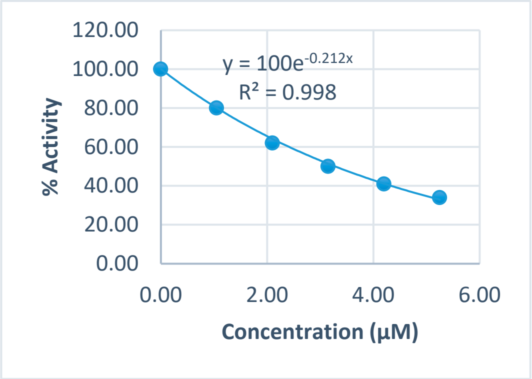

5c

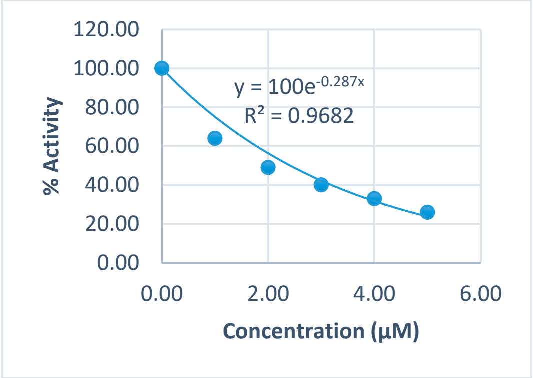

5d

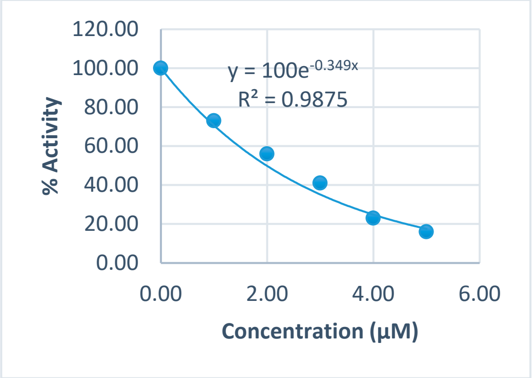

5d

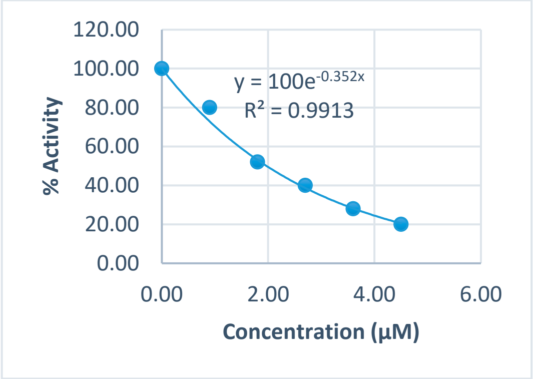

5e

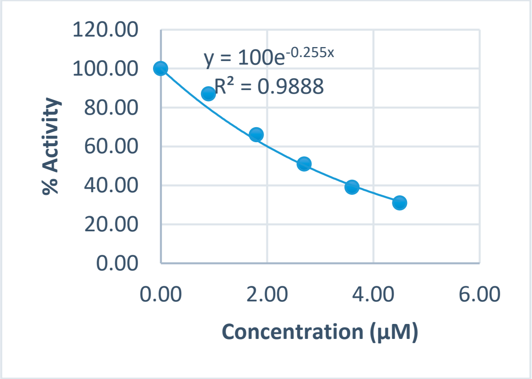

5e

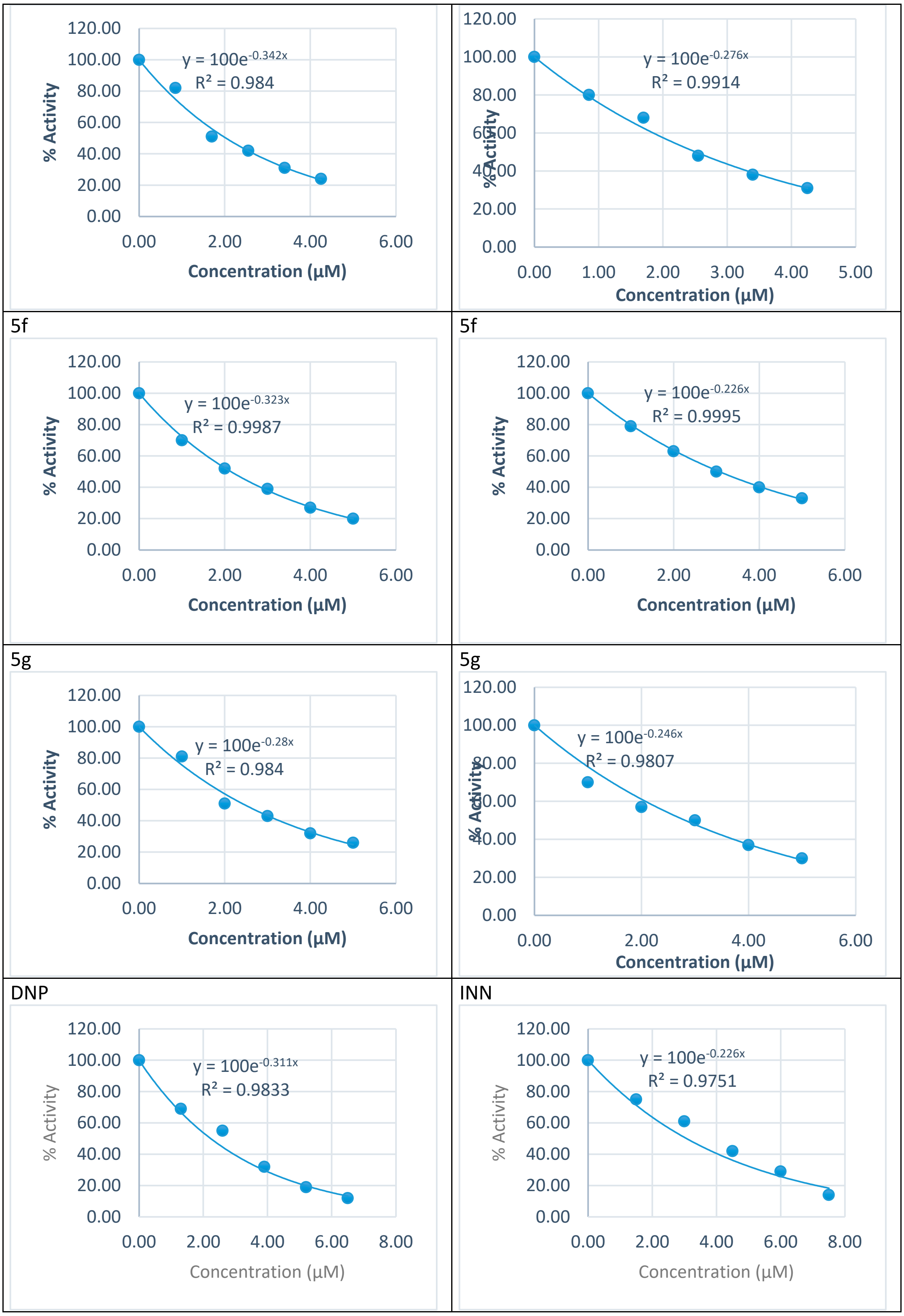

Figure S62. IC_{50} inhibition curves of the synthesized compounds and reference inhibitors against the investigated enzymes.
